# Regulation of apoptosis, ferroptosis, and pyroptosis mediated by acetylation

**DOI:** 10.1038/s41420-025-02859-1

**Published:** 2026-01-09

**Authors:** Weijie Lu, Yaoting Deng, Mengyang Liu, Yujie Hu, Kang Yang, Bowen Wang, Yanling Li, Gang Wang, Li Ma, Jiajia Liu, Yaohui Xie, Qianrong Li, Ai Liu, Xiubin Zhang, Ping Xie

**Affiliations:** 1 College of Integrated Traditional Chinese and Western Medicine, Gansu University of Traditional Chinese Medicine, Lanzhou, Gansu China; 2 School of Clinical Medicine, Lanzhou University, Lanzhou, Gansu China; 3 Department of Critical Care Medicine, The 940th Hospital of the People’s Liberation Army Joint Logistics Support Force, Lanzhou, Gansu China; 4 Cardiology, Gansu Provincial People’s Hospital, Lanzhou, Gansu China

**Keywords:** Acetylation, Oncogenesis

## Abstract

Acetylation is an important post-translational modification (PTM) of proteins and plays critical roles in multiple biological processes. The modes of cell death represent different pathways leading to the final outcome of “death” for the cell, with apoptosis, ferroptosis, and pyroptosis being the most common forms of cell death. In recent years, research has found that acetylation modifications influence various biological processes, ultimately playing a role in the regulation of apoptosis, ferroptosis, and pyroptosis. This article introduces the molecular effects of acetylation and enzyme/non-enzyme regulation, systematically summarizing the regulatory mechanisms of apoptosis, ferroptosis, and pyroptosis. It ultimately focuses on the genes and proteins associated with the regulation of apoptosis, ferroptosis, and pyroptosis, providing a comprehensive explanation of how acetylation regulates these processes. Given that the mode of cell death may be singular or coexist with two or more types in certain diseases, this article aims to conduct an in-depth analysis of the regulatory role of a specific PTM of proteins (acetylation) on different cell death pathways. Furthermore, it seeks to summarize potential key pathways or targets through which acetylation influences the interplay of various cell death mechanisms. By intervening in multiple cell death pathways, this study aims to provide insights for the prevention and treatment of tumors and cardiovascular diseases, both of which are closely related to outcomes associated with cell death.

## Facts

- ROS can simultaneously mediate apoptosis, ferroptosis, and pyroptosis, serving as one of the core factors that promote cell death.
- NAT10 catalyzes the acetylation modifications of tRNA, rRNA, and mRNA, regulating RNA stability, translation efficiency, and gene expression, thereby influencing the types of cell death that occur.
- The acetylated state of p53 can simultaneously promote the occurrence of apoptosis and ferroptosis.
- STAT3 and IL-1β can regulate both ferroptosis and pyroptosis, serving as significant mediators that influence these processes.

## Open questions

- Apoptosis, ferroptosis, and pyroptosis, whether independently or in combination, may play significant roles in the progression of diseases. Should we focus on and intervene in the effects of multiple forms of cell death on diseases? Could this further enhance the clinical efficacy of treatments for diseases closely associated with cell death, such as tumors and heart failure?
- NAT10, p53, microtubules, STAT3, and IL-1β all play roles in the acetylation regulation of apoptosis, ferroptosis, and pyroptosis. Under what circumstances do these factors act, and how do they regulate the interplay between different modes of cell death?
- NAT10, p53, microtubules, STAT3, and IL-1β play significant roles in the acetylation regulation of cell death. Is it possible to improve the clinical efficacy of diseases such as tumors and heart failure by intervening in the regulation of acetylating enzymes?

## Introduction

Acetylation is a type of post-translational protein modification that plays a crucial role in gene expression and protein function. With changes in dietary patterns, the incidence of metabolism-related diseases has been rising within the spectrum of human diseases, particularly obesity induced by high-fat diets. Metabolic state is closely linked to cellular acetylation, and the two are mutually regulated through key metabolites [1]. For example, acetyl-CoA serves both as a substrate for lipid synthesis and as a donor for histone acetylation, thereby connecting metabolism with epigenetic regulation and influencing cell fate [2]. At the same time, the levels of metabolites such as acetyl-CoA, NAD^+^, and acetylcarnitine can modulate the activities of acetyltransferases and deacetylases, making the cellular acetylation status a critical hub for sensing the body’s metabolic state [3].

Cell death plays a crucial role in both the developmental processes of organisms and the maintenance of homeostasis after birth. During the morphological development stage, as well as in sustaining the body’s stable state postnatally, cell death effectively eliminates damaged or no longer needed cells. At the same time, in the face of pathogen invasion, cell death can effectively curb the spread of the pathogen by inducing the demise of infected cells. Cell death can be induced by the genetically programmed suicide mechanisms of apoptosis, necroptosis, and pyroptosis, or it can be a consequence of dysregulated metabolism, as in ferroptosis [4].

In recent years, with the application of high-resolution mass spectrometry, research into post-translational modifications of proteins has become increasingly in-depth. This is particularly relevant in the current era, marked by a high prevalence of metabolic-associated diseases, where researchers have begun to focus more on the regulatory effects of acetylation modifications on the ultimate outcomes for cells, specifically their role in regulating modes of cell death. Whether it is the regulation of apoptosis by acetylation, or its regulation of ferroptosis or pyroptosis, numerous studies have emerged. However, the related reviews tend to focus more on the effects of post-translational modifications on specific types of cell death, such as the impact of post-translational modifications on ferroptosis or pyroptosis, and there has yet to be a discourse on how a particular modification affects multiple forms of cell death [5–8]. In fact, certain disease cells can simultaneously undergo apoptosis, ferroptosis, or pyroptosis. For example, in heart failure with preserved ejection fraction, there are studies related to apoptosis [9], as well as those concerning ferroptosis [10] and pyroptosis [11]. However, research has also indicated an inhibitory relationship between ferroptosis and pyroptosis [12]. Additionally, some articles have reported characteristics of cell pyroptosis signals triggered by ferroptosis in heart failure [13]. Therefore, we hypothesize that there may be crosstalk among the different modes of cell death. This article centers on the modification of acetylation. Based on a summary and overview of the pathogenesis of apoptosis, ferroptosis, and pyroptosis, it classifies and summarizes the regulatory mechanisms of acetylation on cell death pathways from the perspective of gene and protein-level acetylation modifications. The aim is to provide a relatively comprehensive discussion on how acetylation modifies the regulation of cell death modes, while also exploring common pathways through which acetylation influences these three types of cell death. This approach seeks to offer insights into the interregulatory mechanisms of cell death modes mediated by acetylation.

## Overview of acetylation

### Concept and classification of acetylation

Acetylation refers to lysine acetylation, which is an evolutionarily conserved post-translational modification of proteins (PTM) present in both prokaryotes and eukaryotes. In 1964, Vincent Allfrey and his colleagues demonstrated that histones undergo acetylation by having acetyl groups (–COCH₃) introduced at the ε-amino group of lysine residues [14]. Since then, acetylation has emerged as a regulatory form of PTM, marking the beginning of research into its roles in gene expression, transcriptional regulation, and protein functionality. In 2006, the combination of acetylated peptide immunoaffinity enrichment and high-resolution mass spectrometry enabled the identification of hundreds of acetylation sites [15]. During this period, researchers discovered that, in addition to histones, large-scale proteomic studies have established that across diverse organisms ranging from bacteria to mammals, there exist thousands of highly conserved non-histone protein lysine acetylation sites—modifications that are not only ubiquitous but also subject to dynamic and precise regulation [16]. Thus, the study of acetylation has been divided into two categories: histone acetylation and non-histone acetylation.

### Mechanism of action and molecular effects of acetylation

#### Mechanism of acetylation

In acetylation modification, although the mass of the acetyl group is only 42 Da, the acetylation of the ε-amino group results in the neutralization of the inherent positive charge of lysine residues. This change in charge can disrupt the electrostatic interactions between the modified residue and other macromolecules, thereby exerting biological effects. Previous studies have typically demonstrated that regulatory effects are exerted through various mechanisms, including altering enzyme activity, regulating protein degradation, influencing protein-protein interactions, modulating DNA synthesis, controlling subcellular localization, and engaging in crosstalk with other PTMs [17].

#### Molecular effects of histone acetylation

##### Regulation of genes by histone acetylation

Lysine acetyltransferases (KATs) catalyze the transfer of acetyl groups to lysine residues, representing a class of enzymes responsible for acetylation. Similar to histone methylation, their recruitment to chromatin is believed to depend on transcription factors or pre-existing chromatin modifications [18]. However, the acetylation of histone residues can, in turn, influence transcription by altering chromatin structure or by recruiting acetyl-lysine reading proteins.

**Histone acetylation disrupts interactions between nucleosomes:** Research indicates that the side chain of the lysine residue at position 16 of histone H4 in a nucleosome protrudes into an acidic cavity formed by the glutamic acid residue at position 61 and the aspartic acid at position 90, as well as the glutamic acid residue at position 92 of the histone H2 from the adjacent nucleosome [19]. When H4K16 undergoes acetylation, it disrupts the interaction between the H4 tail of one nucleosome and the acidic cavity of the neighboring nucleosome [20]. Consistent with existing research, H4K16 acetylation reduces the proximity of H2A-H2B and H4 in vivo [21]. The disruption of nucleosome interactions can eliminate the folding of nucleosomes into higher-order arrays in vitro, thereby achieving the regulation of gene expression and the stability of chromatin structure.

**Acetylated histones recruit reading proteins:** Histone acetylation promotes transcription by recruiting proteins containing bromodomains. The histone code hypothesis posits that histone modifications serve as local information carriers, interpreted by chromatin-binding proteins known as “readers,” which can distinguish between modified and unmodified nucleosomes [22]. It is known that various protein domains possess the ability to specifically recognize or “read” acetylated lysine residues. These domains include bromodomains such as Yaf9, ENL, AF9, Taf14, and Sas5 (YEATS domain), as well as tandem plant homology domains known as PHDs, also referred to as double PHD finger domains [23]. Bromodomain-containing protein 4 (BRD4) and Transcription Factor IID Subunit 1 (TAF1) are proteins that contain bromodomains. Sequencing data obtained from chromatin immunoprecipitation showed that after just 5 min of treatment with the KAT3A and KAT3B inhibitor A-485, the binding of TAF1 to super-enhancers and promoters was reduced. Additionally, the loss of TAF1 binding induced by A-485 was associated with the elimination of transcription initiation in enhancer-regulated genes [24], indicating that a decrease in acetylation levels can inhibit transcription. Moreover, the absence of the bromodomain and extra-terminal domain (BET) in the BRD4 protein weakens the capability of RNA polymerase II to transition from a paused state near the promoter to transcription elongation, thereby inhibiting transcription [25, 26]. These two examples illustrate that histone acetylation promotes transcription by recruiting proteins that contain bromodomains.

**Histone acetylation also facilitates the recruitment of ribosome remodeling factors:** Ribosome remodeling factors are ATP-dependent enzymes that can slide, evict, or alter the spacing of ribosomes, thereby exposing specific DNA segments [27]. Histone acetylation can regulate chromatin accessibility to modulate transcription.

#### Molecular effects of non-histone acetylation

##### Gene transcription

Protein acetylation is a major regulatory factor in gene transcription. Most typical KATs are located in the nucleus and function as transcription co-activators. Similarly, nearly all proteins that bind to acetylated lysine with a bromodomain are also localized in the nucleus, many of which are directly involved in transcription. The tumor suppressor protein p53 is the first transcription factor identified to undergo acetylation [28]. The acetylation of p53 regulates its DNA binding, stability, and interactions with other proteins, and is closely related to the activation of p53 target genes in response to cellular stress [29]. Overall, acetylation involves the regulation of over 100 non-histone transcription regulatory proteins, including transcription factors, transcription co-activators, and nuclear receptors [30]. Therefore, regulating gene transcription is one of the primary functions of non-histone acetylation.

##### Cell cycle

During the process of DNA replication, sister chromatids are paired together by adhesion complexes until they are separated during mitosis. The ATPase head of Structural Maintenance of Chromosomes 3 (SMC3) is a key component of the cohesin complex, and it is acetylated at two conserved DNA-sensing residues, Lys105 and Lys106 [31–33]. Once SMC3 is loaded onto DNA, its acetylation locks the cohesin ring, thereby establishing stable cohesion between sister chromatids. In mammalian cells, SMC3 undergoes acetylation by Establishment of Sister Chromatid Cohesion 1 (ESCO1), followed by acetylation by Establishment of Sister Chromatid Cohesion 2 (ESCO2). Combined depletion of ESCO1 and ESCO2 leads to severe defects in sister chromatid cohesion, resulting in cell lethality [34]. Interestingly, the cohesin complex may be released in an acetylated form during early prophase and the prophase without mitosis, while HDAC8-dependent deacetylation is a prerequisite for the dissolution of the released complex [35]. This indicates that although the acetylation of SMC3 stabilizes the cohesion of chromatid monomers, SMC3 can also be released from chromatin through a deacetylation-independent mechanism. In addition, acetylation also regulates several other key cell cycle regulators, including protein kinase BUBR1, Aurora kinase A, Aurora kinase B, Cyclin-Dependent Kinase 1 (CDK1), CDK2, and PLK4, to modulate the cell cycle [30].

##### DNA damage repair

Ataxia Telangiectasia Mutated (ATM) is a critical regulatory factor in the repair of DNA double-strand breaks (DSB). TIP60 acetylates and activates ATM in response to DNA damage, and the inactivation of TIP60 renders cells sensitive to ionizing radiation [36]. Furthermore, the deacetylation of ATM by SIRT7 is a prerequisite for the dephosphorylation of ATM by its phosphatase WIP1, thereby facilitating DNA damage repair through the suppression of ATM phosphorylation [37]. Acetylation can also regulate the choice between the double-strand break (DSB) repair pathways of non-homologous end joining (NHEJ) and homology-directed repair (HDR) by promoting the recruitment of TP53-binding protein 1 (53BP1) to sites of DNA damage through the modulation of NHEJ [38]. Acetylation regulates proteins involved in the Base Excision Repair (BER) and Nucleotide Excision Repair pathways. For instance, DNA damage-induced Apurinic/Apyrimidinic Endonuclease 1 (APE1) is a key enzyme in BER, where acetylation inhibits its interaction with XRCC1 and reduces APE1 activity. The deacetylation catalyzed by SIRT1 restores APE1 function [39]. p62 is an autophagy adapter that accumulates in the nucleus in response to oxidative stress. It is acetylated by hMOF and subsequently deacetylated by SIRT7. The acetylated p62 is recruited to chromatin. p62, enriched in chromatin, directly interacts with the key enzyme APE1 in the BER pathway, enhancing its endonuclease activity and thereby promoting BER and cell survival [40].

##### Cell signaling

The acetylation of proteins associated with signal transduction also affects their function, such as the acetylation of Rapamycin-Insensitive Companion of mTOR (RICTOR) by p300, which increases mTORC2-mediated phosphorylation of AKT [41].

##### Protein folding

Chaperone-assisted protein folding is essential for achieving the functionally mature state of proteins. Heat Shock Proteins (HSPs) represent a major class of chaperone proteins in eukaryotes and are targets for acetylation; these proteins include HSP10, HSP70, HSP90, and HSPA5, among others. The acetylation of HSP90 affects its interaction with the critical co-chaperone p23, resulting in the loss of chaperone activity [42]. Acetylation of HSP90 reduces its interaction with endothelial nitric oxide synthase (eNOS), leading to decreased nitric oxide production and subsequent hepatic sinusoidal endothelial dysfunction. Specific overexpression of HDAC6 in hepatic endothelial cells can induce the deacetylation of HSP90, restoring the interaction between HSP90 and eNOS, and improving alcohol-induced liver injury in mice. HSP90 is crucial for the proper folding of many signaling-related proteins, and inhibiting HSP90 can synergistically suppress the proliferation of cells expressing oncogenic kinases when used in conjunction with KDAC inhibitors [43].

##### Cytoskeletal structure

Microtubules are composed of α-tubulin and β-tubulin and are an essential component of the cytoskeleton in eukaryotic cells. α-tubulin is acetylated at Lys40 by the cytoplasmic acetyltransferase TAT1 and deacetylated by the cytoplasmic deacetylase HDAC6 [44]. Research indicates that increased consumption of TAT1 raises the frequency of mechanically induced microtubule rupture, suggesting that acetylation enhances the mechanical resilience of microtubules to ensure the durability of long-lived microtubules [45]. Acetylation also regulates another major cytoplasmic protein, cortactin, which binds to F-actin and contributes to the organization of the actin cytoskeleton and cell migration. Cortactin can be acetylated by CREB-binding protein (CBP) and p300, with acetylated cortactin primarily localized in the nucleus [46]. Acetylation reduces the binding of corticoid proteins to KEAP1 and inhibits cell migration, while HDAC6-dependent, Sirtuin 2, and Sirtuin 1-dependent deacetylation promote cell motility [47].

##### Protein aggregation

The accumulation of protein aggregates is associated with various neuropathologies. Some aggregation-prone proteins are acetylated, including huntingtin, tau protein, superoxide dismutase 1 (SOD1), and TDP-43, which affects their aggregation. For example, the acetylation of TDP-43 associated with amyotrophic lateral sclerosis weakens its RNA binding and promotes the accumulation of insoluble hyperphosphorylated forms of TDP-43 [48]. The acetylation-mimicking mutant TDP-43-K145Q promotes TDP-43 phosphorylation, ubiquitination, and aggregation [49].

##### RNA processing and stability

Acetylation regulates various steps of post-transcriptional RNA processing, including pre-mRNA splicing and polyadenylation, as well as the degradation of polyadenylated mRNA (mRNA decay). The acetylation of the Cleavage Factor Im 25 kDa Subunit (CFIm25) is a component of the cleavage factor Im complex, and poly(A) polymerase (PAP) inhibits mRNA polyadenylation through two mechanisms [50]. First, CFIm25 and PAP interact directly, and the acetylation of CBP in their interaction region inhibits their binding. Secondly, the acetylation of PAP leads to its export to the cytoplasm. Furthermore, CBP and p300 activate CCR4-related factor 1 through acetylation, a process that promotes mRNA degradation [51]. In contrast, the inhibition of HDAC1 and HDAC2 may induce widespread mRNA decay in mammalian and Drosophila melanogaster cells by enhancing protein acetylation.

##### Autophagy

Acetyltransferases CBP, p300, and TIP60, as well as the deacetylases HDAC6 and SIRT1, are important regulatory factors in autophagy. Depending on the target protein, acetylation can enhance or inhibit autophagy. For example, nutrient starvation induces the activation of glycogen synthase kinase 3, which phosphorylates and activates TIP60. TIP60 then acetylates and stimulates the kinase ULK1, which is essential for autophagy [52]. The activity of CBP and p300 is regulated by the Mammalian Target of Rapamycin Complex 1 (mTORC1). Under nutrient-rich conditions, mTORC1 phosphorylates the carboxy-terminal serine residues on p300, thereby alleviating its self-inhibition, inhibiting autophagy, and promoting lipogenesis [53]. The consumption of nutrients can lead to the relocalization of p300 from the cytoplasm to the nucleus, thereby reducing the acetylation of mTORC1, which in turn decreases the activity of mTORC1 and activates autophagy [54]. P300 acetylation of the key autophagy factors ATG5, ATG7, microtubule-associated protein light chain 3 (LC3; Atg8 in yeast), and ATG12 inhibits autophagy [55]. The UVRAG complex, composed of Vacuolar Protein Sorting 34 (VPS34; also known as PIK3C3), Vacuolar Protein Sorting 15 (VPS15; also known as PIK3R4), Beclin 1, and UVRAG, plays a crucial role in the maturation of autophagosomes by promoting the fusion of autophagosomes and lysosomes. The interaction with the Run domain of Beclin 1 and the interaction with the cysteine-rich protein Rubicon inhibit the function of the UVRAG complex [56]. Acetylation of Beclin 1 promotes the recruitment of Rubicon to the UVRAG complex, thereby inhibiting the maturation of autophagosomes [57].

SIRT1 interacts with ATG5, ATG7, and LC3, directly deacetylating them, which is essential for its catalytic activity in autophagy [58]. In particular, LC3 shuttles between the nucleus and the cytoplasm, and during nutrient starvation, it is selectively deacetylated by SIRT1. The deacetylated LC3 interacts with Diabetes and Obesity Regulator (DOR; also known as TP53INP2) and translocates to the cytoplasm, where it interacts with ATG7 to promote autophagy [59].

### Regulation of acetylation

Acetylation, as a modification, is often regulated through enzymatic reactions, where KATs catalyze the transfer of an acetyl group from acetyl-CoA to the ε-amino side chain of lysine, resulting in acetylation, while KDACs can reverse this process. Together, they regulate acetylation modifications. Additionally, non-enzymatic regulatory mechanisms also play a significant role in acetylation and deacetylation.

#### Enzyme regulation mechanism

##### Regulation of KATs and KDACs

KATs

The exact number of true KATs in the human proteome remains unclear. Among the reported KATs, there are 13 classic KATs, most of which are classified into three families: GCN5, p300, and MYST19 (Table 1). The remaining KATs, such as alpha-tubulin N-acetyltransferase 1 (TAT1; also known as ATAT1), ESCO1, ESCO2, and HAT1 (KAT1), are relatively dissimilar to one another. Apart from TAT1, all typical KATs mainly localize to the nucleus and are capable of acetylating both histones and non-histone proteins. The substrate specificity of KATs is considered to be determined by their specific subcellular localization, interacting proteins, and the accessibility of lysine residues in substrate proteins. Many KATs have non-overlapping substrates; however, some closely related KATs can acetylate the same sites and exhibit functional redundancy (see Table 1).

**Table 1:** Classification of acetyltransferases.

	Classification		Subcellular localization
**Acetyltransferase family**	**GCN5**	GCN5 (KAT2A)	Nucleus
		PCAF (KAT2B)	Nucleus
	**p300**	CBP (KAT3A)	Nucleus
		p300 (KAT3B)	Nucleus
	**MYST**	TIP60 (KAT5)	Nucleus
		MOZ (KAT6A)	Nucleus
		MORF (KAT6B)	Nucleus
		HBO1 (KAT7)	Nucleus
		MOF (KAT8)	Nucleus
	**Other**	TAT1	Cytoplasm
		ESCO1	Nucleus
		ESCO2	Nucleus
		HAT1 (KAT1)	Nucleus

*KAT* lysine acetyltransferase, *GCN5* general control nonderepressible 5, *PCAF* p300/CBP-associated factor, *CBP* CREB-binding protein, *p300* E1A-binding protein p300, *TIP60* Tat-interacting protein of 60 kDa, *MOZ* monocytic leukemia zinc finger protein, *MORF* MOZ-related factor, *HBO1* histone acetyltransferase binding to ORC1, *MOF* males absent on the first, *TAT1* tiny acetyltransferase 1, *ESCO1/2* establishment of cohesion 1/2 homolog, *HAT1* Histone acetyltransferase 1.

KDACs

The human genome encodes 18 KDACs, which can be divided into two major classes: Zn^2+^-dependent HDACs and NAD^+^-dependent Sirtuin deacetylases (Table 2). Zn^2+^-dependent HDACs share a highly conserved deacetylase domain, commonly referred to as classical HDACs or classical KDACs. Based on their phylogenetic conservation and sequence similarity, classical KDACs are further divided into four categories: Class I, Class IIa, Class IIb, and Class IV [60]. Class I and Class IV KDACs are nuclear, Class IIb KDACs are cytoplasmic, and Class IIa signaling-responsive KDACs are primarily nuclear but are exported to the cytoplasm upon signal activation. Sirtuin deacetylases, also known as class III KDACs, are localized in various cellular compartments, including the nucleus (SIRT1 and SIRT6), nucleolus (SIRT7), cytoplasm (SIRT2), and mitochondria (SIRT3, SIRT4, and SIRT5) [61].

**Table 2:** Classification of deacetylases.

	Classification			Subcellular localization
**Deacetylase**	**Classical deacetylases (Zn**^**2+**^**-dependent)**	I	HDAC1	Nucleus
			HDAC2	Nucleus
			HDAC3	Nucleus
			HDAC8	Nucleus
		IIa	HDAC4	Nucleus
			HDAC5	Nucleus
			HDAC7	Nucleus
			HDAC9	Nucleus
		IIb	HDAC6	Cytoplasm
			HDAC10	Cytoplasm
		IV	HDAC11	Nucleus
	**Sirtuin deacetylases (NAD**^**+**^**-dependent)**	III	SIRT1	Nucleus
			SIRT2	Cytoplasm
			SIRT3	Mitochondria
			SIRT4	Mitochondria
			SIRT5	Mitochondria
			SIRT6	Nucleus
			SIRT7	Nucleolus
	**Other**	TCF1		Nucleus
		LEF1		Nucleus

*HDAC* histone deacetylase, *HDAC1* histone deacetylase 1, *HDAC2* histone deacetylase 2, *HDAC3* histone deacetylase 3, *HDAC8* histone deacetylase 8, *HDAC4* histone deacetylase 4, *HDAC5* histone deacetylase 5, *HDAC7* histone deacetylase 7, *HDAC9* histone deacetylase 9, *HDAC6* histone deacetylase 6, *HDAC10* histone deacetylase 10, *HDAC11* histone deacetylase 11, *SIRT* sirtuin, *SIRT1* sirtuin 1, *SIRT2* sirtuin 2, *SIRT3* sirtuin 3, *SIRT4* sirtuin 4, *SIRT5* sirtuin 5, *SIRT6* sirtuin 6, *SIRT7* sirtuin 7, *TCF1* T-cell factor 1, *LEF1* lymphoid enhancer-binding factor 1.

It is noteworthy that nearly half of the deacetylase activities are weak or absent, or target other types of acylation reactions. For example, SIRT5, as a sirtuin-type desuccinylase, catalyzes the demalonylation, desuccinylation, and deglutarylation of mitochondrial enzymes associated with various metabolic pathways [62, 63]. SIRT4 removes the acyl groups from methylglutaryl lysine, hydroxymethylglutaryl lysine, and 3-methylglutaryl lysine [64]. SIRT6 exerts defatty-acylase activity and mono-ADP-ribosylation [65]. KDACs of Class IIa lack significant catalytic activity due to variations in conserved amino acids within the catalytic pocket [60] (see Table 2).

Regulation of acetyl-CoA synthesis

Acetyl-CoA is an important metabolic product that plays a crucial role in energy generation in the mitochondria and in lipid biosynthesis in the cytoplasm. Due to its difficulty in penetrating the mitochondrial membrane, the pools of Acetyl-CoA in mitochondria and non-mitochondrial compartments are independently produced. However, Acetyl-CoA can freely diffuse between the cytoplasm and the nucleus through nuclear pores. Acetyl-CoA in mitochondria is produced by the pyruvate dehydrogenase complex, β-oxidation of fatty acids, or amino acid metabolism. In non-mitochondrial pools, Acetyl-CoA is generated in the cytoplasm and nucleus by ATP citrate lyase (ACLY), acetyl-CoA synthetase short-chain family member 2 (ACSS2), and the pyruvate dehydrogenase complex (PDC) [1].

Acetylation is directly related to the levels of acetyl-CoA, which is produced in a cell-compartment-specific manner and can locally drive acetylation. For instance, nuclear ACLY, ACSS2, and PDC have been reported to regulate histone acetylation and gene transcription by generating acetyl-CoA in specific locations [66]. In yeast, the depletion of mitochondrial acetyl-CoA only eliminates the acetylation of mitochondrial proteins without affecting the acetylation of nuclear proteins [67]. The fluctuations in acetyl-CoA levels manipulated by genetics and diet are associated with changes in acetylation levels, further indicating that acetyl-CoA is a rate-limiting factor for many acetylation events [68].

#### Non-enzyme regulation

##### Cellular metabolism regulation through acetylation

**Regulation of acetylation through the perception of energy metabolism status and acetyl-CoA concentration**.

Studies have shown that activated sirtuins transmit metabolic signals to a variety of proteins, including histones, via deacetylation, thereby mediating adaptive transcriptional reprogramming. The NAD^+^-dependent enzymatic activity of SIRT2 enables it to sense the cell’s energy status. Under nutrient-restricted conditions (such as glucose deprivation), cellular energy status shifts, which may activate SIRT2 and other deacetylases by altering the NAD^+^/NADH ratio or local availability rather than absolute levels. This deacetylation conveys metabolic signals to diverse proteins, including histones [69]. Metabolism can influence protein acetylation by altering the cellular concentrations of NAD^+^ and acetyl-CoA. On one hand, during fasting, the relative concentration of NAD^+^ increases, leading to enhanced enzymatic activity of Sirtuins and their targets’ deacetylation [70]. On the other hand, the activity of acetyltransferase varies with the concentration of acetyl-CoA. When nutrient abundance increases, the cellular concentration of acetyl-CoA rises, leading to an increase in acetyltransferase activity and the acetylation of target proteins [1].

**pH-mediated regulation of acetylation**

The ε-amino group of lysine is protonated at acidic and neutral pH. Since lysine must be deprotonated to allow for acetylation, the rate of acetylation is influenced by the protonation state of lysine. In the enzyme acetylation reaction, lysine is deprotonated by the active site residues of KATs. However, under basic pH, lysine naturally deprotonates, and the deprotonated lysine, acting as a nucleophile, approaches the electrophilic carbonyl center of acetyl-CoA. Therefore, the increase in alkaline pH raises the proportion of deprotonated lysine, leading to an increase in non-enzymatic acetylation [71]. In fact, non-enzymatic acetylation preferentially occurs on lysine residues flanked by positively charged amino acids [72] and may be favored by the higher pH environment within the mitochondrial matrix [73].

**Non-enzymatic regulation of acetylation through other acylation reactions**.

Lysine can be acylated by an increasing number of acyl-Coenzyme A (Acyl-CoA) species, and the non-enzymatic mechanisms appear to be associated with most Acyl-CoAs [74]. Interestingly, some acyl-CoAs, such as succinyl-CoA, glutaryl-CoA, and hydroxymethylglutaryl-CoA, exhibit significantly higher reactivity compared to acetyl-CoA [62, 75, 76]. This is because the carboxylate groups of these acyl-CoA molecules can undergo endogenous nucleophilic attack on the thioester bond of coenzyme A, resulting in the formation of a cyclic anhydride that is more reactive than the parent acyl-CoA, thereby facilitating a more effective non-enzymatic modification of proteins.

##### Time regulation of acetylation

**Cell cycle - histone acetylation kinetics**: Imaging and proteomic analysis of chromatin-associated proteins indicate that most KATs are expelled from the chromatin during mitosis [77]. The quantitative mass spectrometry-based method detected a reduction of more than twofold in acetylation at multiple H3 and H4 sites during mitosis [78, 79]. Another type of cell cycle transition is DNA replication, which can potentially disrupt histone acetylation modifications. DNA replication begins at thousands of genomic locations known as “replication origins,” and the positioning and activation of these origins are closely related to the acetylation of H3 and H4, as well as increased chromatin accessibility [80]. This indicates that the periodic metabolic changes and physicochemical properties within cells also affect the level of acetylation.

##### Acetylation spatial regulation

An alternative strategy for controlling enzyme selectivity and activity is through spatial confinement, which enables acetylation to occur preferentially on proteins within specific compartments such as the nucleus, cytoplasm, mitochondria, and endoplasmic reticulum. Some KATs are capable of responding to intracellular signals that alter their subcellular distribution. For instance, KAT3B typically maintains a reservoir in both the nucleus and cytoplasm, but in cases of starvation, its localization is predominantly within the nucleus [81]. The treatment that causes DNA double-strand breaks can induce the relocation of SIRT3 from the nucleus to the mitochondria [82]. Furthermore, calcium/calmodulin-dependent protein kinase-mediated phosphorylation of HDAC5 triggers its export from the nucleus to the cytoplasm during myogenic differentiation [83].

## Overview of apoptosis

Apoptosis is a genetically controlled, autonomous, and orderly form of programmed cell death that maintains internal environmental stability. It plays a critical role in development, maintenance of tissue homeostasis, immune regulation, and the elimination of abnormal cells in the body [84]. In the 1970s, Kerr, Wylie, and Currie defined the ultrastructural characteristics of different types of cell death through electron microscopy, including cytoplasmic shrinkage, nuclear condensation, and the preservation of membrane and organelle integrity—this process is referred to as “apoptosis” [85]. In general, apoptosis is divided into extrinsic and intrinsic pathways.

### Core regulation of apoptosis: caspases

Caspases are a class of cysteine proteases that are found in various types of cells in an inactive proenzyme form. In mammals, they are classified based on their roles in apoptotic regulation into initiator caspases, such as caspase-1, -2, -4, -5, -8, -9, -10, -11, and -12; and executioner caspases, such as caspase-3, -6, -7, and -14. When external factors lead to the deficiency of growth factors, steroid hormones, or the binding of death receptors, or when intrinsic factors cause DNA damage, caspases are activated. Initiator caspases possess a longer N-terminal pro-domain structure that enables them to form a protein platform that regulates caspase activation, such as the interaction between the pro-domain of caspase-9 and Apoptotic Protease-Activating Factor 1 (APAF-1) along with cytochrome c (Cyt c), which initiates apoptotic bodies [86]. Specific executioner caspases, such as caspase-3, -6, -7, and -14, cleave proteins that maintain nuclear structure. These include Lamin (a skeletal protein constituting the nuclear lamina, whose cleavage directly leads to nuclear envelope disintegration and nuclear fragmentation, representing a classic morphological feature of apoptosis), Poly(ADP-ribose) polymerase (PARP) (a key enzyme in DNA damage repair; caspase-mediated cleavage inactivates PARP, thereby terminating cellular DNA repair functions and ensuring the irreversibility of the apoptotic program), DNA-dependent protein kinase (DNA-PK) (similar to PARP, DNA-PK is another critical protein involved in DNA double-strand break repair; it is cleaved and inactivated by caspase-3, further compromising the cell’s repair capacity), and Inhibitor of caspase-activated deoxyribonuclease (ICAD) (ICAD acts as an inhibitor and molecular chaperone for the CAD enzyme, serving as a key regulatory point for DNA fragmentation during apoptosis; cleavage of ICAD by caspase-3 triggers its dissociation from CAD, thereby releasing and activating the CAD enzyme, which subsequently enters the nucleus and degrades DNA, generating the characteristic apoptotic DNA ladder). Concurrently, executioner caspases cleave cytoskeletal proteins such as Fodrin, an essential component of the cytoplasmic skeleton; cleavage of Fodrin by caspases directly results in the collapse of the cytoskeleton, inducing membrane blebbing and ultimately leading to the formation of apoptotic bodies. Cell death is ultimately induced through the aforementioned pathways [87].

### The occurrence of apoptosis

The release of Cyt c from mitochondria forms apoptotic bodies, which are a hallmark of apoptosis. The alteration of mitochondrial outer membrane permeability (MOMP) is crucial for determining whether Cyt c can enter the cytosol. MOMP is regulated by members of the B-cell lymphoma 2 (Bcl-2) family of proteins, which include both anti-apoptotic and pro-apoptotic proteins. The interaction between these two classes of proteins determines whether a cell will undergo apoptosis [88]. BAX–BAK is a pro-apoptotic protein belonging to the Bcl-2 family and is a key player in the intrinsic (mitochondrial) apoptotic pathway. It is activated with the help of BH3-only proteins and forms pores in the outer mitochondrial membrane, leading to an increase in mitochondrial outer membrane permeabilization (MOMP). This process results in the release of cytochrome c, forming apoptosomes that induce apoptosis [89]. Moreover, the imbalance of calcium ion homeostasis also plays a role in apoptosis. When Ca^2+^ is present at high concentrations in the cytoplasm, a high Ca^2+^ microdomain can form near the endoplasmic reticulum (ER), which promotes the uptake of Ca^2+^ by the voltage-dependent anion channel (VDAC) located on the outer mitochondrial membrane (OMM) [90]. Ca^2+^ then enters the mitochondrial matrix through the mitochondrial calcium uniporter (MCU) via the inner mitochondrial membrane (IMM). Normal levels of Ca^2+^ in the matrix play various physiological roles, such as the interaction of Ca^2+^ with cyclophilin D and ANT to form the mitochondrial permeability transition pore (mPTP). However, sustained high levels of calcium overload lead to the opening of the mPTP, mitochondrial swelling, and rupture of the outer mitochondrial membrane (OMM). The rupture of the OMM is associated with the release of pro-apoptotic factors like Cyt c and apoptosis-inducing factor (AIF), which subsequently trigger apoptosis [91]. At the same time, calcium overload promotes the decomposition of complex II in the respiratory chain by binding to cardiolipin in the inner mitochondrial membrane (IMM), leading to the release of various subunits, resulting in the production of large amounts of reactive oxygen species (ROS) and ultimately causing cell apoptosis [92].

**Fig. 1:**
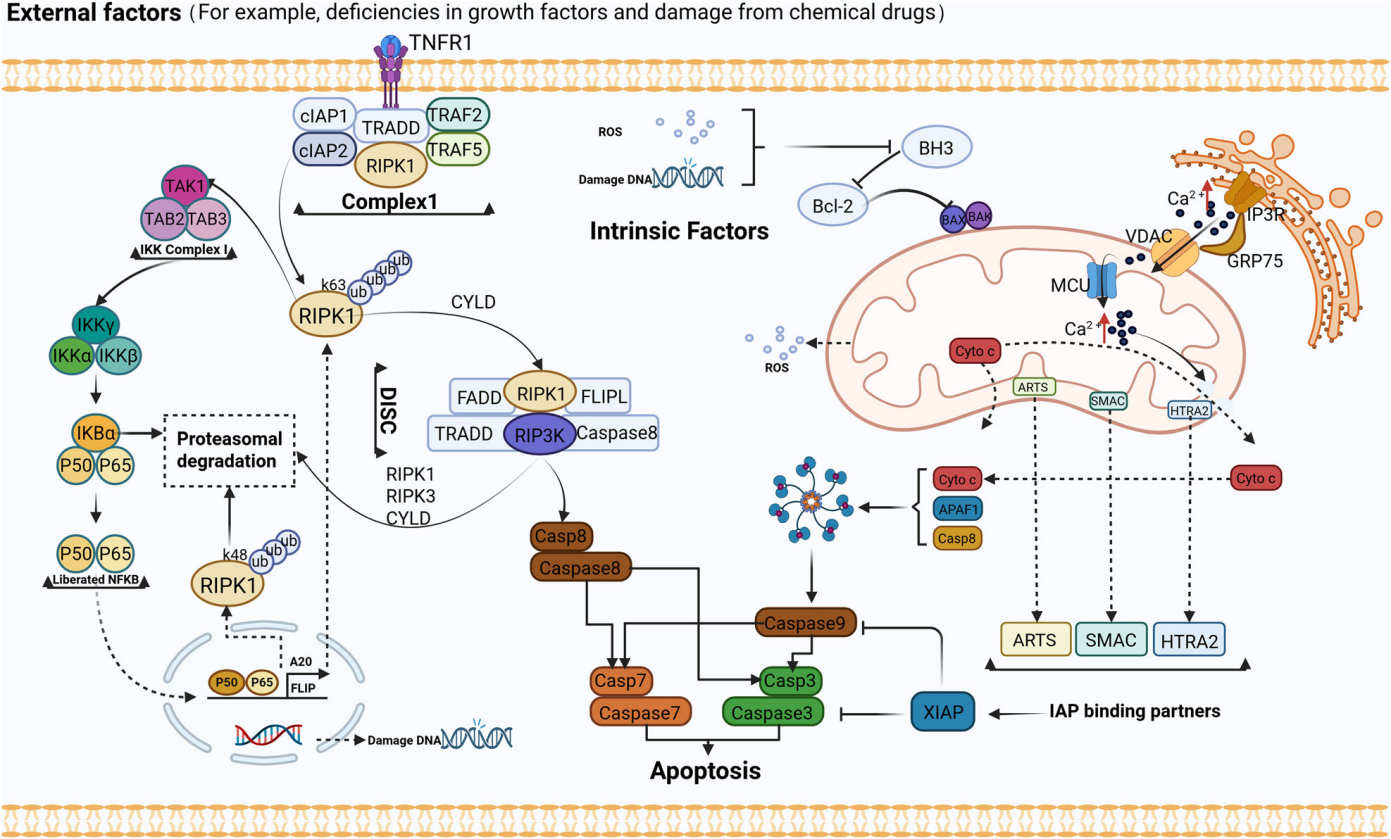
Diagram of apoptosis regulatory mechanisms. Activation of the extrinsic apoptosis pathway depends on TNFR1-mediated transmission of stimulatory signals such as TNF. Upon binding of TNF to its receptor, RIPK1, TRADD, cIAP1, cIAP2, TRAF2, and TRAF5 are recruited to TNFR1 to form Complex I. cIAP1 and cIAP2 mediate Lys63-linked ubiquitination of RIPK1, which anchors TAK1 and its partners TAB2 and TAB3. The signal is then relayed to IKK (IκBα kinase), which degrades IκBα, the inhibitor of canonical NF-κB in the cytoplasm. Once released from IκBα-mediated inhibition, NF-κB translocates to the nucleus and drives the transcription of pro-survival genes and feedback antagonists. Among the pro-survival NF-κB target genes, A20 and FLIP promote the assembly of a DISC by facilitating interactions among RIPK1 and cytosolic RIPK3, TRADD, FADD, caspase-8, and FLIP. FLIPL can heterodimerize with caspase-8 and promote the cleavage and degradation of CYLD, RIPK1, and RIPK3. However, the DISC can also promote caspase-8 homodimerization and catalytic activation, thereby activating caspase-3 and caspase-7 and triggering apoptosis. The intrinsic apoptotic pathway is primarily triggered by DNA damage and ROS, which leads to the activation of BAX–BAK and results in mitochondrial MOMP, releasing cytochrome c to form apoptotic bodies and induce apoptosis. On the other hand, cytoplasmic Ca^2+^ overload can transport Ca^2+^ into the mitochondria through the VDAC protein in the outer mitochondrial membrane and the MCU protein in the inner mitochondrial membrane. When the Ca^2+^ concentration inside the mitochondria becomes excessive, it can lead to the opening of the mPTP, causing the release of pro-apoptotic factors such as cytochrome c and AIF, thereby triggering apoptosis. IAPs suppress the occurrence of apoptosis, but their inactivation induced by SMAC, HTRA2, and ARTS initiates apoptosis. TNFR1 tumor pyroptosis factor receptor 1, RIPK1 receptor-interacting protein kinase 1, TRADD NF receptor type 1-associated death domain protein, cIAP1 cellular inhibitor of apoptosis protein 1, cIAP2 cellular inhibitor of apoptosis protein 2, TRAF2 TNF receptor‑associated factor 2, TRAF5 TNF receptor‑associated factor 5, RIPK3 receptor‑interacting serine/threonine‑protein kinase 3, TAK1 transforming growth factor-β-activated kinase 1, TAB2 TAK1-associated binding protein 2, TAB3 TAK1-associated binding protein 3, IκBα inhibitor of κ (kappa) B α (alpha), P50 nuclear factor kappa-light-chain-enhancer of activated B cells 1, P65 RELA proto-oncogene, NF-KB Subunit, A20 TNF alpha induced protein 3, NFκB nuclear factor kappa-light-chain-enhancer of activated B Cells, FADD Fas-associated protein with death domain, FLIP Fas-associated death domain-containing protein-associated inhibitor, DISC death-inducing signaling complex, MOMP mitochondrial outer‑membrane permeabilization, Cytc cytochrome c, VDAC voltage‑dependent anion channel, MCU mitochondrial calcium uniporter, mPTP mitochondrial permeability transition pore, AIF apoptosis‑inducing factor, IAPs inhibitor of apoptosis proteins, SMAC second mitochondria‑derived activator of caspase, HTRA2 high temperature requirement protein A2, ARTS apoptosis‑related protein in TGF‑β signaling pathway.

The extrinsic activation pathway is triggered by the binding of death receptors, such as when tumor pyroptosis factor (TNF) binds to tumor pyroptosis factor receptor 1 (TNFR1). This binding recruits receptor-interacting protein kinase 1 (RIPK1), TNF receptor type 1-associated death domain protein (TRADD), cellular inhibitor of apoptosis protein 1 (cIAP1), and cellular inhibitor of apoptosis protein 2 (cIAP2), as well as TNF receptor-associated factor 2 (TRAF2) and TNF receptor-associated factor 5 (TRAF5). Together, these proteins form a complex referred to as complex I [93] (Fig. 1). cIAPs mediate the Lys63-linked ubiquitination of RIPK1, while the deubiquitinating enzyme cylindromatosis (CYLD) removes the Lys63-linked ubiquitin chains from RIPK1, leading to its dissociation from complex I [94]. Subsequently, RIPK1 forms a death-inducing signaling complex (DISC), which includes RIPK3, TRADD, Fas-associated protein with death domain (FADD), caspase-8, and Fas-associated death domain-containing protein-associated inhibitor (FLIP), also known as complex IIa. On one hand, DISC can promote the cleavage and degradation of CYLD, RIPK1, and RIPK3 to enhance cell survival; on the other hand, it can facilitate the homodimerization and enzymatic activation of caspase-8, subsequently triggering apoptosis. Furthermore, RIPK1 can form a lethal platform known as complex IIb or ripoptosome with FADD and caspase-8, which is negatively regulated by cIAP1, cIAP2, and X-Linked Inhibitor of Apoptosis Protein (XIAP), and can also activate caspase-8-mediated apoptosis.

### Apoptosis inhibition

Inhibitor of Apoptosis Proteins (IAPs) are a class of proteins that play critical roles within cells, primarily by inhibiting apoptosis through various mechanisms. IAPs contain BIR (Baculoviral IAP Repeat) domains, allowing them to bind to Caspases and inhibit their activity in vitro. Some IAPs also possess RING (Really Interesting New Gene) domains, enabling them to function as E3 ubiquitin ligases, thereby ubiquitinating key cell death proteins. The ubiquitination of Caspases can suppress apoptosis [95].

When a cell is about to initiate apoptosis, IAPs must be inactivated by endogenous antagonists. The most notable IAP-binding proteins in mammals are the Second Mitochondria-derived Activator of Apoptosis (SMAC) and High Temperature Requirement A2 (HTRA2). SMAC and HTRA2 are localized in the intermembrane space of mitochondria and are transported to the cytosol following mitochondrial outer membrane permeabilization (MOMP). Once released into the cytosol, these proteins promote the activation of caspases by binding to IAPs, thereby triggering apoptosis [96]. Another mammalian IAP antagonist is ARTS, which does not contain IBM but is similar to RHG proteins and is localized to the outer mitochondrial membrane [97]. It acts upstream of MOMP and the release of mitochondrial proteins. XIAP also possesses E3 ligase activity and is considered one of the most potent inhibitors of caspases in vitro [98].

## Overview of ferroptosis

Ferroptosis is an iron-dependent, non-apoptotic form of cell death characterized by the accumulation of intracellular lipid peroxides, leading to membrane rupture and cell death. It is defined by the interaction of free intracellular iron or iron-containing enzymes with oxygen and polyunsaturated fatty acids (PUFAs), resulting in high levels of membrane lipid peroxides [99].

### Execution of ferroptosis

Regarding the execution of ferroptosis, the primary focus is on how the accumulation of lipid peroxides leads to the rupture of the cell membrane. The Na⁺/K⁺-ATPase is a crucial membrane protein that belongs to the P-type ATPase family, typically composed of multiple subunits, mainly including the α subunit and the β subunit. The α subunit is responsible for ion transport, while the β subunit plays a supportive role in the enzyme’s stability and function. This enzyme hydrolyzes ATP to provide energy, actively transporting sodium and potassium ions. For each molecule of enzyme that hydrolyzes one molecule of ATP, it usually translocates three Na⁺ ions from the intracellular space to the extracellular space, while moving two K⁺ ions from the extracellular space into the cell. Piezo1 is a non-selective cation channel formed by multiple transmembrane structures, mediating the passage of ions such as Na^+^ and Ca^2+^. It is primarily located in the cell membrane and is capable of sensing and responding to changes in mechanical forces such as pressure, tension, and shear stress [100]. TRP channels are a class of non-selective cation channels typically composed of six transmembrane helical structures that form a central pore, allowing ions such as Na^+^, Ca^2+^, and Mg^2+^ to pass through. They play a crucial role in the cellular response to mechanical stimuli, participating in mechanosensation and signal transduction [101].

**Fig. 2:**
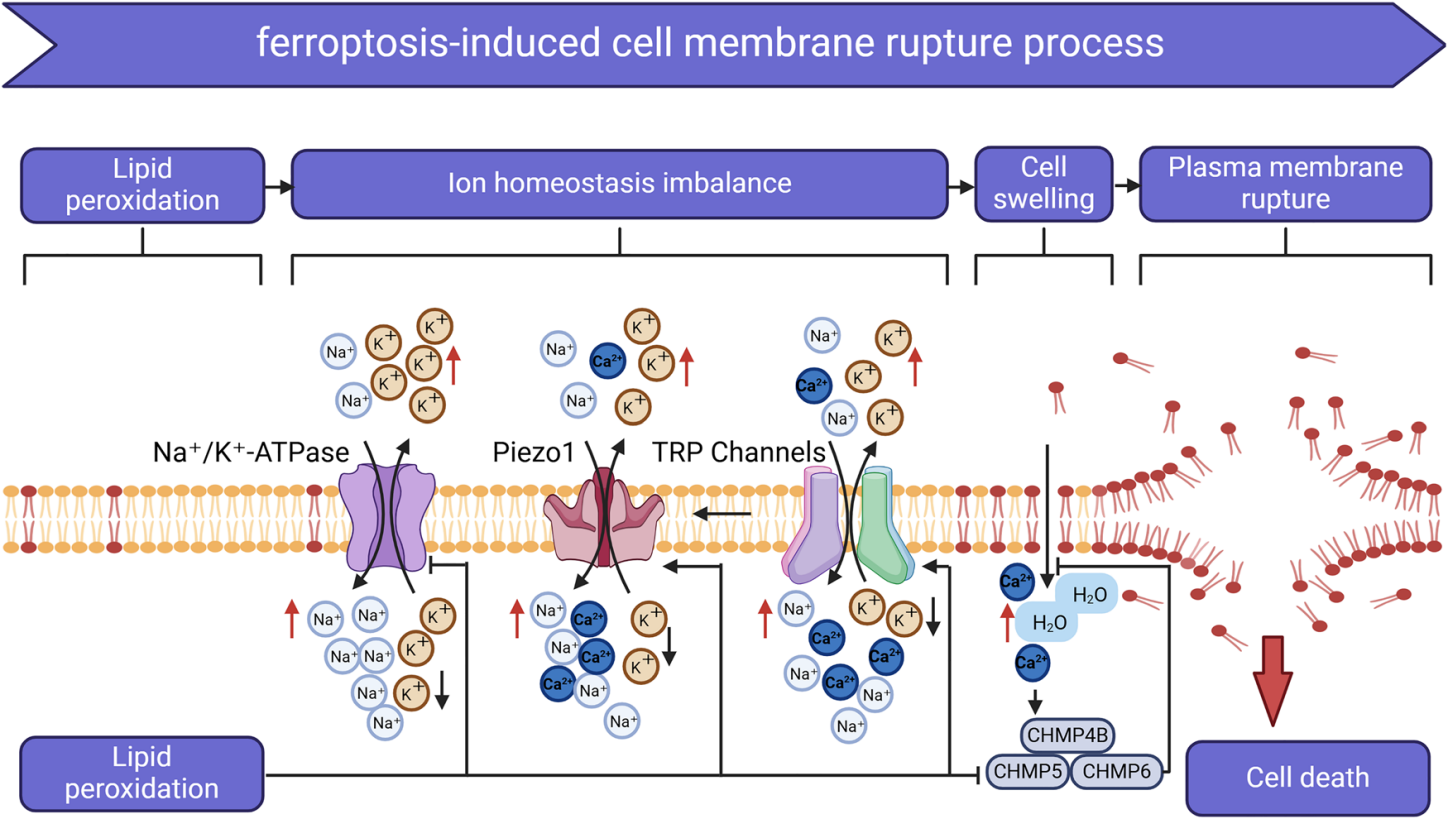
Mechanisms of cell membrane rupture induced by ferroptosis. Lipid peroxidation leads to the inactivation of Na⁺/K⁺-ATPase, resulting in elevated intracellular Na⁺ levels. This osmotic effect causes cellular edema and increased tension, which activates Piezo1 and TRP channels, leading to the influx of Na⁺ and Ca²⁺ and the efflux of K⁺, further exacerbating cellular swelling and membrane tension. Additionally, lipid peroxidation restricts the recruitment of CHMP5 and CHMP6 to the plasma membrane, which are involved in the local repair processes of the cell membrane, ultimately resulting in cell rupture and death. Piezo1 mechanosensitive ion channel piezo1, TRP transient receptor potential channel, CHMP5 charged multivesicular body protein 5, CHMP6 charged multivesicular body protein 6, CHMP4B charged multivesicular body protein 4B.

Research has found that excessive accumulation of lipid peroxidation products in the cell membrane can lead to the inactivation of Na⁺/K⁺-ATPase, resulting in a high intracellular Na⁺ state and exacerbating the cytoplasmic univalent cation gradient [102]. Concurrently, increased membrane tension activates Piezo1 and TRP channels, leading to the influx of Na⁺ and Ca²⁺ and the loss of K⁺. These alterations cause elevated intracellular levels of Na⁺ and Ca²⁺, disrupt ionic homeostasis, and result in cell swelling [103]. Increased intracellular Ca^2+^ can recruit Charged Multivesicular Body Protein 5 (CHMP5) and Charged Multivesicular Body Protein 6 (CHMP6) to the plasma membrane to participate in the localized repair of the cell membrane; however, high levels of lipid peroxidation can inhibit the membrane repair functions mediated by CHMP4B, CHMP5, and CHMP6, ultimately leading to the rupture of the cell membrane [104] (Fig. 2).

### Regulation of ferroptosis

#### Regulation of ferroptosis mediated by intervention in PUFAs levels

PUFAs refer to polyunsaturated fatty acids that contain two or more double bonds within their molecular structure. This double bond arrangement is prone to oxidation, leading to the formation of lipid peroxides; consequently, PUFAs play a crucial role in the process of ferroptosis. More specifically, the threshold for cellular ferroptosis can be determined by lipid composition, with the relative levels of different lipids dictating whether a cell is in a ferroptosis-sensitive or ferroptosis-resistant state. For example, reducing the ratio of more easily oxidizable PUFAs to less oxidizable monounsaturated fatty acids (MUFAs) is sufficient to shift cells from being sensitive to ferroptosis to being resistant to ferroptosis [105].

Additionally, during the process of lipid peroxidation, when lipid hydroperoxide interacts with ferrous ions (Fe^2+^), it generates peroxy radicals. These radicals can extract hydrogen from adjacent acyl chains within the lipid membrane environment, thereby propagating the lipid peroxidation process. Hydrogen abstraction preferentially occurs at PUFAs because, compared with other lipid species, their bis-allylic hydrogens have relatively low bond dissociation energies. This results in a higher rate of lipid peroxide formation in PUFAs than in more saturated lipid types [106].

##### The regulatory mechanism mediated by the endoplasmic reticulum affecting levels of polyunsaturated fatty acids

The endoplasmic reticulum is the primary center for lipid metabolism within the cell, responsible for lipid desaturation, phospholipid synthesis, remodeling, and the biosynthesis of lipid droplets. Therefore, the lipid metabolic enzymes residing in the endoplasmic reticulum play a crucial role in regulating sensitivity to ferroptosis.

Fatty Acid Desaturase 1 (FADS1) and Fatty Acid Desaturase 2 (FADS2) play critical roles in fatty acid metabolism, particularly in the synthesis of PUFAs. They introduce carbon-carbon double bonds into fatty acids, enhancing sensitivity to ferroptosis through the synthesis of specific PUFAs [107]. However, studies have also shown that FADS2 can synthesize MUFAs via non-classical pathways, inhibiting sensitivity to ferroptosis [108].

Long-chain acyl-CoA synthetase (ACSL) is a key enzyme that activates long-chain fatty acids by catalyzing their conjugation with coenzyme A to form acyl-CoA, a critical step in fatty acid metabolism. It activates PUFAs and MUFAs into PUFAs-CoA and MUFAs-CoA, respectively, which are then incorporated into membrane phospholipids, triacylglycerols, or other lipids. The ACSL family includes various isoforms (such as ACSL1, ACSL3, ACSL4, etc.), with different isoforms being expressed in distinct tissues and cell types, each serving specific physiological functions. For instance, the metabolism of PUFAs driven by ACSL4 increases sensitivity to ferroptosis [109], while the ACSL3-dependent metabolism of MUFAs promotes resistance to ferroptosis [110].

Lipid remodeling, specifically the Lands cycle, involves the action of phospholipase cutting one acyl chain from a phospholipid, followed by the re-acylation process where lysolipids interact with acyl-CoA to reform phospholipids. Calcium-independent phospholipase A2 (also known as PLA2G6, PNPLA9, or iPLA2β) can remove oxidized PUFA acyl chains from phospholipids, thereby directly limiting the spread of lipid peroxidation and the occurrence of ferroptosis [111–113]. Lysophosphatidylcholine Acyltransferase 3 (LPCAT3; also known as MBOAT5), 1-Acylglycerol-3-phosphate O-acyltransferase 3 (AGPAT3), and MBOAT7 are localized in the endoplasmic reticulum and are responsible for the re-acylation of lysolipids with PUFAs, contributing to pathways that increase sensitivity to ferroptosis [114–116]. MBOAT1 and MBOAT2, residing in the endoplasmic reticulum, facilitate the re-acylation of MUFAs with lysolipids, providing a pathway to enhance resistance to ferroptosis [117, 118]. Within these two pathways, there may be competition among phospholipid acyltransferases for the re-acylation of the same substrate. The relative levels of different enzymes and substrates will influence the overall composition of the membrane, thereby affecting the cell’s sensitivity to ferroptosis.

In addition to lipid synthesis, the endoplasmic reticulum is also a crucial site for processing and regulating cellular lipids and the redox environment through important transcription factors. Sterol Regulatory Element-Binding Proteins (SREBPs) are transcription factors anchored in the endoplasmic reticulum by two transmembrane domains. They are regulated by the PI3K-AKT-mTORC1 pathway, which mediates their translocation to the Golgi apparatus, where they are cleaved to release active transcription factors. These transcription factors subsequently activate the expression of Stearoyl-CoA Desaturase 1 (SCD1), which catalyzes the synthesis of long-chain monounsaturated fatty acids, such as oleic acid, thereby reducing the membrane’s susceptibility to oxidation and sensitivity to ferroptosis [119, 120].

##### Regulatory mechanisms mediated by other organelles affecting the levels of polyunsaturated fatty acids

**Lipid droplet**: Lipid droplets are intracellular lipid storage particles composed of a neutral lipid core, primarily consisting of triglycerides and sterol esters. The outer layer features a phospholipid monolayer membrane that contains integral and peripheral regulatory proteins, playing a context-specific role in the regulation of ferroptosis [121]. In general, in certain cells, PUFAs are preferentially sequestered in lipid droplets, away from membrane phospholipids, which limits sensitivity to ferroptosis. For example, in glioblastoma cells, if PUFAs are not sequestered in lipid droplets away from membrane phospholipids, sensitivity to ferroptosis can be promoted [122]. Similarly, cancer cells growing under acidic conditions that simulate the tumor microenvironment accumulate lipid droplets. Inhibiting the synthesis of these lipid droplets causes PUFAs to shift from triacylglycerols (TAG) to phospholipids, making the cells more sensitive to ferroptosis [123].

However, lipid droplets do not always prevent ferroptosis. In clear cell renal cell carcinoma cells, hypoxia-inducible factor 2 alpha (HIF-2α) regulates the expression of hypoxia-inducible lipid droplet-associated protein (HILPDA). When HILPDA expression increases, it leads to an increase in the number of lipid droplets and the levels of PUFA-TAG, which is similarly associated with increased sensitivity to ferroptosis [124]. Furthermore, in certain cases, the formation of lipid droplets does not impact ferroptosis. For instance, in fibroblast sarcoma cells incubated with extracellular MUFAs, the increase in the number of lipid droplets and the disruption of lipid droplet synthesis do not alter sensitivity to ferroptosis; meanwhile, treatment with exogenous MUFAs reduces the sensitivity of membrane lipids to ferroptosis, indicating that in some instances, lipid droplets do not participate in the regulation of ferroptosis [125].

**Peroxisome**: Peroxisomes play a crucial role in multiple metabolic processes, including the breakdown of very long-chain fatty acids and the metabolic decomposition of hydrogen peroxide by catalase. Ether lipids are essential components of cell membranes, participating in membrane structure and function; their presence helps maintain the fluidity and stability of the cell membrane. Peroxisomes can synthesize ether lipids, and ether lipids containing polyunsaturated fatty acids are abundant in the plasma membrane, where these lipids can promote the execution of ferroptosis under certain conditions [116].

#### Intervention in the regulation of ferroptosis mediated by movable iron levels

Lipid peroxidation can be initiated in cells through both enzymatic and non-enzymatic pathways. The enzymatic reactions of lipid peroxidation are primarily represented by those regulated by lipoxygenases, which utilize non-heme iron to exert their activity and directly catalyze the formation of lipid free radicals. However, in several commonly used cell lines for ferroptosis research, the expression of the lipoxygenase (Arachidonate Lipoxygenase, ALOX) gene is nearly undetectable, indicating that the role of ALOX family members in ferroptosis is limited [126]. Non-enzymatic lipid peroxidation is catalyzed by redox-active metals, particularly iron. The labile iron pool refers to the iron within cells that can be rapidly mobilized and utilized, usually existing in the form of ferrous iron (Fe²⁺). This is in contrast to the iron found in heme (the prosthetic group of many proteins, such as hemoglobin), iron-sulfur clusters (the cofactors of many enzymes), and ferritin (a specialized iron storage protein), where iron exists in a complex form. Reactive iron exhibits a high degree of reactivity, particularly in its reaction with hydrogen peroxide (H_2_O_2_). This reaction, known as the Fenton reaction, involves the cycling between ferrous (Fe^2+^) and ferric (Fe^3+^) ions, converting H_2_O_2_ into hydroxide ions (OH–) and hydroxyl (HO•) radicals, or protons and peroxy radicals (HOO•). Both hydroxyl and peroxy radicals can initiate lipid peroxidation. Similar to H2O2, phospholipid hydroperoxides (PL-OOH) can also undergo iron-catalyzed Fenton reactions, producing lipid hydroxyl radicals (PLO•) and lipid peroxyl radicals (PLOO•). If PL-OOH is not quickly neutralized after its formation, it can propagate peroxidation to adjacent polyunsaturated fatty acid phospholipids (PUFA-PLs) in the presence of mobilizable iron. Therefore, regulating the cellular pool of mobilizable iron can influence the process of ferroptosis [127].

##### Regulatory mechanisms mediated by lysosomes affecting levels of mobile iron

Iron can be imported into cells through the transferrin–transferrin receptor (TFRC) system. During this process, the Fe³⁺–transferrin complex is internalized by TFRC and ultimately transported to the acidic environment of the lysosome, where it is released. As such, lysosomal deacidification typically inhibits ferroptosis [128]. Moreover, silencing TFRC, which restricts the intake of iron into cells, can also suppress ferroptosis [129]. Degradation of ferritin within lysosomes is a crucial pathway for the generation of bioavailable iron. The adapter protein Nuclear Receptor Coactivator 4 (NCOA4) links ferritin, which carries iron, to the lysosomal degradation mechanism through a process known as selective ferritin autophagy. Inhibition of NCOA4 can lead to a decrease in bioavailable iron levels, thereby reducing sensitivity to ferroptosis [130]. Nuclear Factor Erythroid 2-Related Factor 2 (NRF2/NFE2L2) plays a crucial role in regulating the labile iron pool. Knockout of NFE2L2/NRF2 can reduce the expression of HERC2 and VAMP8, which respectively lead to the simultaneous increase of ferritin and NCOA4, recruit divalent iron to the autophagosome, and hinder ferritin autophagy, ultimately resulting in the accumulation of divalent iron in the autophagosome, elevated intracellular labile iron levels, and enhanced sensitivity to ferroptosis [131].

#### Regulation of ferroptosis mediated by interventions of reactive oxygen species levels

Reactive Oxygen Species (ROS) refer to a class of highly reactive molecules, mainly including superoxide anions (O₂⁻), hydrogen peroxide (H₂O₂), and hydroxyl radicals (·OH). They primarily originate from the mitochondrial respiratory chain, cytochrome P450 enzymes, and the oxidative burst of inflammatory cells. In the context of ferroptosis, they represent another significant contributor to lipid peroxidation.

##### Regulation of ferroptosis mediated by the antioxidant enzyme system

Glutathione Peroxidase 4 (GPX4) is a selenium-containing enzyme primarily found in the cytoplasm and mitochondria. Its main function is to catalyze the reduction reactions of hydrogen peroxide and lipid peroxides, using glutathione (GSH) as a reducing agent, thereby protecting cells from oxidative damage. Although GPX4 in mitochondria may inhibit ferroptosis under certain circumstances, the cytosolic GPX4 plays a more critical role in preventing ferroptosis [132].

Cysteine is indispensable for combating ferroptosis and serves as both the reduced cofactor of GPX4 and a precursor for GSH synthesis [133]. The Xc-cystine-glutamate reverse transporter is a sodium-dependent binary transporter primarily composed of two subunits: SLC7A11 (also known as xCT) and SLC3A2. Its main function is to transport intracellular glutamate out of the cell while concurrently bringing extracellular cystine into the cell, serving as one of the primary sources of intracellular cysteine. Extracellular proteins rich in cysteine, such as albumin, can be indirectly obtained through endocytosis and subsequent lysosomal degradation [134]. The gamma-glutamyl cycle is another method of cysteine uptake, where gamma-glutamyltransferase 1 (GGT1), located in the plasma membrane, can cleave the gamma-glutamyl bond connecting glutamate to cysteine in glutathione (GSH), resulting in the formation of cysteinylglycine dipeptide. This dipeptide can then be absorbed by the cell membrane and further degraded by dipeptidases, producing cysteine for the synthesis of cytosolic GSH [135]. GSH, as a cofactor of GPX4, is synthesized through a two-step enzymatic process involving Glutamate-Cysteine Ligase (GCL) and Glutathione Synthase (GSS). However, even blocking de novo glutathione (GSH) synthesis or knocking out glutamate-cysteine ligase catalytic subunit (GCLC) may not be sufficient to directly induce ferroptosis [136]. The underlying mechanism is attributed to one or more additional cysteine-derived sulfur-containing metabolites that operate in parallel to reduce lipid peroxides. For instance, in pancreatic ductal adenocarcinoma (PDAC) cells, inhibiting GSH synthesis alone fails to effectively induce ferroptosis. Metabolic flux analysis using ¹³C-labeled cystine revealed that a significant portion of intracellular cystine is channeled into the biosynthesis of coenzyme A (CoA). Furthermore, experimental evidence confirmed that the CoA pathway functions independently of GSH as a parallel mechanism conferring resistance to ferroptosis [137].

Selenium is an important component of GPX4. Research has found that under low selenium conditions, the synthesis of GPX4 is inhibited by ribosomal stalling and collisions, which leads to early termination of GPX4 translation [138]. Selenium intake from the diet can enter cells through the endocytosis of selenium-containing carrier protein selenoprotein P, which is secreted by the liver. This process releases selenium through the lysosomal pathway and is mediated by cell surface receptors such as Low-Density Lipoprotein Receptor-Related Protein 8 (LRP8). Research has shown that both knockout and deletion of the LRP8 gene can trigger ferroptosis [139, 140]. Selenium can be converted from inorganic selenium to volatile selenium compounds through the process of cysteine uptake, which is dependent on the xc-cystine-glutamate reverse transport protein, thereby facilitating selenium absorption and the biosynthesis of selenocysteine [141]. Ultimately, this assists GPX4 in exerting its antioxidant effects. It is important to note that selenocysteine is synthesized directly on a specialized tRNA (tRNA[Ser]Sec) through a multi-step process. However, its unique translation mechanism results in a lower translation efficiency for selenoproteins, and under conditions of insufficient selenium supply, it is prone to early termination [141].

##### Regulation of ferroptosis mediated by lipid peroxidation and antioxidant-free radical capture

Reactive Thioester Acylation (RTAs) are a class of antioxidants capable of effectively capturing and neutralizing lipid peroxyl radicals. These antioxidants play a crucial role in biological systems, particularly in protecting cells from oxidative damage and maintaining the intracellular redox balance.

Coenzyme Q10 (CoQ10) is a crucial component of the oxidative phosphorylation mechanism, synthesized in the mitochondria. It can carry two electrons and exists in three redox states: ubiquinone (fully oxidized), semiquinone, and ubiquinol (fully reduced). Its high lipophilicity allows ubiquinol to function as a free radical scavenger, enhancing resistance to ferroptosis. Alpha-tocopherol is a form of vitamin E and is a classic endogenous RTA that can inhibit ferroptosis, but its efficacy is significantly lower than that of many synthetic RTAs [142]. In addition to the above two categories, tetrahydrobiopterin [143, 144], vitamin K, and vitamin A [145] also serve as RTAs and play a role in enhancing resistance to ferroptosis.

Ferroptosis Suppressor Protein 1 (FSP1/AIFM2) is an NAD(P)H and FAD-dependent oxidoreductase that can reduce CoQ10 [146, 147] and vitamin K [148], terminating phospholipid peroxidation by generating panthenol and the reduced form of vitamin K. Research has found that the specific localization of FSP1 to the plasma membrane and lipid droplets can promote its inhibition of ferroptosis. During the translation of FSP1, its N-terminal methionine is removed, and a 14-carbon fatty acid, myristic acid, is covalently linked to a glycine residue, irreversibly anchoring FSP1 to the plasma membrane. This anchoring is a necessary step for its function in exerting anti-ferroptotic activity [146, 147]. At the same time, the targeting of FSP1 to the plasma membrane is sufficient to inhibit ferroptosis, indicating that FSP1 on the plasma membrane plays an important role in the suppression of ferroptosis [146].

##### Regulation of ferroptosis mediated by NADPH synthesis or regeneration of endogenous antioxidant metabolites

Nicotinamide Adenine Dinucleotide Phosphate (NADPH) is widely present in biological systems, serving as an important coenzyme and a key electron carrier within cells. High levels of NADPH are typically associated with stronger resistance to ferroptosis [149], as NADPH is used for the synthesis or regeneration of endogenous antioxidant metabolites. For example, using NADPH, Glutathione Disulfide Reductase (GSR) reduces oxidized glutathione (GSSG) to GSH; FSP1 [146, 147] reduces oxidized Coenzyme Q and oxidized vitamin K to ubiquinol and reduced vitamin K, respectively; and Dihydrofolate Reductase reduces dihydrobiopterin to tetrahydrobiopterin, among other RTAs, to exert an inhibitory effect on ferroptosis [144]. The membrane-associated Ring-CH type finger 6 (MARCHF6) E3 ubiquitin ligase located in the endoplasmic reticulum contains a unique C-terminal binding region that can directly sense the levels of NADPH in the cytoplasm, thereby increasing the enzyme’s activity and leading to changes in the levels of critical regulators of ferroptosis, such as ACSL4 and p53 [150]. It is noteworthy that the embryonic development of Marchf6−/− animals is impaired; however, maternal dietary supplementation with natural RTA vitamin E can improve this situation. Thus, MARCHF6 may play a protective role against ferroptosis during development. Protein 3 containing the HD domain (HD Domain-Containing Protein 3, HDDC3/MESH1) has been proposed as a NADPH phosphatase, whose activity enhances sensitivity to ferroptosis by lowering NADPH levels [151].

NADPH serves as an electron donor for the NOX enzymes located on the plasma membrane and the redox enzymes cytochrome P450 reductase (POR) and cytochrome b5 reductase 1 (CYB5R1) on the endoplasmic reticulum membrane, facilitating the synthesis of ROS [152, 153]. Therefore, NADPH may also play a role in promoting ferroptosis. Aspartate aminotransferase (GOT1) can also exert anti-ferroptotic effects by influencing NADPH production. In pancreatic ductal adenocarcinoma, GOT1 catalyzes NADPH generation via a noncanonical malate–aspartate shuttle pathway, thereby indirectly suppressing ferroptosis. The core mechanism of its anti-ferroptotic activity is that GOT1 maintains sufficient pools of NADPH and glutathione (GSH) to support GPX4 in clearing lipid peroxides. When GOT1 is inhibited, mitochondrial respiratory dysfunction and energy stress ensue, triggering NCOA4-mediated ferritinophagy and the consequent release of labile iron. Concurrently, antioxidant capacity (NADPH/GSH) declines. Under the combined conditions of iron overload and oxidative stress, cells become highly sensitive to stimuli such as cystine deprivation, GPX4 inhibition, or blockade of GSH synthesis, which markedly enhances lipid peroxidation and induces ferroptosis [154].

##### Regulatory mechanisms mediated by organelles affecting levels of reactive oxygen species

**Mitochondria**: Mitochondria are multifunctional organelles that can regulate the sensitivity to ferroptosis through several different mechanisms. Among these, mitochondrial shrinkage and disorganization of the cristae structure are potential morphological markers of ferroptosis [155]. However, the regulation of ferroptosis by mitochondria appears to be related to the type of cell and the biochemical processes occurring within the cell. In human HT-1080 cells, genetic ablation of mitochondria via Parkin-mediated mitophagy revealed that the ferroptosis inhibitor ferrostatin-1 remained effective, with its potency even slightly enhanced. Electron microscopy indicated a proliferation of endoplasmic reticulum (ER) structures following mitochondrial loss, suggesting that ferrostatins may primarily exert their protective effects by acting on the ER [156]. In contrast, studies in Trypanosoma brucei demonstrated that knockout of tryparedoxin peroxidase (TXNPx) induced iron-dependent cell death accompanied by mitochondria-specific lipid peroxidation [157]. The underlying discrepancy may be attributed to differences in cellular type and metabolic state: tumor cells such as HT-1080 often rely predominantly on glycolysis (the Warburg effect), rendering mitochondrial function secondary; whereas trypanosomes depend critically on oxidative phosphorylation for energy production, making them acutely vulnerable to mitochondrial damage. Furthermore, mitochondria can either promote or inhibit ferroptosis under different circumstances.

In terms of increasing sensitivity to ferroptosis in mitochondria, there are the following mechanisms. The mitochondrial tricarboxylic acid (TCA) cycle promotes ferroptosis, primarily because cysteine serves not only as a substrate for glutathione (GSH) biosynthesis—essential for GPX4 activity—but also modulates glutamine catabolism. Under conditions of cysteine deprivation, the reduction in GSH synthesis impairs GPX4 function, thereby inducing ferroptosis. Concurrently, cysteine deprivation enhances glutamine catabolism. The metabolites derived from glutaminolysis lead to the accumulation of TCA cycle intermediates, such as succinate, fumarate, and malate. These intermediates can directly substitute for glutamine to amplify lipid ROS accumulation and cell death, ultimately exacerbating the ferroptotic effect. [158]. Furthermore, TCA cycle intermediate α-ketoglutarate (α-KG) also promotes the occurrence of ferroptosis. The mechanism involves α-KG enabling the TCA cycle to operate fully, generating substantial amounts of NADH and FADH₂. These reducing equivalents subsequently donate electrons to the electron transport chain (ETC), leading to hyperpolarization of the mitochondrial membrane potential. This state facilitates the massive generation of ROS, which ultimately drives ferroptosis [159]. Mitochondria serve as the central hub for the utilization and metabolism of iron, and increased mitochondrial iron uptake mediated by mitochondrial ferritin 1 (MFRN1) appears to enhance sensitivity to ferroptosis [160]. Depletion of NFS1 cysteine desulfurase in mitochondria reduces the synthesis of iron-sulfur clusters, triggering a feedback response to iron deficiency that increases iron uptake and enhances sensitivity to ferroptosis [161]. When the mitochondrial outer membrane becomes permeabilized throughout the cell, it leads to the activation of the ATF4 pathway and enhances sensitivity to Ferroptosis induced by GPX4 inhibitors [162]. In general, mitochondrial ETC activity inhibits the occurrence of ferroptosis, as the former is essential for the activity of mitochondrial-localized Dihydroorotate Dehydrogenase (DHODH) [163] and Glycerol-3-Phosphate Dehydrogenase (GPD2) [164], both of which contribute to the production of reduced coenzyme Q and suppress ferroptosis. However, in some cancer cells, mitochondrial ETC activity promotes ferroptosis [158].

In certain cases, mitochondrial activity can reduce sensitivity to ferroptosis. Mitochondrial Fusion Protein 1 (MFN1)-mediated mitochondrial fusion decreases sensitivity to RSL3, a covalent and selective inhibitor of GPX4, induced ferroptosis [165]. The activation of Oma1 leads to mitochondrial fragmentation and induces an integrated stress response along the Oma1-Dele1-Atf4 signaling axis, which can inhibit ferroptosis and provide protective effects [166]. Mitochondria are also the site of CoQ metabolite synthesis [167]; the major functional protein involved in this synthesis, CoQ protein, is localized in the mitochondria. The process involves the transport of metabolites from the mitochondria to the plasma membrane, facilitated by StAR-related lipid transfer protein 7 (STARD7). Finally, these metabolites are reduced by FSP1 to inhibit lipid peroxidation and ferroptosis [168]. Mitochondria play another crucial protective role in the decomposition of PUFAs, in which the mitochondrial enzyme 2,4-dienoyl-CoA reductase 1 (DECR1) predominantly mediates this process by reducing PUFAs to exert an anti-ferroptotic effect [169]. Notably, DECR1 has been further defined as an “androgen-repressed survival factor,” with androgen directly binding to the DECR1 promoter and suppressing its transcription. This regulatory mechanism is particularly pronounced in prostate cancer due to the central role of androgen signaling as the core oncogenic driver in this malignancy [170].

In summary, the regulation of ferroptosis by mitochondria exhibits different roles depending on the type of cell and biochemical processes involved. When the cytoplasmic anti-ferroptosis regulatory systems are disrupted, mitochondria may become more significant in the overall regulation of ferroptosis [171, 172].

**Endoplasmic reticulum**: The endoplasmic reticulum regulates ferroptosis not only by influencing the synthesis of polyunsaturated fatty acids but also by generating reactive oxygen species, which enhance the sensitivity to ferroptosis. POR: It is a membrane-bound enzyme belonging to the flavoprotein family, primarily found in the endoplasmic reticulum. It catalyzes the redox reaction of NADPH, providing electrons to cytochrome P450 enzymes. CYB5R1: It is a membrane-bound enzyme mainly located in the endoplasmic reticulum and mitochondria. It catalyzes the redox reaction of NADH, transferring electrons to cytochrome b5, thereby participating in various biochemical reactions. Research has found that POR and CYB5R1, retained in the endoplasmic reticulum, utilize NADPH to produce ROS, thereby promoting membrane lipid peroxidation [152].

The Mevalonate Pathway is a crucial metabolic pathway in the endoplasmic reticulum that synthesizes steroids, sterols, fatty acids, and other important biomolecules. Several intermediates in the cholesterol synthesis pathway play a significant role in inhibiting ferroptosis. 7-Dehydrocholesterol acts as an endogenous free radical scavenger, limiting lipid peroxidation in membranes [173]; farnesyl pyrophosphate is a key precursor in the synthesis of RTAs, vitamin K [174], and coenzyme Q [167]; isopentenyl pyrophosphate is involved in the isoprenylation of Sec-tRNA, which regulates the translation of selenoproteins, affecting the levels of GPX4, thereby influencing lipid peroxidation [175].

Nuclear Factor Erythroid 2-Related Factor 1 (NFE2L1) is an important transcription factor that resides in the endoplasmic reticulum. It undergoes complex post-translational modifications, including deglycosylation by N-Glycanase 1 (NGLY1) in the cytoplasm and proteolytic cleavage by DNA Damage Inducible 1 Homolog 2 (DDI2), ultimately leading to the generation of an active transcription factor [176]. The NGLY1–NFE2L1 axis indirectly promotes the stability of GPX4 protein, helping to suppress ferroptosis in certain cells, potentially by modulating the expression or function of proteasome genes [177].

**Fig. 3:**
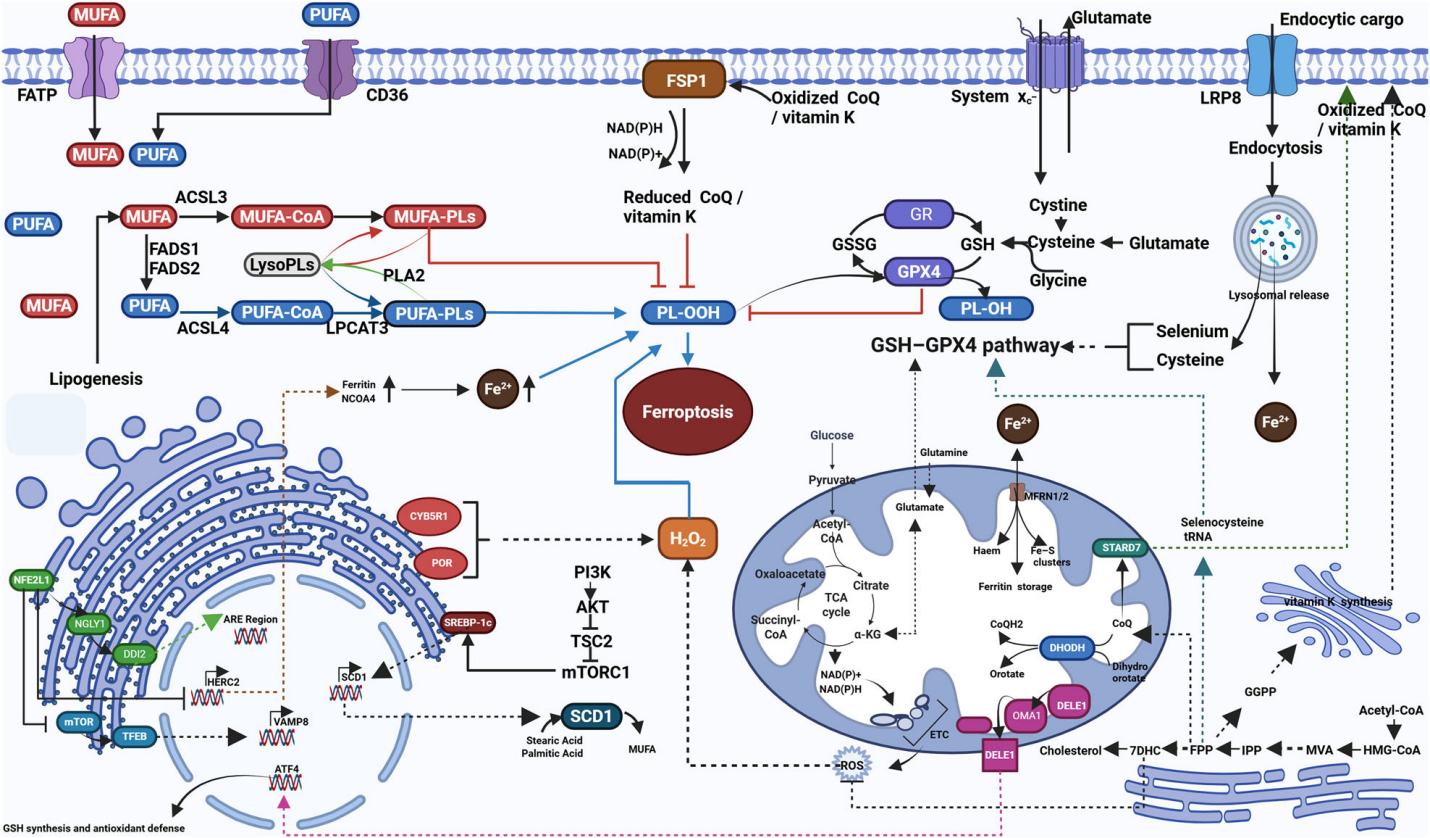
Regulatory mechanisms of ferroptosis. The occurrence of ferroptosis involves an increase in PUFAs, Fe²⁺, and reactive oxygen species levels. The synthesis of PUFAs primarily occurs in the endoplasmic reticulum. On one hand, the MUFAs ingested by cells are converted into MUFAs-CoA under the action of ACSL3, which then interacts with Lysop LS to produce MUFAs-PLs, thereby inhibiting PL-OOH. On the other hand, the PUFA intake is converted into FUFAs-CoA under the action of ACSL4, which also interacts with Lysop LS to form FUFAs-PLs, promoting PL-OOH. Additionally, MUFAs can be converted into PUFAs under the influence of FADS1 and FADS2, thus facilitating ferroptosis. Additionally, the transcription factors SREBPs, residing in the endoplasmic reticulum, translocate to the nucleus, activating the expression of SCD1, which catalyzes the synthesis of long-chain monounsaturated fatty acids, thereby reducing membrane susceptibility to peroxidation and sensitivity to ferroptosis. The generation of Fe²⁺ involves the endoplasmic reticulum, mitochondria, and lysosomes. In the endoplasmic reticulum, NFE2L2 acts on VAMP8-NCOA4 to increase free Fe²⁺ levels; iron entering the cell via TFRC is released in the acidic environment of the lysosome, resulting in elevated free Fe²⁺; and increased mitochondrial iron uptake mediated by MFRN1 in the mitochondria can enhance sensitivity to ferroptosis. The production and regulation of ROS primarily involve the antioxidant enzyme system, lipophilic free radical-scavenging antioxidants, and the regulation of ROS by certain organelles. Within the antioxidant enzyme system, GPX4 directly inhibits lipid peroxidation, with its synthesis dependent on cysteine and selenium, thus regulated by the cystine-glutamate reverse transport protein (xc-) that transports cystine, a precursor of cysteine. Additionally, some cysteine and selenium are derived from lysosomes, which are also regulated by lysosomes. Furthermore, selenocysteine is synthesized directly on a specific tRNA (tRNA[Ser]Sec) through a multi-step process that relies on FPP from the endoplasmic reticulum. The main RTAs include CoQ10 and vitamin K, whose reduced forms can reduce reactive oxygen species and inhibit ferroptosis. FSP1, located in the cell membrane, facilitates the reduction of CoQ10 and vitamin K, further inhibiting ferroptosis. In mitochondria, α-KG competes with GPX4 for cysteine, inhibiting ferroptosis; the activation of Oma1 leads to mitochondrial fragmentation and induces an integrated stress response along the Oma1-Dele1-Atf4 signaling axis, which can inhibit ferroptosis and exert a protective role. In the endoplasmic reticulum, POR and CYB5R1 utilize NADPH to produce ROS, promoting lipid peroxidation and ferroptosis; in the mevalonate pathway, 7DHC acts as an endogenous free radical scavenger, limiting lipid peroxidation; GGPP is a key precursor for the synthesis of vitamin K and coenzyme Q, inhibiting ferroptosis. PUFAs polyunsaturated fatty acids, MUFAs monounsaturated fatty acids, ACSL3 Acyl‑CoA synthetase long‑chain family member 3, MUFAs‑CoA monounsaturated fatty acyl‑CoA, PLA2 phospholipase A2, Lysop LS lysophospholipids (lysophospholipids are a form of phospholipids characterized by the retention of only one fatty acid chain in their structure, serving as critical intermediates in phospholipid metabolism), MUFAs‑PLs monounsaturated fatty acyl phospholipids, PL‑OOH phospholipid hydroperoxides, ACSL4 Acyl‑CoA synthetase long‑chain family member 4, FUFAs‑CoA fully unsaturated fatty Acyl-CoA, FUFAs‑PLs polyunsaturated fatty acyl phospholipids, FADS1 fatty acid desaturase 1, FADS2 fatty acid desaturase 2, SREBPs sterol regulatory element‑binding proteins, SCD1 stearoyl‑CoA desaturase 1, NFE2L2 nuclear factor, erythroid 2‑like 2 (NRF2), VAMP8 vesicle‑associated membrane protein 8, NCOA4 nuclear receptor coactivator 4, TFRC transferrin receptor, MFRN1 mitoferrin‑1 (SLC25A37), GPX4 glutathione peroxidase 4, FPP farnesyl pyrophosphate, RTAs radical‑trapping antioxidants, CoQ10 coenzyme Q10, FSP1 ferroptosis suppressor protein 1 (AIFM2), α‑KG alpha‑ketoglutarate (2‑oxoglutarate), OMA1 OMA1 zinc metallopeptidase (mitochondrial OMA1 protease), DELE1 DAP3‑binding cell death enhancer 1, ATF4 activating transcription factor 4, POR cytochrome P450 oxidoreductase, CYB5R1 cytochrome b5 reductase 1, NADPH nicotinamide adenine dinucleotide phosphate (reduced form), 7DHC 7‑dehydrocholesterol, GGPP Geranylgeranyl pyrophosphate.

The overall regulation of ferroptosis is shown in Fig. 3.

## Pyroptosis overview

Pyroptosis is a highly inflammatory form of programmed cell death, often referred to as “programmed inflammatory pyroptosis.” It is primarily activated by inflammasomes, which subsequently trigger the cleavage of gasdermin family proteins (such as caspase-1, caspase-4/5/11) to form pores in the cell membrane. This process ultimately leads to cell lysis and the release of a large number of inflammatory factors (such as IL-1β and IL-18) into the surrounding environment [178].

### NLRP3 inflammasome: a key regulator of inflammation

Inflammasomes are multi-protein complexes formed within cells, consisting of pattern recognition receptors (such as NLRP3, NLRC4, AIM2, etc.), apoptosis-associated speck-like protein containing a CARD (ASC), and effector proteins (such as caspase-1). They are capable of sensing a variety of pathogens, cellular damage signals, or metabolic abnormalities, which trigger the activation of downstream caspase-1. This activation subsequently cleaves and matures inflammatory factors, including IL-1β and IL-18, and can also activate pyroptosis and other pathways of inflammatory cell death, serving as a critical regulatory hub in the body’s innate immune response and inflammatory reaction [179]. The classic structure of the NLRP3 inflammasome (NOD-Like Receptor Protein 3 Inflammasome) comprises three parts: a central nucleotide-binding oligomerization domain (referred to as NACHT), a C-terminal leucine-rich repeat (LRR) domain, and an N-terminal effector domain (the pyrin domain, or PYD), which interacts with downstream signaling molecules to promote pyroptosis. Activated NLRP3 binds to an adapter protein—ASC, which contains a caspase-recruitment domain—facilitating the aggregation of ASC into stable filamentous structures. This assembly supports the formation of multiple star-shaped caspase-1 filaments, increasing the density of caspase-1 to enhance contact with substrates [180].

### Apoptosis regulatory effector: caspase-1

Caspase-1 is an effector enzyme of pyroptosis, promoting the maturation and secretion of potent pro-inflammatory cytokines such as IL-1β and IL-18 in humans and mice, as well as a form of inflammatory lytic cell death known as “pyroptotic cell death.” Human caspase-4 and caspase-5, along with their murine homolog caspase-11, mediate the activation of noncanonical inflammasomes. On one hand, caspase-1 cleaves and converts pro-IL-1β and pro-IL-18 into their active forms; on the other hand, it cleaves gasdermin D (GSDMD), leading to the oligomerization of the GSDMD N-terminal fragment (GSDMD-NT) [181]. GSDMD-NT forms 21-nanometer pores on the plasma membrane, driving the extracellular release of IL-1β and IL-18, leading to the loss of membrane integrity and ultimately resulting in cell death.

### Hallmarks of pyroptosis executioner: GSDMD

GSDMD is a member of the Gasdermin protein family and is closely related to pyroptosis, serving as one of the important molecules for the body’s defense against pathogens and for mediating inflammatory responses. Research on the pore structure of GSDMD-NT has shown that it facilitates the passage of neutral and positively charged proteins while reducing the transport of negatively charged proteins, thereby promoting the passage of mature IL-1β and IL-18, and increasing the cytoplasmic persistence of acidic, negatively charged pro-IL-1β and pro-IL-18. GSDMD-NT has a high affinity for phosphatidylinositol phosphates and phosphatidylserine, which are primarily located on the inner surface of the plasma membrane, thereby minimizing damage to adjacent cellular membranes [182]. At lower concentrations, GSDMD-NT forms non-lytic pores, which are more conducive to the release of pro-inflammatory cytokines rather than to cell death, compared to higher concentrations [183].

### Regulatory mechanisms of pyroptosis execution

#### The occurrence of pyroptosis mediated by damage-associated molecular patterns or pathogen-associated molecular patterns

The activation of the NLRP3 inflammasome in cardiomyocytes and other resident cardiac cells is a prerequisite for pyroptosis. However, in healthy, undamaged hearts, the expression of NLRP3 inflammasome components is very low, thus insufficient to promote their aggregation and accumulation. Damage-associated molecular patterns (DAMPs) refer to a series of molecular signals released by the body’s own cells or tissues in response to damage, pyroptosis, or stress, such as ATP, heat shock proteins, and high mobility group proteins. These molecules are typically exposed or released into the extracellular environment only when normal physiological processes are disrupted or the integrity of the cell membrane is compromised. At this point, immune cells can recognize DAMPs through pattern recognition receptors, thereby activating immune and inflammatory responses [184]. Pathogen-Associated Molecular Patterns (PAMPs) refer to molecular structures derived from microbial pathogens, such as bacteria, viruses, fungi, and parasites, including bacterial lipopolysaccharides and peptidoglycans, and viral double-stranded RNA. The immune system detects PAMPs through various pattern recognition receptors, such as Toll-like receptors (TLRs) and NOD-like receptors (NLRs), to initiate an immune response that aids in pathogen clearance. In fact, DAMPs and PAMPs engage Toll-like receptors (TLRs) or Nucleotide-Binding Oligomerization Domain-Containing 2 (NOD2), activating NF-κB transcriptional regulation, which enhances the levels of the NLRP3 inflammasome in cells. Once a critical mass of NLRP3 inflammasome components is reached, the accumulation of activation signals (or triggers) leads to potassium efflux within the cell, thereby catalyzing the formation of the inflammasome [185]. In addition, the extracellular ATP binding to purinergic P2X7 receptors causes K^+^ efflux; lysosomal destabilization induced by cholesterol, urate crystals, or pathogens can also lead to the leakage of cytochrome c and K^+^ efflux, thereby activating the NLRP3 inflammasome and promoting pyroptosis [186, 187].

#### Other regulatory mechanisms of thermal runaway

NIMA-related kinase 7 (NEK7) is a serine/threonine kinase that was initially thought to play a primary role in regulating the assembly and rearrangement of microtubules during cell cycle division. Subsequent research has revealed that NEK7 is also critically involved in immune responses, particularly closely associated with the activation of the NLRP3 inflammasome. Studies have shown that NEK7 can sense K^+^ efflux, bind to NLRP3, and induce its aggregation [188]. BTK is a non-receptor tyrosine kinase that is primarily expressed in B cells and myeloid cells. It is also essential for the activation of NLRP3 in the body and interacts with ASC [189].

Thioredoxin-interacting protein (TXNIP) is a protein that can bind to thioredoxin (Trx) and inhibit its activity. It plays an important role in redox balance, metabolic regulation, and inflammatory responses [190]. Under healthy conditions, TXNIP binds to thioredoxin; however, increased levels of and the unfolded protein response lead to the release of TXNIP from thioredoxin, promoting the activation of NLRP3 and subsequent inflammasome formation [191].

Autophagy is an evolutionarily conserved catabolic pathway whereby cells, in response to stimuli such as nutrient deficiency, oxidative stress, or pathogen infection, encapsulate damaged or excess organelles and proteins and degrade them through fusion with lysosomes to obtain energy and raw materials. In human monocytes and macrophages, the inhibition of the autophagy pathway drives the formation of the NLRP3 inflammasome, while increased autophagic flux reduces the secretion of IL-1β, suggesting the role of autophagy impairment in driving inflammasome activation [192]. Mitochondrial dysfunction and damage are also major factors that activate NLRP3-mediated pyroptosis. For instance, ineffective mitophagy leads to increased ROS production and the accumulation of mitochondrial DNA in the cytoplasm, which enhances the activation of caspase-1 and IL-1β. Knocking out genes related to mitochondrial fission promotes NLRP3-dependent caspase-1 activation and IL-1β secretion; conversely, mitochondrial fusion has been shown to prevent the activation of NLRP3 [193, 194].

**Fig. 4:**
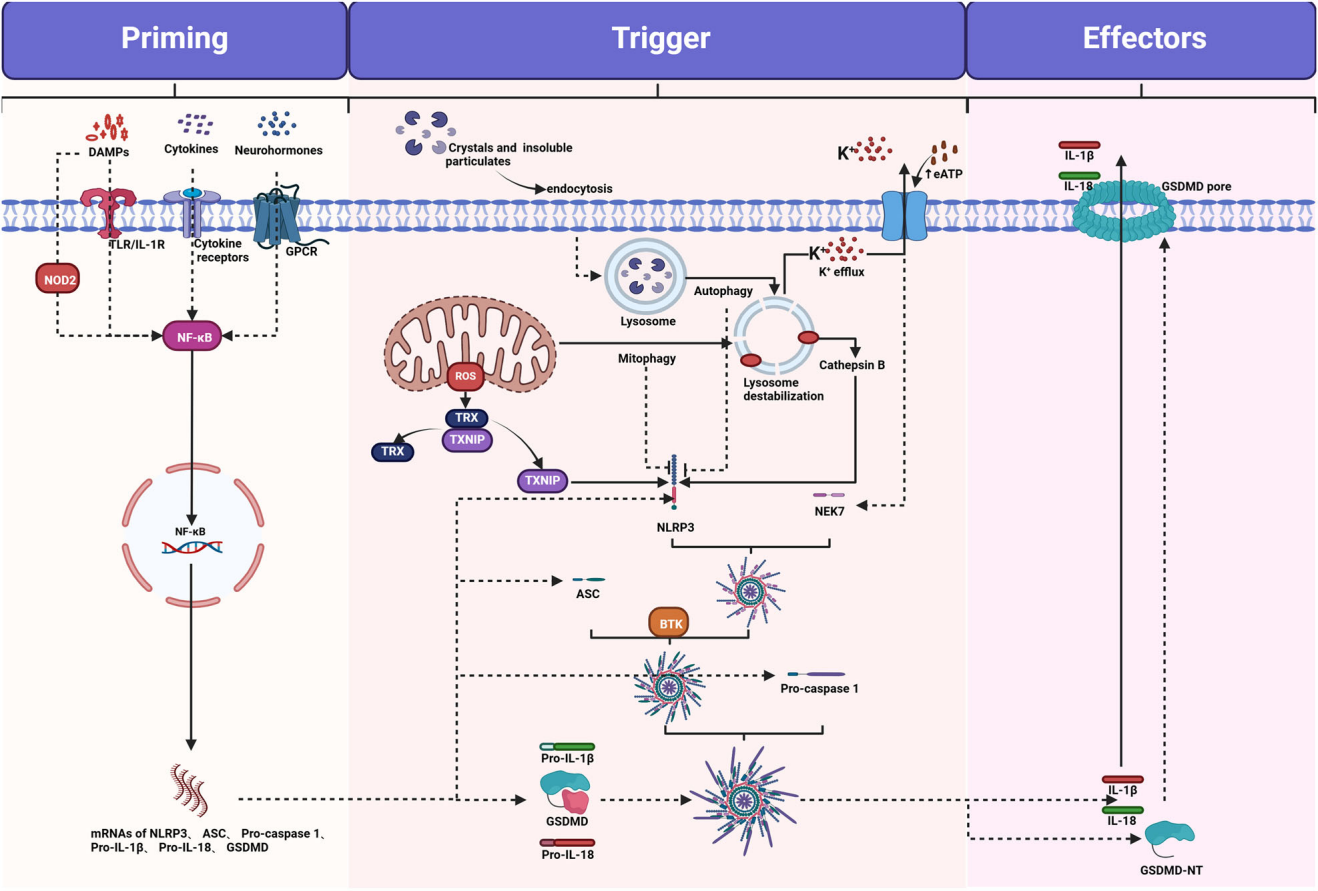
Regulation mechanism of pyroptosis. Pyroptosis primarily consists of three stages. First, DAMPs (ATP, heat shock proteins, high mobility group box 1 (HMGB1)), PAMPs (LPS, peptidoglycan, viral double-stranded RNA), cytokines, neurohormones, etc., enter the cell through receptors such as TLR/IL-1R, cytokine receptors, and GPCRs, activating NF-κB, which in turn promotes the transcription of mRNA for NLRP3, ASC, pro-caspase-1, pro-IL-1β, pro-IL-18, GSDMD, among others. Following the translation, the NLRP3 protein forms a complex with NEK7, which, under the influence of BTK, further interacts with ASC to create the second phase of the complex. This complex continues to associate with Pro-caspase-1, culminating in the formation of the inflammasome. The inflammasome senses various pathogens, cellular damage signals, or metabolic abnormalities, triggering the activation of downstream caspase-1. Caspase-1 cleaves and converts pro-IL-1β and pro-IL-18 into their active forms, while also cleaving GSDMD to generate GSDMD-NT. During this phase, both autophagy and mitophagy inhibit the generation of NLRP3. However, elevated levels of ROS and the unfolded protein response lead to the release of TXNIP from thioredoxin, promoting the activation of NLRP3 and the subsequent formation of the inflammasome. Additionally, Cathepsin B within the lysosome also facilitates the generation of NLRP3. In the final stage, GSDMD-NT forms 21-nanometer pores, driving the extracellular release of IL-1β and IL-18, as well as the loss of membrane integrity, ultimately leading to cell death and the release of inflammatory mediators. DAMPs damage-associated molecular patterns, PAMPs pathogen-associated molecular patterns, LPS lipopolysaccharide, TLR/IL-1R toll-like receptor/Interleukin-1 receptor, GPCR G protein-coupled receptor, NF-κB nuclear factor kappa-light-chain-enhancer of activated B cells, NLRP3 NOD-like receptor family, pyrin domain-containing 3, ASC apoptosis-associated speck-like protein containing a CARD, Pro-IL-1β pro-interleukin-1 beta, Pro-IL-18 pro-interleukin-18, GSDMD gasdermin D, NEK7 NIMA-related kinase 7, BTK Bruton tyrosine kinase, IL-1β interleukin-1 beta, IL-18 interleukin-18, GSDMD-NT N-terminal fragment of gasdermin D, TXNIP thioredoxin-interacting protein.

The overall regulatory state of pyroptosis is shown in Fig. 4.

## Acetylation-mediated regulation of cell death pathways

### Acetylation-mediated apoptosis

Apoptosis mediated by acetylation primarily operates through this modification’s regulation of the expression of apoptotic genes or proteins, thereby promoting or inhibiting the occurrence of apoptosis.

#### Acetylation modifications regulating the expression of pro-apoptotic/anti-apoptotic genes

##### Acetylation modification of the BIK gene expression

**Fig. 5:**
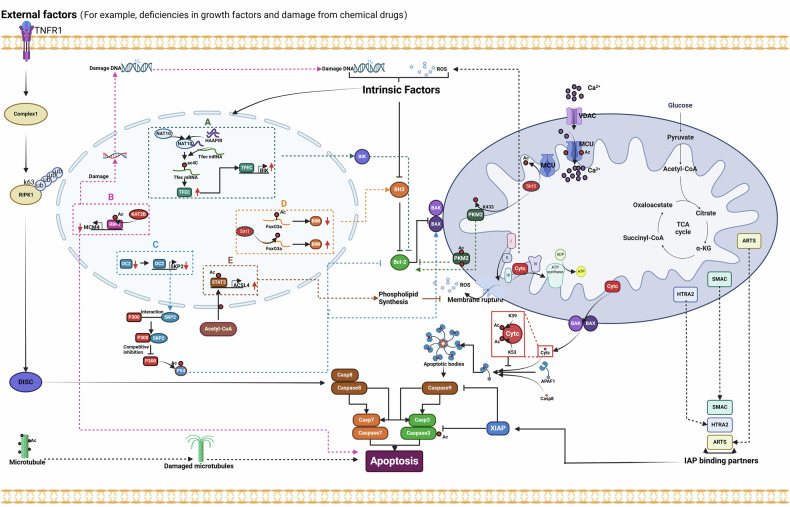
Acetylation regulatory mechanism of apoptosis. **A** HAAPIR and NAT10 acetylate Tfec mRNA transcripts through ac4C, which promotes the expression of the BIK gene, facilitating apoptosis. **B** KAT2B can bind to the transcriptional repressor RBPJ while increasing the acetylation level of RBPJ. Acetylated RBPJ binds to the promoter of MCM4, inhibiting the expression of the MCM4 gene, thereby leading to DNA damage and promoting apoptosis. **C** OC2 can directly bind to the promoter of SKP2 and regulate its expression; downregulation of OC2 also results in decreased SKP2 expression. The downregulation of both releases p300, thereby promoting the acetylation of P53 and further facilitating apoptosis. **D** When Sirt1 expression significantly decreases, the deacetylation level of FoxO3a is restricted. Hyperacetylated FoxO3a leads to a decrease in BIM expression, promoting apoptosis. **E** FAO increases CoA, triggering the acetylation of STAT3, and acetylated STAT3 upregulates the expression of ACSL4, which in turn leads to increased phospholipid synthesis and enhances mitochondrial integrity, thereby overcoming cellular apoptosis. In mitochondria, PKM2 is a key metabolic enzyme. When PKM2 accumulates in the mitochondria and undergoes hyperacetylation, it leads to a decrease in Bcl-2 expression. The action of SIRT3 on the K433 residue of PKM2 reduces its acetylation level, thereby stabilizing Bcl-2 expression and exerting an anti-apoptotic effect. Additionally, the MCU with high acetylation levels is in an activated state, resulting in a substantial influx of Ca^2+^ into the mitochondria, which causes mitochondrial Ca²⁺ overload and depolarization, ultimately triggering apoptosis. Acetylation of Cytc at K39 and K53 reduces its binding with Apaf-1, thereby inhibiting apoptosis. Furthermore, when the level of α-tubulin acetylation reaches a certain threshold, cells undergo apoptosis rather than autophagy. HAAPIR heterogeneous nuclear ribonucleoprotein A1-associated protein interacting with RNA, NAT10 N-acetyltransferase 10, ac4C N-acetyltransferase 4C, Tfec transcription factor EC, BIK Bcl-2 interacting killer, KAT2B lysine acetyltransferase 2B, RBPJ recombination signal binding protein for immunoglobulin kappa J region, MCM4 minichromosome maintenance 4, OC2 Onecut2, SKP2 S-phase kinase-associated protein 2, p300 E1A-associated protein p300, Sirt1 sirtuin 1, FoxO3a forkhead box O3, BIM Bcl-2 interacting mediator of cell death, FAO fatty acid oxidation, CoA coenzyme A, STAT3 signal transducer and activator of transcription 3, ACSL4 Acyl-CoA synthetase long-chain family member 4, PKM2 pyruvate kinase M2, Bcl-2 B-cell lymphoma 2, SIRT3 sirtuin 3, MCU mitochondrial calcium uniporter, Cytc cytochrome c, Apaf-1 apoptotic protease-activating factor 1.

Heterogeneous Nuclear Ribonucleoprotein A1-Associated Protein Interacting with RNA (HAAPIR) is a protein that interacts with RNA and is primarily involved in post-transcriptional regulation and processing of RNA, with high expression levels in cardiomyocytes. In patients with myocardial infarction, HAAPIR enhances the acetylation of the transcription factor EC (Tfec) mRNA transcript via N4-acetylcytidine (ac4C) mediated by N-acetyltransferase 10 (NAT10), thereby increasing Tfec mRNA stability and translation, and promoting the TFEC–BIK pathway to drive cardiomyocyte apoptosis [195] (Fig. 5A, Top left).

##### Acetylation modifications of the expression of the DNA damage-related gene MCM4

Minichromosome Maintenance 4 (MCM4) is a protein that plays a crucial role in DNA replication, primarily ensuring the accurate duplication of DNA and the proper progression of the cell cycle. In patients with endometrial cancer, the lysine acetyltransferase KAT2B can bind to the transcriptional repressor RBPJ, simultaneously increasing the acetylation level of RBPJ. Acetylated RBPJ binds to the promoter of MCM4, inhibiting the expression of the MCM4 gene. This inhibition subsequently leads to the suppression of cell proliferation, resulting in DNA damage and promoting apoptosis [196] (Fig. 5B, Left center).

##### Acetylation regulation of the P53 gene expression

Onecut2 (OC2) plays a crucial regulatory role in tumor growth, metastasis, and angiogenesis. In hepatocellular carcinoma, the knockout of OC2 using the CRISPR/Cas9 system not only significantly inhibits the proliferation and angiogenesis of hepatocellular carcinoma cells but also markedly promotes apoptosis. ChIP-Seq and dual-luciferase reporter assays demonstrate that OC2 can directly bind to the promoter of SKP2 and regulate its expression. When OC2 is downregulated, SKP2 expression also decreases, and the downregulation of both releases p300, thereby promoting the acetylation of P53 and further enhancing apoptosis [197] (Fig. 5C, Lower left).

##### Acetylation modification of the BIM gene expression

Forkhead box O3a (FoxO3a) is an important transcription factor belonging to the FoxO family, and is widely involved in various biological processes such as cell growth, metabolism, apoptosis, and stress responses. In myocardial infarction, the expression of the deacetylase Sirt1 is significantly reduced, limiting the deacetylation of FoxO3a. As a result, the K271/K290 sites of FoxO3a exhibit high acetylation levels, leading to decreased expression of BIM (a BH3-only protein with the characteristic BH3 domain of the Bcl-2 family that binds to anti-apoptotic proteins, inhibits their function, and thereby promotes the activation of pro-apoptotic proteins such as Bax and Bak), which in turn facilitates the occurrence of apoptosis [198] (Fig. 5D, Middle).

In urinary and male reproductive tract infections caused by uropathogenic Escherichia coli (UPEC), UPEC suppresses the host cell AKT signaling pathway via its virulence factor α-hemolysin, resulting in a marked reduction in histone H4 acetylation levels (ChIP-qPCR shows *P* < 0.001 compared with the control group). Although the specific acetylation sites were not identified, this change epigenetically silences BIM expression, thereby blocking caspase-3 activation and apoptosis, and promoting bacterial immune evasion and chronic infection [199]. In triple-negative breast cancer (TNBC), treatment with EZH2 and HDAC inhibitors (such as GSK126 and LBH589) increases histone H3 lysine 27 acetylation (H3K27ac) levels by 2.5–4-fold (ChIP-qPCR, *P* < 0.05), acting specifically on the promoter and enhancer regions of the BIM gene. This upregulates BIM expression, induces cancer cell apoptosis, and provides a new mechanism for targeted therapy in TNBC [200].

##### Acetylation modifications of the Bcl-2 gene expression

Pyruvate Kinase M2 (PKM2) is a key metabolic enzyme primarily involved in the glycolytic process. Recent studies have shown that PKM2 not only plays a significant role in energy metabolism but is also closely related to apoptosis. In research on pulmonary ischemia/reperfusion injury, PKM2 accumulates in the mitochondria and undergoes hyperacetylation, leading to a decrease in Bcl-2 expression. The deacetylase SIRT3 acts on the K433 residue of PKM2, resulting in reduced acetylation levels of PKM2, thereby stabilizing Bcl-2 expression and exerting an anti-apoptotic effect [201].

Idiopathic pulmonary fibrosis (IPF) is a progressive lung disease in which fibroblast apoptosis resistance drives pathological fibrosis. This study reveals that anti-HSP70 autoantibodies in patients with IPF enhance apoptosis resistance via an epigenetic mechanism—specifically, treatment with these autoantibodies markedly increases acetylation of lysine 16 on histone H4 (H4K16Ac) at the Bcl-2 gene promoter (*p* < 0.05), thereby upregulating Bcl-2 expression and suppressing apoptosis. This effect depends on the acetyltransferase MOF; its knockdown reverses the process, confirming that the autoantibodies promote IPF progression through H4K16Ac-mediated epigenetic regulation [202].

#### Acetylation modifications of key proteins regulating apoptosis

##### Acetylation modification of caspase-3 protein expression

The N-terminus of a protein carries information about its biochemical properties and functions. These N-termini can be processed by proteases and can undergo other co-translational or post-translational modifications. For example, in HCT116 cells, 74% of ORF-derived N-terminal peptides are modified by N-terminal acetylation. Caspase-3 is a key executioner protease in apoptosis and plays an important role during programmed cell death. Studies have shown that newly generated N-termini created by Caspase-3 cleavage (such as Ser43 on the NACA protein and Ser94 on the eIF4H protein) can be further modified by N-terminal acetylation (a total of 358 new sites identified across 318 proteins). Moreover, this modification occurs at early stages of apoptosis (for instance, acetylation of NACA Ser43 is detectable 1 h after ABT-199 treatment) and may contribute to translational repression by stabilizing protein fragments and regulating ribosome function [203].

##### Acetylation modification of Cytc protein expression

Cyt c is a small, essential protein composed of 104 amino acids, with dual functions related to sustaining life and cell death. Within the mitochondria, Cyt c acts as a single-electron carrier as part of the Electron Transport Chain (ETC); however, when the outer mitochondrial membrane is ruptured, Cyt c is released into the cytoplasm, triggering the assembly of apoptosomes and initiating apoptosis. Therefore, the regulation of Cyt c is of significant importance, and post-translational modification is a key intervention strategy. Six phosphorylation sites, T28, S47, Y48, T49, T58, and Y97, as well as three acetylation sites, K8, K39, and K53, have been identified to regulate Cytc in vivo [204]. In skeletal muscle subjected to ischemia-reperfusion injury, acetylation of Cytc at K39 and the K39Q acetyl mimic substitute reduce the binding to Apaf-1, as well as the cleavage and activity of caspases, thereby inhibiting apoptosis [205]. In prostate cancer, the lysine 53 (K53) of Cytc undergoes significant acetylation. By expressing the acetylation-mimicking mutant K53Q in Cytc double knockout cells, the apoptotic phenotype is improved, demonstrating that K53 acetylation of Cytc is an adaptive response [206].

#### Acetylation modification in non-classical apoptosis pathways

##### Acetylation modifications induced by calcium overload leading to apoptosis

The integrity of the mitochondrial membrane is a necessary condition for preventing the intrinsic apoptotic pathway. In addition to the regulation of MOMP by BCL-2 family proteins, which leads to the release of Cytc into the cytosol, sustained high levels of mitochondrial calcium overload, as mentioned in the overview of apoptosis, can also induce the opening of the mPTP, resulting in rupture of the OMM and the release of Cytc. However, the influx of Ca^2+^ through the IMM requires the opening of the MCU. Research has shown that MCU remains inactive under resting cellular conditions and is only activated when local Ca^2+^ levels rise above 1 μM [207]. A study on colorectal cancer found that MCU is regulated by acetylation modifications. The inhibition of SIRT1 leads to increased acetylation at the K332 residue. High levels of acetylated MCU exhibit an activated state, resulting in a significant influx of Ca^2+^ into the mitochondria, causing mitochondrial Ca²⁺ overload and depolarization, ultimately triggering apoptosis in colorectal cancer cells [208]. Another factor affecting mitochondrial membrane integrity is the phospholipid synthesis pathway, as phospholipids are fundamental components of cell membranes. Some apoptosis occurs due to the disruption of mitochondrial membrane structure, while increased phospholipid synthesis contributes to the integrity of the mitochondrial membrane, thereby enhancing its anti-apoptotic effects. A study addressing the chemotherapy-induced apoptosis of tumor cells confirmed that fatty acid β-oxidation triggers the acetylation of STAT3 by increasing acetyl-CoA. The acetylated STAT3 upregulates the expression of ACSL4, which leads to increased phospholipid synthesis and enhances mitochondrial integrity, thus overcoming chemotherapy-induced tumor cell apoptosis [209] (Fig. 5E, Lower left).

##### Acetylation of microtubule proteins associated with apoptosis

Alpha-tubulin is a major component of microtubules, which are essential elements of the cytoskeleton involved in various biological processes such as maintaining cell shape, cell division, and material transport. Depolymerization or instability of microtubules can induce apoptosis. For example, microtubule-targeting agents (such as phenethyl isothiocyanate in combination with paclitaxel) can increase microtubule acetylation levels by approximately 6–10 times; acetylation enhances microtubule stability and induces cell cycle arrest and apoptosis [210]. In lung cancer research, the acetyltransferase TAT1, the deacetylase HDAC6, and Hsp90 regulate the expression level of α-tubulin Lys40 acetylation. When α-tubulin acetylation reaches a certain level, cells undergo apoptosis rather than autophagy, suggesting that the level of acetylated α-tubulin may determine a cell’s survival or apoptotic fate [211]. However, the article lacks specific quantitative information, which diminishes the credibility of this “threshold” effect.

A summary of acetylation modifications regulating the expression of apoptosis-related genes/proteins is presented in Table 3.

**Table 3:** Acetylation modifications regulating apoptosis.

Gene/Protein name	Acetylation modification sites	Acetylation regulatory mechanisms	Biological function (with reference numbers)
**p53**	Sites not specified	Quantitative data not provided	Acetylation state regulates stability and transcriptional activity; promotes **apoptosis** [197].
**STAT3**	Sites not specified	Acetylation enhances transcriptional activity.	Acetylated STAT3 upregulates ACSL4 expression, **promoting apoptosis** [209].
**FoxO3a**	K271, K290	High acetylation levels when Sirt1 expression is significantly reduced.	Hyperacetylation decreases BIM expression, **promoting apoptosis** [198].
**MCU**	K332	SIRT1 inhibition increases acetylation; highly acetylated MCU is activated.	Activation causes Ca²⁺ influx into mitochondria, leading to calcium overload and depolarization, **triggering apoptosis** [208].
**Cyt c**	K39, K53	K39 acetylation mimic (K39Q) reduces Apaf-1 binding; K53 acetylation mimic (K53Q) improves apoptotic phenotype.	Acetylation (especially K39 and K53) reduces Cytc binding to Apaf-1 and caspase activation, **inhibiting apoptosis** [204–206].
**α-tubulin**	K40	Microtubule-targeting agents increase acetylation ~6–10 fold; evodiamine (5 μM) increases acetylation ~3–4 fold.	Acetylation enhances microtubule stability. At certain thresholds, it triggers **apoptosis** rather than autophagy [210, 211].
**RBPJ**	Sites not specified	KAT2B increases its acetylation level.	Acetylated RBPJ binds the MCM4 promoter, inhibiting MCM4 expression, causing DNA damage, and **promoting apoptosis** [196].
**Caspase-3**	Newly generated N-termini (e.g., NACA Ser43, eIF4H Ser94)	In HCT116 cells, 74% of ORF-derived N-terminal peptides are N-terminally acetylated; NACA Ser43 acetylation is detectable 1 h after ABT-199 treatment.	N-terminal acetylation of caspase-cleaved protein fragments may stabilize fragments and regulate ribosome function, contributing to translational repression early in **apoptosis** [203].
**TFEC (mRNA)**	Sites not specified	Quantitative data not provided	Enhances Tfec mRNA stability and translation, promoting the TFEC–BIK pathway to **drive cardiomyocyte apoptosis** [195].
**BIM (gene)**	H3K27	Treatment with EZH2 and HDAC inhibitors (such as GSK126 and LBH589) increases histone H3 lysine 27 acetylation (H3K27ac) levels by 2.5–4-fold	The promoter and enhancer regions of the BIM gene, thereby upregulating BIM expression and inducing **apoptosis** [200].
**Bcl-2 (gene)**	H4K16	Treatment with anti-HSP70 autoantibodies significantly increases the H4K16Ac level in the promoter region of the Bcl-2 gene by affecting the acetyltransferase MOF	Upregulated Bcl-2 **suppresses apoptosis** [202].

*p53* tumor protein p53, *STAT3* signal transducer and activator of transcription 3, *FoxO3a* forkhead box O3a, *MCU* mitochondrial calcium uniporter, *Cyt c* cytochrome c, *α-tubulin* alpha-tubulin, *RBPJ* recombination signal binding protein for immunoglobulin kappa J region, *Caspase-3* cysteine-dependent aspartate-specific protease 3, *TFEC* transcription factor EC, *SIRT1* sirtuin 1, *KAT2B* lysine acetyltransferase 2B, *Apaf-1* apoptotic protease-activating factor 1, *BIM* BCL-2 like 11, *BIK* BCL-2 interacting killer.

### Acetylation-mediated ferroptosis

The mechanisms of ferroptosis are relatively complex. Acetylation modifies the expression of ferroptosis-related genes by regulating ROS levels (SLC7A11, GPX4, FSP1) and iron metabolism pathways (FTH1). Modifications of ferroptosis-related protein expression act by regulating ROS levels (SLC7A11, GPX4, FSP1, DHODH, SDH) and by directly promoting the peroxidation of membrane phospholipids (ALOX12).

#### Acetylation modifications regulating the expression of ferroptosis-related genes

##### Acetylation modification of the SLC7A11 gene expression

Solute Carrier Family 7 Member 11 (SLC7A11), also known as System X-, is an important transporter protein primarily responsible for transporting cysteine from the extracellular space into the cell while simultaneously exporting glutamate to the extracellular space. Intracellular cysteine provides the substrate for GPX4 production. Currently, the acetylation modifications regulating SLC7A11 gene expression are as follows.

**Fig. 6:**
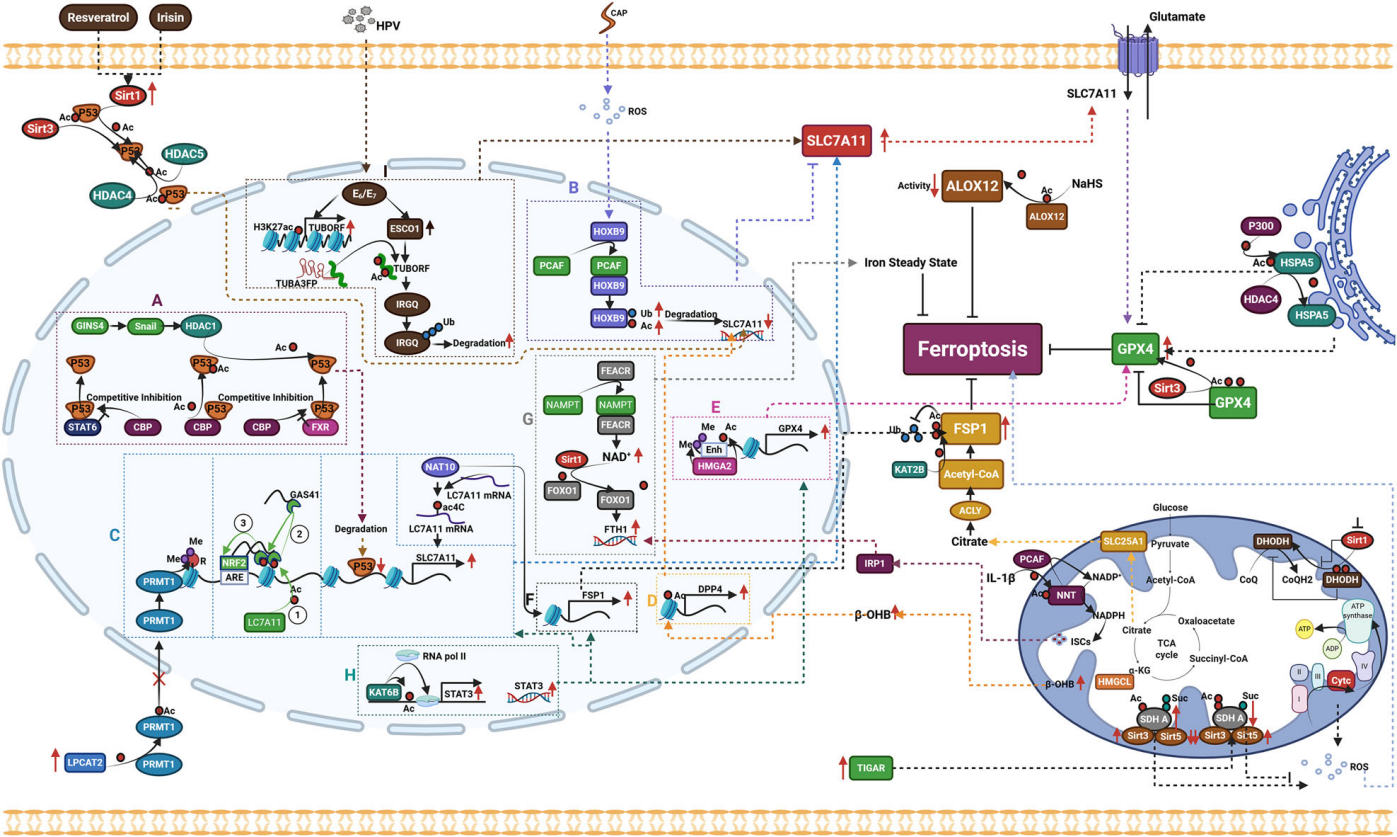
Mechanism of acetylation regulation of ferroptosis. **A** Acetylated P53 is not easily degraded and downregulates the expression of SLC7A11, inhibiting ferroptosis; Irisin and resveratrol entering the cell can elevate the level of Sirt1, leading to the deacetylation of P53. Additionally, Sirt3, HDAC4, and HDAC5 in the cytoplasm can also deacetylate P53, upregulating SLC7A11 and inhibiting ferroptosis. FXR and STAT6 undergo competitive inhibition with CBP, reducing the acetylation of p53, thereby upregulating the expression of SLC7A11 and inhibiting ferroptosis. **B** CAP enhances the acetylation modification level of HOXB9 by promoting the interaction between HOXB9 and PCAF; acetylated HOXB9 elevates its ubiquitination modification level and leads to its degradation, decreasing HOXB9’s ability to regulate LC7A11 expression as a transcription factor, promoting ferroptosis. **C** LPCAT2 regulates the acetylation of PRMT1 at the K145 site, preventing the cytoplasmic transport of PRMT1 within the cell, resulting in a loss of PRMT1-mediated asymmetric dimethylation of arginine 3 on the H4 histone of LC7A11, limiting transcriptional activation of LC7A11 and promoting ferroptosis. The acetylation of the H3 lysine 27 site of LC7A11 serves as a marker that recruits GAS41; GAS41 activates the expression of LC7A11 by bridging NRF2 with the H3K27ac site, inhibiting ferroptosis. NAT10 suppresses ferroptosis by stabilizing SLC7A11 mRNA through acetylation. **D** HMGCL modulates H3K9 acetylation via β-OHB and dose-dependently regulates the expression of DPP4, which inhibits SLC7A11 expression to control ferroptosis. E HMGA2 is an important transcription factor that binds to and promotes cis-element modifications in the GPX4 gene promoter region, enhancing enhancer activity by increasing H3K4 methylation and H3K27 acetylation. **F** NAT10 inhibits ferroptosis by stabilizing FSP1 mRNA transcripts through ac4C RNA modification. **G** FEACR binds to NAMPT, stabilizing the latter; NAMPT increases NAD^+^ synthesis, promoting Sirt1 expression, which in turn reduces FOXO1 acetylation levels. The latter upregulates the transcription of FTH1, inhibiting ferroptosis. **H** KAT6B induces acetylation of H3 lysine 23 in cells and enriches RNA pol II at the STAT3 promoter; STAT3 can bind to consensus DNA response elements in the promoters of GPX4, SLC7A11, and FTH1, regulating ferroptosis. **I** The HPV oncogenes E6 and E7 upregulate TUBORF on one hand through p300-mediated acetylation of histone H3 lysine 27; on the other hand, they recruit ESCO1 to bind and acetylate TUBORF, which subsequently leads to the degradation of IRGQ via the ubiquitin–proteasome pathway. The inhibition of IRGQ upregulates the expression of SLC7A11 and GPX4 proteins, ultimately enhancing resistance to ferroptosis. In the endoplasmic reticulum, the EP300 acetyltransferase promotes ferroptosis by acetylating the K353 site of HSPA5, while HDAC6 limits the acetylation of HSPA5 and the subsequent ferroptosis. The mechanism involves HSPA5 mediating resistance to ferroptosis by maintaining the stability of the GPX4 protein. SIRT3 also directly participates in the acetylation modification of GPX4. When SIRT3 expression decreases, GPX4 becomes highly acetylated, leading to a reduction in GPX4 protein levels and promoting ferroptosis. In mitochondria, SLC25A1 drives the transport of citrate from the mitochondria to the cytoplasm and provides energy for ACLY to synthesize acetyl-CoA. This, together with KAT2B, maintains the acetylation of FSP1, preventing its ubiquitination and subsequent degradation, while HDAC3 reverses this process. The reduction of mitochondrial SIRT3 leads to an increase in the acetylation level of DHODH; high acetylation levels of DHODH hinder pyrimidine biosynthesis and the production of CoQH2, thereby promoting ferroptosis. TIGAR translocates to the mitochondria, enhancing the interaction between SIRT5 and SDH A while reducing the interaction between SIRT3 and SDH A, facilitating the acetylation and deacylation of SDH A, which inhibits SDH activity, subsequently decreasing ROS production and suppressing ferroptosis. Furthermore, NaHS can alleviate the acetylation of ALOX12 and protect membrane lipids from peroxidation, thereby inhibiting ferroptosis. SLC7A11 solute carrier family 7 member 11, Sirt1 sirtuin 1, Sirt3 sirtuin 3, HDAC4 histone deacetylase 4, HDAC5 histone deacetylase 5, FXR farnesoid X receptor, STAT6 signal transducer and activator of transcription 6, CBP CREB-binding protein, CAP cold atmospheric plasma, HOXB9 homeobox B9, PCAF P300/CBP-associated factor, LPCAT2 lysophosphatidylcholine acyltransferase 2, PRMT1 protein arginine methyltransferase 1, GAS41 Gastric cancer associated protein 41, NRF2 nuclear factor erythroid 2-related factor 2, NAT10 N-acetyltransferase 10, HMGCL hydroxymethylglutaryl-CoA lyase, β-OHB beta-hydroxybutyrate, DPP4 dipeptidyl peptidase-4, HMGA2 high mobility group AT-hook 2, GPX4 glutathione peroxidase 4, FSP1 ferroptosis suppressor protein 1, FEACR ferroptosis-related Acyl-CoA binding protein, NAMPT nicotinamide phosphoribosyltransferase, FOXO1 forkhead box O1, FTH1 ferritin heavy chain 1, KAT6B lysine acetyltransferase 6B, STAT3 signal transducer and activator of transcription 3, p300 E1A-binding protein p300, TUBORF tubulin oligomerization factor, ESCO1 establishment of sister chromatid cohesion 1, IRGQ immunity-related GTPase Q, EP300 E1A-binding protein p300, HSPA5 heat shock protein family A member 5, HDAC6 histone deacetylase 6, SLC25A1 solute carrier family 25 member 1, ACLY ATP citrate lyase, KAT2B lysine acetyltransferase 2B, HDAC3 histone deacetylase 3, DHODH dihydroorotate dehydrogenase, CoQH2 coenzyme Q hydroquinone, TIGAR TP53-inducible glycolysis and apoptosis regulator, SIRT5 sirtuin 5, SDHA succinate dehydrogenase A, ALOX12 arachidonate 12-lipoxygenase.

**Regulation of acetylation modifications in the P53 pathway:** SLC7A11 is an important protein that inhibits ferroptosis, regulating multiple pathways, with the classic being the P53 pathway (Fig. 6A, Left center). SIRT is an NAD^+^-dependent deacetylase that plays a crucial role in the deacetylation modification of P53. Studies have found that irisin, a cleaved peptide containing the fibronectin type III domain 5 (FNDC5), and resveratrol can both increase SIRT1, thereby reducing p53 K382 acetylation. This promotes p53 degradation and decreases p53 protein levels, which in turn upregulates SLC7A11 expression to inhibit ferroptosis [212, 213]. The absence of SIRT3 in cardiac cells similarly increases p53 acetylation, downregulates SLC7A11 expression, and enhances sensitivity to ferroptosis; replenishing SIRT3 can improve the ferroptosis process [214]. Class IIa histone deacetylases can also act on the p53 protein. Dysregulation of lipid metabolism can lead to lipotoxicity, which increases the expression of HDAC4 and HDAC5, thereby reducing the acetylation of p53 at the K120 site. This modification is essential for the transcriptional activation of apoptosis and ferroptosis [215]. Farnesoid X Receptor (FXR/NR1H4), as a scavenger for lipid peroxidation products, is involved in the regulation of ferroptosis. In adenocarcinoma tissues, both FXR and SLC7A11 are significantly upregulated. The underlying mechanism involves the competitive inhibition between FXR and CBP in the nucleus, which reduces the acetylation of p53 at lysine 382, thereby upregulating the expression of SLC7A11 [216]. In the pathophysiological process of acute lung injury, STAT6 is a key regulator of ferroptosis. STAT6 inhibits the acetylation of P53 by competing for binding with CBP and upregulates the expression of SLC7A11, thereby negatively regulating ferroptosis [217]. Snail is a transcription factor that belongs to the zinc finger protein family and is a key molecule in regulating the acetylation of p53. Its interaction with p53 and HDAC1 leads to the deacetylation of p53 at lysine 382. GINS4 is a promoting factor in the G1/S phase of the eukaryotic cell cycle. In lung adenocarcinoma, GINS4 activates Snail to inhibit the stability of p53, upregulating SLC7A11 to suppress ferroptosis, while this modification also provides an anti-apoptotic effect [218]. The above pathways all regulate ferroptosis through p53; however, we also observe that certain acetylation modifications of p53 exhibit anti-apoptotic properties. Therefore, it remains to be explored whether the ferroptosis induced by these modifications and the regulation of apoptosis are synergistic or competitive, as well as the extent to which different pathways modify p53 to regulate ferroptosis.

**Homeobox B9 (HOXB9):** HOXB9 is primarily involved in embryonic development and cell differentiation. In patients with lung cancer, cold atmospheric plasma (CAP) enhances the interaction between HOXB9 and the acetyltransferase P300/CBP-associated factor (PCAF), thereby increasing the level of K27 acetylation on HOXB9. Acetylated HOXB9 exhibits elevated ubiquitination, leading to its degradation and consequently diminishing its capacity, as a transcription factor, to regulate the expression of SLC7A11 [219] (Fig. 6B, Upper-middle).

**Lysophosphatidylcholine acyltransferase 2 (LPCAT2):** LPCAT2 belongs to the lysolipid acyltransferase family and plays a crucial role in regulating phospholipid metabolism and providing substrates for lipid peroxidation, with reported involvement in ferroptosis. In colorectal cancer, LPCAT2 regulates the acetylation of Protein Arginine Methyltransferase 1 (PRMT1) at the K145 site, preventing the cytoplasmic transport of PRMT1 in colorectal cancer cells. This leads to the loss of PRMT1’s role in the asymmetric dimethylation of histone H4 arginine 3 on LC7A11, thereby limiting the transcriptional activation of LC7A11 [220] (Fig. 6C).

**Gastric carcinoma-associated protein 41 (GAS41):** GAS41 is a histone-recognizing factor and oncogene that contains a YEATS domain and is involved in the regulation of ferroptosis. The mechanism involves the acetylation of lysine 27 on histone H3 by LC7A11 as a marker that can recruit GAS41. GAS41 activates the expression of LC7A11 by bridging the antioxidant response element NRF2 with the H3K27-acetylated site [221] (Fig. 6C, Bottom left).

**NAT10:** NAT10 is the only known enzyme that can mediate the N4-acetylcytidine (ac4C) modification of mRNA, which is crucial for the stability and translational efficiency of mRNA. In ferroptosis, it regulates the expression of the iron death-related protein cystine-glutamate transporter, SLC7A11, through its action on ac4C RNA modifications. Studies have found that NAT10 inhibits ferroptosis by stabilizing the SLC7A11 mRNA transcript [222] (Fig. 6C, Bottom left).

**Hydroxymethylglutaryl-CoA lyase (HMGCL):** HMGCL is a key enzyme in ketone body synthesis. In cancer patients with metabolic disorders, HMGCL regulates H3K9 acetylation through β-OHB and modulates the expression of dipeptidyl peptidase-4 (DPP4) in a dose-dependent manner, leading to ferroptosis in liver cancer cells [223]. Research indicates that DPP4 regulates ferroptosis by controlling the protein levels of SLC7A11 [224] (Fig. 6D, Lower-middle).

##### Acetylation modification of the GPX4 gene expression

GPX4 is an important antioxidant enzyme that plays a significant role in the inhibition of ferroptosis. High Mobility Group AT-hook 2 (HMGA2) is a critical transcription factor that, in pancreatic cancer patients, binds to and promotes modifications of the cis-regulatory elements in the GPX4 gene promoter region, enhancing enhancer activity by increasing H3K4 methylation and H3K27 acetylation [225] (Fig. 6E, Middle).

##### Acetylation modification of the FSP1 gene expression

FSP1 is an important protein that inhibits ferroptosis, capable of reducing coenzyme Q10 to its active form through its enzymatic activity, thereby eliminating intracellular lipid peroxides. Consistent with the stabilization of SLC7A11 mRNA transcripts, NAT10 modifies cytidine at position 132 of FSP1 mRNA via ac4C, stabilizing the FSP1 mRNA transcript to suppress ferroptosis [226] (Fig. 6F, Lower-middle).

##### Acetylation modifications of the FTH1 gene expression

Ferritin Heavy Chain 1 (FTH1) is a key component of ferritin, which isolates redox-active iron to inhibit the occurrence of ferroptosis. Although FTH1 is considered a suppressor of ferroptosis, its overexpression can induce iron autophagy and promote ferroptosis [227].

**Ferroptosis and epithelial cell apoptosis regulator (FEACR):** FEACR is a circRNA associated with ferroptosis. In cardiac cells after ischemia-reperfusion, FEACR binds to Nicotinamide Phosphoribosyltransferase (NAMPT), enhancing the stability of the latter. NAMPT increases NAD^+^ synthesis, thereby promoting Sirtuin 1 expression. Sirtuin 1 reduces the acetylation levels of FOXO1 at K242, K245, and K262. Hypoacetylation at these sites weakens its DNA-binding capacity and suppresses FOXO1 transcriptional activity, consequently decreasing the expression of downstream target genes (such as FTH1). The latter upregulates FTH1 transcription and inhibits ferroptosis [228] (Fig. 6G, Middle).

**Interleukin-1 beta (IL-1β):** IL-1βis a cytokine that, under inflammatory conditions, causes acetylation of nicotinamide nucleotide transhydrogenase (NNT) at the lysine (K) 1042 position, inducing the translocation of PCAF to the mitochondria. The acetylation of NNT at K1042 increases the binding affinity of NNT for NADP^+^, promoting the production of NADPH and maintaining the stability of iron-sulfur clusters (ISCs). The stability of ISCs is sensed by iron regulatory protein 1 (IRP1), which inhibits the expression of TFRC and promotes the transcription of FTH1, thereby maintaining iron homeostasis and inhibiting ferroptosis [229].

##### Acetylation modifications of the STAT3 gene expression

**Signal transducer and activator of transcription 3 (STAT3)**: STAT3 is an important transcription factor involved in various cell signaling pathways. In glioma, KAT6B facilitates the acetylation of histone H3 at lysine 23 and the enrichment of RNA polymerase II at the STAT3 promoter in glioma cells, thereby promoting STAT3 expression and exerting an inhibitory effect on ferroptosis [230]. In gastric cancer patients, STAT3 can bind to the consensus DNA response elements within the promoters of NR-related genes (GPX4, SLC7A11, and FTH1) and promote the expression of these ferroptosis-negative regulators, thereby establishing a negative STAT3–ferroptosis regulatory axis [231] (Fig. 6H, Bottom left).

#### Acetylation modifications regulating the expression of ferroptosis-related proteins

##### Acetylation modifications of the SLC7A11 protein expression

**Tubulin oligomerization and folding regulator (TUBORF):** TUBORF is a 92-amino acid peptide identified in cervical cancer, encoded by the lncRNA TUBA3FP. In cervical cancer, human papillomavirus (HPV) oncogenes E6 and E7 upregulate TUBORF through p300-mediated acetylation of histone H3 at lysine 27 (H3K27ac) on one hand; on the other hand, recruitment of Establishment of Sister Chromatid Cohesion N-Acetyltransferase 1 (ESCO1) enables its binding to TUBORF, leading to acetylation of TUBORF at K10 and K16. The latter promotes degradation of Immunity-related GTPase Q (IRGQ) via the ubiquitin–proteasome pathway. Inhibition of IRGQ upregulates the expression of SLC7A11 and GPX4 proteins, ultimately enhancing resistance to ferroptosis [232] (Fig. 6I, Top left).

##### Acetylation modifications of the GPX4 protein expression

**Heat shock protein family A member 5 (HSPA5):** HSPA5 is a molecular chaperone primarily expressed in the endoplasmic reticulum. In pancreatic ductal adenocarcinoma, HSPA5 mediates resistance to ferroptosis by maintaining the stability of GPX4 protein. The E1A-binding protein P300 (Enhancer of Zeste Homolog 2, EP300) acetyltransferase promotes ferroptosis through the acetylation of the K353 site on HSPA5, while HDAC6 limits the acetylation of HSPA5 and the subsequent ferroptosis [233].

**SIRT3**: is directly involved in the acetylation modification of GPX4. In cadmium-induced acute kidney injury, the expression of SIRT3 declines, leading to a high level of acetylation modification of GPX4. This modification results in a reduction of GPX4 protein levels, promoting ferroptosis [234].

##### Acetylation modifications of the FSP1 protein expression

**Solute Carrier Family 25 Member 1 (SLC25A1):** SLC25A1 is consistent with the xc-cystine-glutamate antiporter (SLC3A2 and SLC7A11) and belongs to the solute carrier superfamily. Recent studies have identified it as a key regulator of ferroptosis in human cancer cells. In cancer cells, SLC25A1 drives the translocation of citrate from the mitochondria to the cytoplasm and provides substrate for ATP citrate lyase (ACLY) to generate acetyl-CoA. Together with KAT2B, ACLY maintains FSP1 acetylation at K168, preventing its ubiquitination and degradation, while HDAC3 reverses this process [235].

##### Acetylation modifications of the DHODH protein expression

**Dihydroorotate dehydrogenase (DHODH):** DHODH is a key enzyme involved in the biosynthesis of pyrimidine nucleotides and acts as an oxidoreductase, catalyzing the conversion of dihydroorotic acid to orotic acid while reducing CoQ to CoQH2. CoQH2, as a potent antioxidant, effectively eliminates intracellular free radicals and lipid peroxides, thereby inhibiting the occurrence of ferroptosis. In cisplatin-induced acute kidney injury, mitochondrial SIRT3 decreases, resulting in increased acetylation levels of DHODH. Elevated acetylation levels of DHODH hinder pyrimidine biosynthesis and the generation of CoQH2, consequently promoting ferroptosis [236].

##### Acetylation modifications of the SDH protein expression

**Succinate dehydrogenase (SDH):** SDH, as part of the respiratory chain complex II, transfers electrons from succinate to coenzyme Q, participating in the oxidative phosphorylation process of the cell while catalyzing the oxidation of succinate to fumarate, releasing energy. Under ischemia-reperfusion injury, during the ischemic phase, SDH catalyzes in reverse, leading to succinate accumulation; during reperfusion, SDH oxidizes succinate in the forward direction, driving complex I to produce ROS [237]. TP53-Inducible Glycolysis and Apoptosis Regulator (TIGAR) inhibits glycolysis, increases the Pentose Phosphate Pathway (PPP), and promotes NADPH production to generate reduced glutathione for the clearance of reactive oxygen species (ROS). In ischemic stroke, TIGAR translocates to the mitochondria, enhancing the interaction between SIRT5 and SDH A while reducing the interaction between SIRT3 and SDH A. This leads to the acetylation and desuccinylation of SDH A, thereby inhibiting SDH activity. The latter reduces ROS production and suppresses ferroptosis [238].

##### Acetylation modifications of the ALOX12 protein expression

**Arachidonate 12-lipoxygenase (ALOX12):** ALOX12 belongs to the lipoxygenase family and primarily participates in fatty acid metabolism and the synthesis of bioactive lipids. It is a key protein that initiates the oxidation of membrane phospholipids. When Ferroptosis agonist RSL3 induces ferroptosis, it promotes the protein expression and acetylation of ALOX12. The exogenously applied NaHS, simulating hydrogen sulfide as a gas messenger, regulates oxidative stress and lipid metabolism. It has been found that NaHS alleviates the acetylation of ALOX12 and protects membrane lipids from peroxidation, thereby inhibiting ferroptosis [239].

A summary of acetylation modifications regulating the expression of ferroptosis-related genes/proteins is provided in Table 4.

**Table 4:** Acetylation modifications regulating ferroptosis.

Gene/Protein name	Acetylation modification sites	Acetylation regulatory mechanisms	Biological function (with reference numbers)
**p53**	K382, K120, K117, K161, K162, K98, K136	p53 3KR/3KR (K117,161,162 mutations) activates ferroptosis; adding K98 mutation eliminates pro-ferroptotic effect; adding K136 mutation abolishes tumor suppression.	Acetylation state downregulates SLC7A11 expression, thereby **promoting ferroptosis** [212–218].
**PRMT1**	K145	LPCAT2 increases the acetylation of PRMT1 at the K145 site	A hyperacetylated state leads to the loss of PRMT1-mediated asymmetric dimethylation of histone H4 arginine 3 at the LC7A11 locus, constraining LC7A11 transcriptional activation and **promoting ferroptosis** [220].
**STAT3**	Sites not specified	Acetylation enhances transcriptional activity.	Acetylated STAT3 binds to consensus DNA response elements in promoters of GPX4, SLC7A11, and FTH1, **inhibiting ferroptosis** [231].
**HOXB9**	K27	The interaction between HOXB9 and the acetyltransferase p300/CBP-associated factor enhances the K27 acetylation level of HOXB9.	Acetylation of HOXB9 promotes its **degradation,** **weakening** HOXB9’s ability as a transcription factor to regulate **SLC7A11 expression** [219].
**FSP1**	K168	SLC25A1/ACLY/KAT2B pathway maintains acetylation, preventing ubiquitination degradation. HDAC3 reverses this.	Acetylation stabilizes FSP1 protein, enhancing its reduction of CoQ10 and vitamin K, **inhibiting ferroptosis** [235].
**DHODH**	Sites not specified	Decreased SIRT3 leads to increased acetylation levels.	High acetylation hinders pyrimidine biosynthesis and CoQH2 generation, **promoting ferroptosis** [236].
**GPX4**	Sites not specified	Decreased SIRT3 results in hyperacetylation and reduced protein levels.	Hyperacetylation decreases GPX4 protein levels, **promoting ferroptosis** [234].
**HSPA5**	K353	EP300 catalyzes acetylation, promoting ferroptosis; HDAC6 limits acetylation.	Acetylation impairs HSPA5’s ability to stabilize GPX4 protein, **promoting ferroptosis** [233].
**TUBORF**	K10, K16	ESCO1 associates with TUBORF, catalyzing acetylation of TUBORF at K10 and K16.	TUBORF suppresses the upregulation of SLC7A11 and GPX4 protein expression, **enhancing resistance to ferroptosis** [232].
**ALOX12**	Sites not specified	NaHS (hydrogen sulfide donor) alleviates its acetylation.	Acetylation promotes its function in lipid peroxidation. Reducing acetylation protects membrane lipids, **inhibiting ferroptosis** [239].
**SDHA**	Sites not specified	TIGAR enhances SIRT5 interaction and reduces SIRT3 interaction, leading to acetylation.	Acetylation inhibits SDH enzyme activity, reducing ROS production and **inhibiting ferroptosis** [238].
**NNT**	K1042	IL-1β induces acetylation of NNT at the K1042 site.	NNT K1042 acetylation preserves **iron-sulfur clusters**, which **suppress TFRC expression and promote FTH1 transcription**, thereby maintaining iron homeostasis and **inhibiting ferroptosis** [229].
**SLC7A11 (gene)**	H3K27 at promoter	H3K27ac serves as a mark recruiting GAS41, activating expression.	Histone H3K27 acetylation **promotes SLC7A11 transcription,** **inhibiting ferroptosis** [221].
**TUBORF (gene)**	H3K27	p300-mediated acetylation of histone H3 lysine 27	TUBORF suppresses the upregulation of SLC7A11 and GPX4 protein expression, **enhancing resistance to ferroptosis** [232].
**GPX4 (gene)**	Promoter region	HMGA2 binds the promoter, enhancing enhancer activity via increased H3K4 methylation and H3K27 acetylation.	Histone modifications (including H3K27ac) at the promoter **promote GPX4 transcription,** **inhibiting ferroptosis** [225].
**FTH1 (gene)**	Promoter region	Sirt1 reduces FOXO1 acetylation, weakening DNA binding, thus suppressing FOXO1-mediated inhibition of FTH1 transcription (indirect promotion).	Low FOXO1 acetylation **upregulates FTH1 transcription,** **inhibiting ferroptosis** [228].
**DPP4 (gene)**	H3K9	HMGCL regulates H3K9 acetylation via β-OHB	DPP4 **regulates ferroptosis** by modulating **SLC7A11 protein levels** [223].
**FSP1 (mRNA)**	Cytidine 132 site (ac4C modification)	NAT10 mediates ac4C modification, stabilizing the mRNA transcript.	mRNA ac4C acetylation **enhances FSP1 mRNA stability,** **inhibiting ferroptosis** [226].
**SLC7A11 (mRNA)**	Sites not specified	NAT10 stabilizes mRNA transcript via ac4C modification.	mRNA ac4C acetylation **enhances SLC7A11 mRNA stability,** **inhibiting ferroptosis** [222].

*p53* tumor protein p53, *PRMT1* protein arginine methyltransferase 1, *STAT3* signal transducer and activator of transcription 3, *HOXB9* homeobox B9, *FSP1* ferroptosis suppressor protein 1, *DHODH* dihydroorotate dehydrogenase, *GPX4* glutathione peroxidase 4, *HSPA5* heat shock protein family A (Hsp70) member 5, *TUBORF* tubulin oligomerization and folding regulator, *ALOX12* arachidonate 12-lipoxygenase, *SDHA* succinate dehydrogenase complex flavoprotein subunit A, *NNT* nicotinamide nucleotide transhydrogenase, *SLC7A11* solute carrier family 7 member 11, *FTH1* ferritin heavy chain 1, *DPP4* dipeptidyl peptidase-4, *NAT10* N-acetyltransferase 10, *EP300* E1A-binding protein P300, *HDAC3* histone deacetylase 3, *SIRT3* sirtuin 3, *ESCO1* establishment of sister chromatid cohesion N-acetyltransferase 1, *TIGAR* TP53 induced glycolysis regulatory phosphatase, *SIRT5* sirtuin 5, *GAS41* glioma-amplified sequence 41, *HMGA2* high mobility group AT-hook 2, *FOXO1* forkhead box O1, *HMGCL* 3-hydroxy-3-methylglutaryl-CoA lyase, *β-OHB* beta-hydroxybutyrate.

### Acetylation-mediated pyroptosis

Acetylation-mediated cell pyroptosis primarily regulates the expression of genes associated with pyroptosis through acetylation modifications. These modifications facilitate the functional activation of key proteins involved in pyroptosis, thereby promoting or inhibiting the occurrence of pyroptosis.

#### Acetylation modification of genes related to the regulation of cellular senescence

##### Acetylation modifications of the NF-κB gene expression

Nuclear Factor-κB (NF-κB) is an important family of transcription factors, including RelA, RelB, c-Rel, p50, and p52, which are widely involved in regulating immune-inflammatory responses, cell survival, and proliferation [240]. When cells are stimulated by inflammatory signals (such as TNF-α and LPS) or stress signals, IκB is phosphorylated and degraded, releasing NF-κB into the nucleus, where it activates the expression of various downstream inflammation and immune-related genes [241]. It can upregulate the expression of the NLRP3 inflammasome and its associated molecules, such as NLRP3, caspase-1, and pro-IL-1β, laying a molecular foundation for the occurrence of pyroptosis [242].

**Fig. 7:**
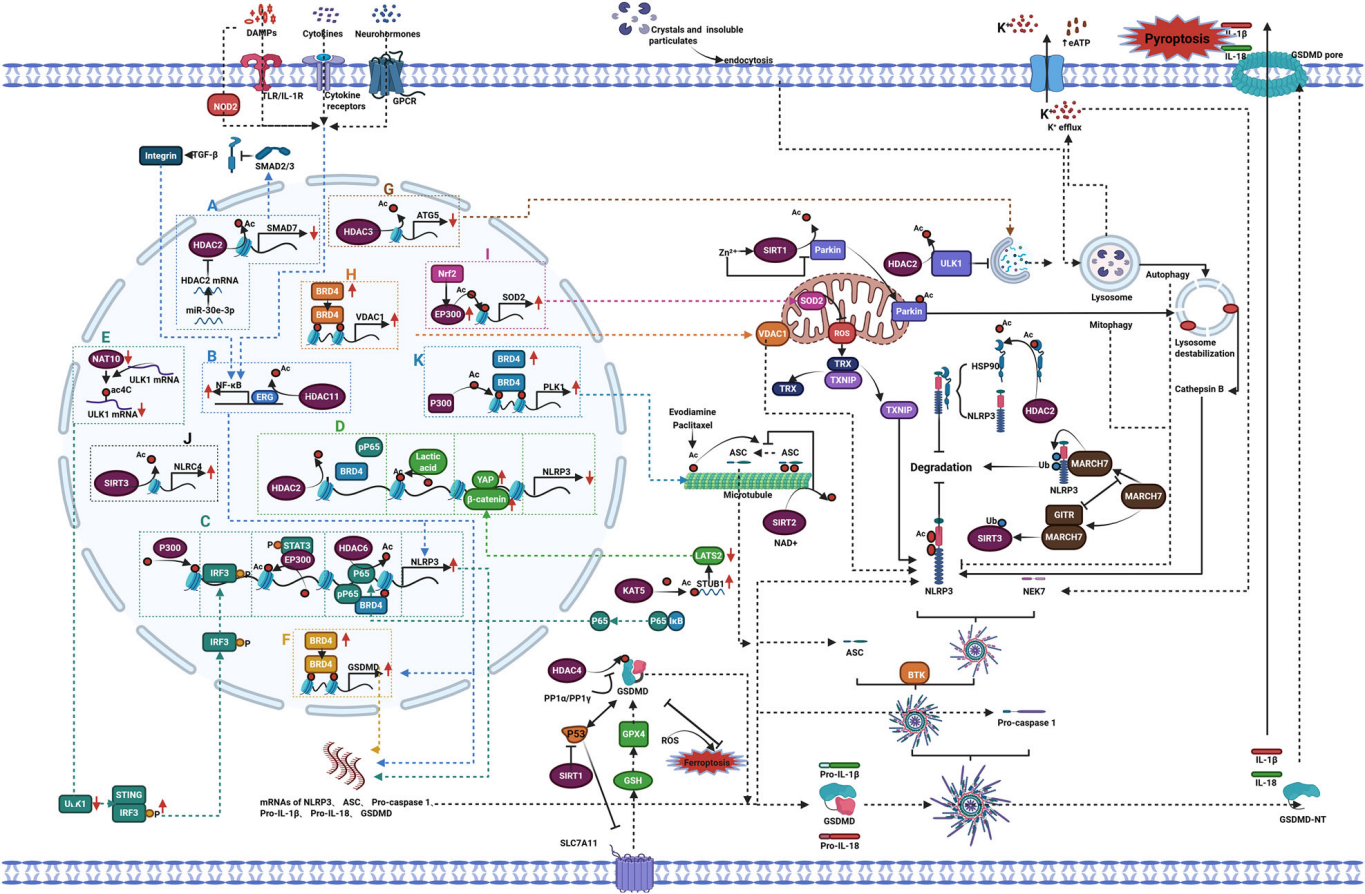
Schematic of acetylation regulation in regulated pyroptosis. **A** HDAC2 suppresses the expression of SMAD7 by catalyzing the deacetylation of H3K27 at the SMAD7 promoter; meanwhile, miR-30e-3p promotes the expression of SMAD7 by inhibiting HDAC2 expression through binding with HDAC2 mRNA, thus inhibiting TGF-β-mediated NF-κB activation and exerting an anti-necrotic effect. **B** ERG concurrently inhibits TNF-α-dependent activation of the ICAM-1 promoter and NF-κB activity; however, HDAC11 may activate NF-κB and promote pyroptosis by regulating the acetylation level of ERG. **C** p65 typically forms a heterodimer with p50 and remains in the cytoplasm by binding to the inhibitor protein IκB. When cells are stimulated (such as by inflammatory factors or pathogens), IκB is degraded, and p65/p50 translocates to the nucleus, inducing multiple target genes, including those acting on the NLRP3 promoter region. Acetylation of p65 can inhibit the expression of NLRP3, while HDAC6 decreases the acetylation level of p65, promoting pyroptosis; phosphorylated STAT3 recruits EP300 to form a complex that enhances acetylation of histones H3 and H4 at the NLRP3 promoter, leading to cellular pyroptosis, which can be inhibited by colchicine; in addition, P300 directly increases histone acetylation levels at the NLRP3 promoter region, resulting in high expression of NLRP3 and subsequent pyroptosis; overexpression of HDAC2 suppresses the recruitment of the BRD4-p-p65 complex mediated by H3K27ac, thus inhibiting NLRP3 transcription and the occurrence of pyroptosis. **D** KAT5 promotes STUB1 transcription through acetylation, leading to the ubiquitination and degradation of LATS2 and activation of the YAP/β-catenin pathway. The interaction of YAP and β-catenin in the nucleus can inactive NLRP3 and inhibit pyroptosis; lactate can promote histone H3K9 acetylation and H3K18 lactylation, inhibiting NLRP3 expression and consequently preventing pyroptosis. **E** The reduction in NAT10 expression leads to a decrease in ULK1 transcripts, thereby reducing ULK1 levels. As a regulator of STING phosphorylation, the absence of ULK1 enhances the activation of the STING-IRF3 signaling pathway, resulting in the nuclear translocation of IRF3 and increased expression of NLRP3. **F** BRD4 is upregulated during hepatocyte lipotoxicity, subsequently regulating H3K27 acetylation in the GSDMD gene promoter region, thereby enhancing GSDMD expression. **G** Increased expression of HDAC3 results in decreased H3K27 acetylation of ATG5, which inhibits the expression of ATG5. The HDAC3 inhibitor BRD3308 can upregulate ATG5 expression through a deacetylation effect, which reduces cellular reactive oxygen species accumulation and inhibits pyroptosis. **H** Elevated levels of BRD4 further regulate H3K27 acetylation in the VDAC gene promoter region, thereby enhancing VDAC expression and recruiting NLRP3 to promote pyroptosis. **I** Nrf2 can recruit EP300, thereby improving H3K27 acetylation modification of SOD2, resisting oxidative stress, and inhibiting pyroptosis. **J** SIRT3-mediated deacetylation of NLRC4 at k71 and K272 promotes its activation, which facilitates inflammasome formation and subsequent pyroptosis. **K** Elevated levels of BRD4 recognize P300-dependent H3K27 acetylation, promoting the expression of the PLK1 gene promoter, strengthening microtubule-organizing center structures, and regulating the subcellular localization of NLRP3 to activate the inflammasome and subsequent pyroptosis. Mitochondrial autophagy also plays an important role in the acetylation regulation of pyroptosis. The PINK1-Parkin-mediated mitophagy can reduce mitochondrial ROS and subsequent activation of the NLRP3 inflammasome. Exogenous Zn²⁺ binds to SIRT1 and significantly inhibits its activity, leading to the upregulation of Parkin acetylation, which promotes mitochondrial autophagy and inhibits NLRP3 inflammasome activation and cellular pyroptosis. During the formation of NLRP3, HSP90 acts as a non-histone substrate of HDAC6, enhancing protein binding capabilities after deacetylation, and can bind to the NLRP3 protein to prevent its degradation and subsequent formation of the NLRP3 inflammasome. GITR competes with NLRP3 for binding to the E3 ubiquitin ligase MARCH7 and recruits MARCH7 to induce the degradation of SIRT2, resulting in decreased ubiquitination of NLRP3 but increased acetylation. This process reduces the degradation of NLRP3 and promotes subsequent pyroptosis. Elevated HDAC2 facilitates the deacetylation of ULK1 at K68, weakening ULK1-mediated autophagy; thus, degradation of NLRP3 is inhibited, driving the occurrence of pyroptosis. Acetylated α-tubulin promotes the dynein-mediated transport of mitochondria (a transport carrier for the NLRP3 inflammasome adapter ASC) along microtubules to the negative end (i.e., the perinuclear region), enhancing the proximity between ASC and NLRP3 on the endoplasmic reticulum and promoting the assembly of the NLRP3 inflammasome. Conversely, the SIRT2 agonist resveratrol and NAD^+^ inhibit the acetylation of α-tubulin, thereby suppressing ASC-mediated assembly and activation of the NLRP3 inflammasome. The regulation of GSDMD protein acetylation involves the p53/GPX4/GSDMD axis, with GSDMD exerting a positive feedback effect on p53. The activation of SIRT1 inhibits the p53/GPX4/GSDMD axis by inducing p53 acetylation, thereby alleviating damage caused by pyroptosis. HDAC4 is responsible for mediating the deacetylation of GSDMD, while the PP1 catalytic subunits PP1α and PP1γ dephosphorylate and inactivate HDAC4, thus promoting acetylation-mediated pyroptosis of GSDMD. HDAC2 histone deacetylase 2, SMAD7 SMAD family member 7, TGF-β transforming growth factor beta, NF-κB nuclear factor kappa B, Erg ETS-related gene (The ETS family comprises a class of transcription factors that recognize and bind to a specific DNA sequence in the promoter regions of target genes, thereby activating or repressing their transcription. Dysregulation of these factors can convert them into potent key oncogenic regulators.), TNF-α tumor pyroptosis factor alpha, ICAM-1 intercellular adhesion molecule 1, HDAC11 histone deacetylase 11, p65 P65 subunit of NF-κB, p50 P50 subunit of NF-κB, IκB inhibitor of kappa B, NLRP3 NOD-like receptor protein 3, HDAC6 histone deacetylase 6, STAT3 signal transducer and activator of transcription 3, EP300 E1A-binding protein p300, P300 E1A-binding protein p300, BRD4 bromodomain-containing 4, KAT5 lysine acetyltransferase 5, STUB1 STIP1 homology and U-box containing protein 1, LATS2 large tumor suppressor kinase 2, NAT10 N-acetyltransferase 10, ULK1 Unc-51 like autophagy-activating kinase 1, STING stimulator of interferon genes, IRF3 interferon regulatory factor 3, GSDMD gasdermin D, HDAC3 histone deacetylase 3, ATG5 ATPase associated with diverse cellular activities 5, VDAC voltage-dependent anion channel, Nrf2 nuclear factor erythroid 2-related factor 2, SOD2 superoxide dismutase 2, SIRT3 sirtuin 3, NLRC4 NLR family CARD domain-containing 4, PLK1 polo-like kinase 1, PINK1-Parkin PTEN-induced kinase 1 and parkin, SIRT1 sirtuin 1, HSP90 heat shock protein 90, HDAC6 histone deacetylase 6, GITR glucocorticoid-induced TNFR family related protein, SIRT2 sirtuin 2, ASC apoptosis-associated speck-like protein containing a CARD, GPX4 glutathione peroxidase 4, HDAC4 histone deacetylase 4, PP1 protein phosphatase 1, PP1α protein phosphatase 1 alpha, PP1γ protein phosphatase 1 gamma.

**SMAD family member 7 (SMAD7):** SMAD7 is an inhibitory SMAD protein in the classical TGF-β signaling pathway that suppresses TGF-β signaling by negatively regulating the phosphorylation and transcriptional activity of SMAD2/3 [243]. Moreover, TGF-β can promote the activation of NF-κB through integrin signaling [244]. Research on cardiomyocyte pyroptosis and inflammation induced by coronary microembolism has found that HDAC2 inhibits the expression of SMAD7 by catalyzing the deacetylation of H3K27 at the SMAD7 promoter. Meanwhile, miR-30e-3p promotes the expression of SMAD7 by binding to HDAC2 mRNA and thus inhibiting the expression of HDAC2, which in turn suppresses TGF-β-mediated activation of NF-κB, exerting an anti-apoptotic effect [245] (Fig. 7A, Top left).

**ETS-related gene (ERG) :** ERG is a transcription factor of the ETS family that can bind to specific sequences and regulate the transcription of downstream genes. It plays an important role in processes such as embryonic development, angiogenesis, hematopoiesis, and tumorigenesis [246]. Research has found that ERG simultaneously inhibits TNF-α-dependent activation of the ICAM-1 promoter and NF-κB activity [247]; however, HDAC11 may activate NF-κB by regulating the acetylation levels of ERG, promoting the occurrence of pyroptosis [248]. (Fig. 7B, Left center)

##### Acetylation modifications of the NLRP3 gene expression

**p65:** p65 is a key subunit of the classical NF-κB family, and in inflammation and immune regulation, p65 typically forms heterodimers with p50 and remains in the cytoplasm bound to the inhibitory protein IκB. When cells are stimulated (such as by inflammatory factors or pathogens), IκB is degraded, allowing p65/p50 to enter the nucleus and induce multiple target genes, including enhancing the transcription of NLRP3 by acting on its promoter region [249]. In atherosclerosis, the acetylation of p65 can inhibit the expression of NLRP3, while HDAC6 reduces the acetylation levels of p65, promoting the occurrence of pyroptosis [250] (Fig. 7C, Bottom left).

**The C-terminus of Hsc70 interacting protein (STUB1/CHIP):** STUB1 is a protein with E3 ubiquitin ligase activity. It primarily participates in the processes of protein folding and degradation by binding to molecular chaperones such as Hsp70 and Hsp90. This protein tags misfolded or aggregated proteins within cells with ubiquitin and directs their degradation [251]. In myocardial ischemia-reperfusion injury, KAT5 enhances the transcription of STUB1 through acetylation, which subsequently leads to the ubiquitination and degradation of LATS2 (a cascade kinase in the Hippo pathway that inactivates YAP through phosphorylation), thereby activating the YAP/β-catenin pathway [252]. The interaction between YAP and β-catenin in the cell nucleus can inactivate NLRP3 and inhibit the occurrence of pyroptosis [253] (Fig. 7D, Left center).

**STAT3:** STAT3 is also involved in the regulation of pyroptosis. In acute lung injury caused by sepsis, phosphorylated STAT3 recruits EP300 to form a complex that promotes the acetylation of histones H3 and H4 at the NLRP3 promoter, leading to cellular pyroptosis. Colchicine can inhibit this process [254] (Fig. 7C, Bottom left).

**Stimulator of interferon genes (STING):** STING is an important intracellular signaling protein that plays a role in antiviral immunity, inflammatory responses, and tumor immunity. It activates downstream Interferon Regulatory Factor 3 (IRF3) by sensing intracellular DNA, particularly pathogen-derived DNA, which in turn triggers the expression of interferons and inflammatory factors [255]. Research has found that lipopolysaccharide stimulation triggers the translocation of STING to the perinuclear region, which further binds to and phosphorylates IRF3. The phosphorylated IRF3 translocates into the nucleus, enhancing the expression of NLRP3 [256]. In sepsis, reduced NAT10 expression lowers the ac4C modification level of ULK1 mRNA, leading to decreased ULK1 mRNA stability and increased degradation. As a regulatory factor for STING phosphorylation, the absence of ULK1 enhances the activation of the STING-IRF3 signaling pathway, subsequently resulting in an increase in the pro-pyroptotic NLRP3 inflammasome in neutrophils [257] (Fig. 7E, Left Center).

**Lactic acid** is primarily produced through glycolysis under anaerobic or high-intensity exercise conditions, and is involved in functions such as pH regulation and signal transduction. In ulcerative colitis, lactic acid can promote histone H3K9 acetylation and H3K18 lactylation while inhibiting NLRP3 expression, thereby suppressing pyroptosis [258] (Fig. 7D, Left center).

In addition, certain acetyltransferases and deacetylases can directly modify histones on NLRP3, regulating subsequent pyroptosis. In a study on decreased articular cartilage quality in female offspring rats induced by maternal nicotine exposure, P300 was found to increase H3K9 and H3K14 acetylation levels at the NLRP3 promoter region, leading to elevated NLRP3 expression and subsequent pyroptosis [259]. In colorectal cancer, the high expression of HDAC2 inhibits the recruitment of the BRD4-p-P65 complex mediated by H3K27ac, thereby suppressing the transcription of NLRP3 and the occurrence of pyroptosis [260] (Fig. 7C, Bottom left).

##### Acetylation modifications of the GSDMD gene expression

GSDMD is an executor of pyroptosis, and currently, there are only a few reports on its gene expression level regulation through acetylation. In metabolic dysfunction-associated fatty liver disease, BRD4 is upregulated during hepatocyte lipotoxicity, thereby regulating the H3K27 acetylation in the promoter region of the GSDMD gene, which enhances its expression [261] (Fig. 7F, Lower left).

##### Acetylation modifications that influence the expression of other pyroptosis-related genes

**ATPase associated with diverse cellular activities 5 (AGT5):** AGT5 is a protein encoded by human ATPase-related genes. It belongs to the ATPase-related protein family and is typically associated with various essential intracellular processes, including the regulation of autophagy, cell cycle control, transcription, and protein folding and transport [262]. In acute lung injury caused by sepsis, the increased expression of HDAC3 leads to decreased acetylation of H3K27 on ATG5, inhibiting its expression. The HDAC3 inhibitor BRD3308 upregulates ATG5 expression by inhibiting deacetylation, which reduces the accumulation of reactive oxygen species and suppresses pyroptosis [263] (Fig. 7G, Top left).

**VDAC:** VDAC is a multifunctional protein located in the outer membrane of mitochondria and belongs to the family of voltage-dependent anion channels. It plays a crucial role in cellular energy metabolism, as well as the transport of ions and small molecules. Research has revealed that the oligomerization of VDAC recruits NLRP3, enabling its close association with VDAC and subsequently promoting the activation of the inflammasome [264]. During hepatic cellular lipotoxicity, BRD4 levels increase, thereby modulating the H3K27 acetylation of the VDAC gene promoter region, which enhances VDAC expression and recruits NLRP3 to promote pyroptosis [261] (Fig. 7H, Top left).

**Manganese superoxide dismutase 2 (SOD2):** SOD2 is an important antioxidant enzyme that primarily functions in the mitochondria of cells. It helps cells combat oxidative stress by converting harmful superoxide anions (O2–) into hydrogen peroxide and oxygen. When cells are under stress, the activation of Nrf2 can rapidly increase SOD2 levels, assisting in the removal of excess superoxide anions and alleviating oxidative damage [265]. In cancer, Nrf2 can recruit the E1A-binding protein p300 (EP300), thereby enhancing the H3K27 acetylation modification of SOD2, which counters oxidative stress and inhibits ferroptosis [266] (Fig. 7I, Upper-middle).

**NOD-like receptor family, CARD domain-containing protein 4 (NLRC4):** NLRC4 is a protein that belongs to the NLR (NOD-like receptors) family. It primarily activates inflammatory responses by forming inflammasomes, distinguishing itself from NLRP3 by mainly recognizing specific molecules from bacteria, such as flagellin or plasmid proteins, to initiate activation [267]. Research has found that SIRT3-mediated deacetylation of NLRC4 at the K71 and K272 sites can promote its activation, which in turn facilitates the formation of the inflammasome and subsequent pyroptosis [268] (Fig. 7J, Left center).

**Polo-like kinase 1 (PLK1):** PLK1 is a serine/threonine protein kinase that plays a critical regulatory role in processes such as the transition from G₂ to M phase in eukaryotic cells, chromosome separation, and cell division. Through pharmacological and genetic methods, it has been discovered that PLK1 can promote the activation of the NLRP3 inflammasome during interphase. The mechanism involves PLK1 enhancing the structure of the microtubule-organizing center and regulating the subcellular localization of NLRP3, thereby activating the inflammasome and subsequent pyroptosis [269]. In diabetic nephropathy, the level of the epigenetic “reader” BRD4 is elevated, which recognizes P300-dependent H3K27 acetylation, promoting the expression of the PLK1 gene promoter, leading to the activation of the NLRP3 inflammasome and subsequent pyroptosis [270] (Fig. 7K, Middle).

#### Acetylation modification of proteins related to the regulation of pyroptosis

##### Acetylation modifications of the NLRP3 protein expression

**Parkin** is a protein encoded in the human body, belonging to the E3 ubiquitin ligase family. The gene is located on human chromosome 6 and is primarily involved in the ubiquitination process of intracellular proteins. It mediates the selective autophagy of damaged mitochondria by promoting the ubiquitination tagging of these damaged mitochondria for degradation via autophagosomes [271]. In acute kidney injury, PINK1-Parkin-mediated mitophagy can reduce mitochondrial ROS and subsequent NLRP3 inflammasome activation [272]. In sepsis-induced acute kidney injury, exogenous Zn²⁺ binds to SIRT1 and significantly inhibits its activity, which in turn upregulates Parkin acetylation to promote mitochondrial autophagy and suppress the activation of the NLRP3 inflammasome and cell pyroptosis [273].

**70** **kDa heat shock protein (HSP70) and 90** **kDa heat shock protein (HSP90):** HSP70 and HSP90 are both important molecular chaperones involved in protein folding, stabilization, and repair, helping cells respond to various stresses and maintain normal cellular functions. Research has found that HSP90, as a non-histone substrate of HDAC6, exhibits enhanced protein binding abilities after deacetylation and can bind to the NLRP3 protein to prevent its degradation and the subsequent formation of the NLRP3 inflammasome [274]. When using HSP90 inhibitors, the association between its co-chaperones and substrate proteins can be shifted to HSP70. Unlike HSP90, HSP70 promotes the proteasomal degradation of substrate proteins after binding to them [43]. In postoperative cognitive dysfunction, the expression of HDAC6 is increased, activating the NLRP3 inflammasome to induce pyroptosis in hippocampal microglia. The mechanism involves a decrease in HSP90 acetylation levels, which stabilizes NLRP3 and leads to subsequent pyroptosis. In contrast, the binding of HSP70 to NLRP3 triggers its degradation, although this effect is less significant than the protective role of HSP90 against NLRP3 degradation [275].

**Glucocorticoid-Induced TNFR family related protein (GITR):** GITR is a cell surface protein belonging to the tumor pyroptosis factor receptor family, which plays a significant role in immune regulation and inflammatory responses [276]. In sepsis, GITR enhances the uptake of lysolipid phosphates by macrophages and specifically amplifies macrophage pyroptosis mediated by the NLRP3 inflammasome. The mechanism involves GITR competing with NLRP3 for binding to the E3 ubiquitin ligase MARCH7, and recruiting MARCH7 to induce the degradation of the deacetylase SIRT2. This leads to a reduction in ubiquitination but an increase in acetylation of NLRP3, ultimately decreasing its degradation and promoting subsequent pyroptosis [277].

**UNC-51-like autophagy-activating kinase 1 (ULK1):** ULK1 is a serine/threonine kinase that belongs to the UNC-51 family and plays a critical role in the process of autophagy within cells, particularly in regulating the initiation phase of autophagy. Research has shown that autophagy can negatively regulate the activation of inflammatory bodies by clearing misfolded proteins and damaged organelles. As one of the autophagy genes, ULK1 promotes autophagy while inhibiting the activity of the NLRP3 inflammasome [278]. In acute liver failure, RNA sequencing results reveal that ULK1 is a negative regulatory target of HDAC2. When HDAC2 levels are elevated, the K68 deacetylation of ULK1 occurs, which diminishes ULK1-mediated autophagy and inhibits NLRP3 degradation, driving the onset of pyroptosis [279].

**Microtubules:** Microtubules are a crucial component of the cytoskeleton, composed of α- and β-tubulin dimers, forming tubular structures within the cell. They play an important role in the structure, dynamics, and function of the cell. Research indicates that microtubules are significant in regulating the activation of the NLRP3 inflammasome, and the acetylation of α-tubulin enhances the flexibility of microtubules, accelerating the movement of motor proteins [280]. Importantly, acetylated α-tubulin can promote the dynein-mediated transport of mitochondria (a transport carrier for the NLRP3 inflammasome adapter ASC) along microtubules toward the negative end (i.e., the perinuclear area), thereby enhancing the proximity between ASC and NLRP3 on the endoplasmic reticulum and facilitating the assembly of the NLRP3 inflammasome [281]. The SIRT2 agonists resveratrol (5 μM) and NAD^+^ (10 μM) specifically suppress acetylation at lysine 40 (K40) of α-tubulin—reducing the proportion of highly acetylated cells from >60% to ~20%—thereby inhibiting ASC-mediated assembly and activation of the NLRP3 inflammasome [282]. Studies have shown that evodiamine (which increases acetylation levels by approximately 3–4-fold at a concentration of 5 μM) and paclitaxel (which induces peak acetylation within 30 min at 33 nM) both enhance NLRP3 inflammasome activation and subsequent pyroptosis by specifically promoting acetylation at lysine 40 (K40) of α-tubulin—a process dependent on the acetyltransferase MEC-17—thereby revealing their mechanisms of action in cancer therapy [283, 284].

##### Acetylation modifications of the GSDMD protein expression

GSDMD protein is the executioner of pyroptosis, regulated by GPX4 protein. In acute liver failure, knocking out GSDMD reduces p53 expression while increasing GPX4 levels; silencing GPX4 significantly elevates markers of ferroptosis and GSDMD, indicating the existence of the p53/GPX4/GSDMD axis, with GSDMD exerting a positive feedback effect on p53 [285]. Similarly, in acute liver failure, the activation of SIRT1 induces the acetylation of p53, thereby inhibiting the p53/GPX4/GSDMD axis and alleviating damage caused by ferroptosis [12]. Research has found that GSDMD can undergo acetylation at lysine 248, and this acetylation enhances pyroptosis. HDAC4 is the specific deacetylase responsible for mediating the deacetylation of GSDMD, while the catalytic subunits of protein phosphatase 1 (PP1), namely PP1α and PP1γ, dephosphorylate and inactivate HDAC4, thereby promoting acetylation-mediated pyroptosis of GSDMD [286].

A summary of acetylation modifications regulating the expression of pyroptosis-related genes/proteins is presented in Table 5.

**Table 5:** Acetylation modifications regulating pyroptosis.

Gene/Protein name	Acetylation modification sites	Acetylation regulatory mechanisms	Biological function (with reference numbers)
**NLRP3 (gene)**	H3 (K9, K14, K27), H4	P300 increases H3K9 and H3K14 acetylation at the promoter; STAT3-EP300 promotes histone H3 and H4 acetylation; Lactate can promote histone H3K9 acetylation.	Histone hyperacetylation (H3K9ac, H3K14ac, H3K27ac, H4ac) at the promoter promotes NLRP3 gene transcription, foundational for **pyroptosis** [254, 258–260].
**p65**	Sites not specified	Its acetylation can inhibit NLRP3 expression; HDAC6 reduces its acetylation.	p65 acetylation **inhibits pyroptosis**; deacetylation **promotes pyroptosis** [250].
**GSDMD**	K248	HDAC4 mediates deacetylation; PP1 inactivates HDAC4, promoting acetylation.	Acetylation enhances GSDMD pore-forming function, **promoting pyroptosis** execution [286].
**NLRC4**	K71, K272	SIRT3 mediates deacetylation.	Deacetylation promotes its activation, thereby **promoting pyroptosis** [268].
**Parkin**	Sites not specified	Exogenous Zn²⁺ inhibits SIRT1 activity, upregulating its acetylation.	Acetylation promotes Parkin-mediated mitophagy (mitochondrial clearance), thereby **inhibiting NLRP3 inflammasome activation and pyroptosis** [273].
**HSP90**	Sites not specified	HDAC6 causes deacetylation.	Deacetylation enhances HSP90 binding to NLRP3 protein, preventing NLRP3 degradation, thereby **promoting pyroptosis** [43, 274, 275].
**ULK1**	K68	Increased HDAC2 leads to its deacetylation.	Deacetylation weakens ULK1-mediated autophagy (a cellular clearance mechanism), inhibiting NLRP3 degradation, thus **driving pyroptosis** [279].
**α-tubulin**	K40	SIRT2 agonists resveratrol (5 μM) and NAD^+^ (10 μM) reduce K40 acetylation from >60% to ~20%.	Acetylated α-tubulin promotes dynein-mediated transport of mitochondria (carrying ASC) to the perinuclear area, enhancing ASC proximity to NLRP3 on the ER, **promoting pyroptosis** [283, 284]. Deacetylation **inhibits pyroptosis**.
**GSDMD(gene)**	H3K27	BRD4 upregulates H3K27 acetylation at the GSDMD gene promoter region	Increased GSDMD expression **promotes pyroptosis** [261].
**SMAD7 (gene)**	H3K27	HDAC2 catalyzes the deacetylation of H3K27 at the SMAD7 promoter.	Deacetylation of SMAD7 suppresses its expression, thereby inhibiting TGF-β–mediated activation of NF-κB and exerting an **anti-pyroptotic effect** [245].
**ERG (gene)**	Sites not specified	HDAC11 may regulate the acetylation level of ERG	Low acetylation levels of ERG activate NF-κB and **promote pyroptosis** [248].
**ULK1 (mRNA)**	Sites not specified	Reduced NAT10 expression leads to decreased ac4C modification of ULK1 mRNA.	Loss of ULK1 enhances activation of the STING-IRF3 signaling pathway, thereby leading to **increased pro-pyroptotic N**LRP3 inflammasome in neutrophils [257].
**ATG5 (gene)**	H3K27	Increased HDAC3 expression reduces H3K27 acetylation at ATG5.	**The HDAC3 inhibitor** BRD3308 upregulates ATG5 expression by suppressing deacetylation, which in turn reduces the accumulation of reactive oxygen species and **inhibits pyroptosis** [263].
**VDAC (gene)**	H3K27	BRD4 Regulates H3K27 Acetylation at the VDAC Gene Promoter Region	Upregulation of VDAC and recruitment of NLRP3 **promote the occurrence of pyroptosis** [261].
**SOD2 (gene)**	H3K27	Nrf2 can recruit EP300, thereby mediating H3K27 acetylation of SOD2	SOD 2 in a hyperacetylated state resists oxidative stress and **suppresses pyroptosis** [266].
**PLK1 (gene)**	H3K27	Elevated BRD4 levels recognize P300-dependent H3K27 acetylation and promote the expression of the PLK1 gene promoter	PLK1 induces **activation** of the NLRP3 inflammasome and subsequent **pyroptosis** [270].

*NLRP3* NOD-like receptor family pyrin domain-containing 3, *p65* NF-kappa-B p65 subunit (RELA), *GSDMD* gasdermin D, *NLRC4* NLR family CARD domain-containing 4, *Parkin* parkin RBR E3 ubiquitin protein ligase, *HSP90* heat shock protein 90, *ULK1* Unc-51 like autophagy-activating kinase 1, *α-tubulin* alpha-tubulin, *SMAD7* SMAD family member 7, *ERG* ETS-related gene, *ATG5* autophagy related 5, *VDAC* voltage-dependent anion channel, *SOD2* superoxide dismutase 2, *PLK1* polo-like kinase 1, *P300* E1A-binding protein p300, HDAC6 histone deacetylase 6, *HDAC4* histone deacetylase 4, *PP1* protein phosphatase 1, *SIRT3* sirtuin 3, *SIRT1* sirtuin 1, *HDAC2* histone deacetylase 2, *SIRT2* sirtuin 2, *BRD4* bromodomain-containing protein 4, *HDAC11* histone deacetylase 11, *NAT10* N-acetyltransferase 10, *STING* stimulator of interferon genes, *IRF3* interferon regulatory factor 3, *HDAC3* histone deacetylase 3, *Nrf2* nuclear factor erythroid 2-related factor 2, *EP300* E1A-binding protein p300.

## Discussion

### Exploring the crosstalk mechanisms of apoptosis, ferroptosis, and pyroptosis

#### ROS is an important factor in the processes of apoptosis, ferroptosis, and pyroptosis

From the overview of apoptosis, ferroptosis, and pyroptosis in the text, we find that ROS play a significant role in the occurrence of these three types of cell death. In apoptosis [92], ROS act as one of the endogenous activating factors that initiate the process; in pyroptosis, the increase in ROS levels leads to the release of TXNIP from thioredoxin, promoting the activation of NLRP3 and subsequent inflammasome formation [191], while also enhancing the activation of caspase-1 and IL-1β [193, 194]; in ferroptosis, ROS is indispensable and constitutes a necessary condition for the occurrence of this type of cell death [105]. Therefore, a question arises: when the intracellular levels of ROS increase and exceed the compensatory limits of the cell, which of the three types of cell death will occur? The author believes that, in addition to the increase of ROS, a higher ratio of unsaturated fatty acids and an imbalance in intracellular iron homeostasis are two other crucial factors; the occurrence of ferroptosis requires the fulfillment of all three conditions. In terms of pyroptosis, the elevation of ROS levels does not necessarily lead to the formation of inflammasomes. Research has found that the activation of NLRP3 is triggered not only by ROS but also influenced by various other factors, such as ATP, intracellular calcium ion concentration, bacterial infections, and other danger signals. Therefore, the complete activation of NLRP3 generally requires the combined action of multiple signals [287]. Furthermore, numerous studies indicate that reactive oxygen species (ROS) may need to interact with other intracellular signals, such as inflammatory factors or changes in ion channels, to effectively induce the assembly and activation of the NLRP3 inflammasome [288]. Therefore, the increase in ROS (reactive oxygen species) is only a facilitating factor in the occurrence of apoptosis, rather than a decisive factor. In terms of apoptosis, an elevation in ROS beyond the compensatory capacity of the cell can induce apoptosis; however, the downstream regulatory mechanisms that mediate ROS-induced apoptosis will still influence whether apoptosis ultimately occurs [289]. Although ROS plays an important role in cell death, they are not the only factor or the final link leading to cell death. Thus, the mode of cell death that occurs is closely related to the type of cell and the environment in which the cell resides. For instance, the simultaneous presence of increased ROS levels, elevated proportions of unsaturated fatty acids, and an imbalance in intracellular iron homeostasis may lead to ferroptosis; whereas the coexistence of increased ROS levels and heightened inflammatory response may result in pyroptosis. Therefore, two additional questions arise: do cells that meet the conditions for ferroptosis also undergo apoptosis? Do cells that meet the conditions for pyroptosis, which are accompanied by elevated ROS, also exhibit apoptosis? This warrants investigation. In summary, ROS plays a significant role in cell death, and reducing ROS accumulation is crucial for improving the three types of cell death.

#### Crosstalk between apoptosis and ferroptosis

Apoptosis and pyroptosis are generally considered genetically programmed forms of cell suicide, whereas ferroptosis is attributed to metabolic dysregulation [4]. There is some crosstalk among these three modes of cell death. Studies have found that ferroptosis and apoptosis exhibit synergistic effects. As a key regulator of lipid redox signaling, nicotinamide adenine dinucleotide phosphate oxidase (NOX) has been shown to participate in the induction of apoptosis [290]. Similarly, ROS production mediated by NOX1, NOX2, and NOX4 also contributes to the initiation of ferroptosis by inducing lipid peroxidation [291]. Moreover, Bim and Bax activate the caspase-dependent apoptotic pathway and trigger the ferroptosis pathway by downregulating SLC7A11 and GPX4 [292]. In contrast, during bortezomib-induced apoptosis, ACSL4 is a caspase cleavage target [293], indicating that executioner caspases can suppress ACSL4-mediated incorporation of PUFAs into membranes, thereby limiting a cell’s capacity to undergo ferroptosis. Conversely, cells undergoing ferroptosis due to cystine deprivation exhibit intracellular GSH levels at approximately 10% of normal [294]. The reducing power of GSH may be required for the processing and activation of caspase-3 and caspase-8 [295]; thus, cells depleted of GSH may be unable to activate caspase-mediated apoptosis. This suggests that apoptosis and ferroptosis can mutually antagonize each other.

#### Crosstalk between ferroptosis and pyroptosis

On ferroptosis and pyroptosis. Studies have found that Ginsenoside Rh3 (GRh3) inhibits NRF2 nuclear translocation, leading to decreased expression of heme oxygenase-1, which in turn promotes the expression of NLRP3 and caspase-1; ultimately, caspase-1 activates GSDMD-dependent pyroptosis. Meanwhile, GRh3 prevents NRF2 from entering the nucleus, thereby suppressing SLC7A11, resulting in GSH depletion and the accumulation of iron, ROS, and MDA, ultimately triggering ferroptosis in CRC cells [296]. Similarly, in 40 segments of human coronary artery samples from 4 autopsy cases, proteins associated with both ferroptosis (PTGS2, ACSL4, GPX4) and pyroptosis (caspase-1, NLRP3) were detected concurrently, with PTGS2 occupying a hub position [297]. This suggests that ferroptosis and pyroptosis can occur simultaneously. In epilepsy, on the one hand, pyroptosis can promote the occurrence of ferroptosis; IL-1β produced following neuronal pyroptosis may exacerbate ferroptosis by influencing the expression of genes related to iron metabolism. On the other hand, loss of GPX4 (a key inhibitor of ferroptosis) enhances caspase-1- and caspase-11-mediated GSDMD cleavage, thereby promoting pyroptosis. The two not only coexist but also mutually reinforce each other [298]. However, some studies also indicate an inhibitory relationship between ferroptosis and pyroptosis. In a study of hepatocellular carcinoma, it was found that overexpression of 3-hydroxy-3-methylglutaryl-coenzyme A reductase (HMGCR) can inhibit ferroptosis while promoting pyroptosis, whereas HMGCR knockdown produced the opposite results [299].

#### Crosstalk between apoptosis and pyroptosis

There is also substantial research on apoptosis and pyroptosis. Studies suggest that apoptosis and pyroptosis can occur concurrently. In sepsis-induced cardiomyopathy, apoptosis and pyroptosis are not isolated events but share a common upstream regulatory pathway: the STING–IRF3–NLRP3 axis. Activated NLRP3 inflammasomes directly activate caspase-1, which cleaves GSDMD and generates mature IL-1β and IL-18, thereby executing the canonical pyroptotic program. Activated NLRP3 inflammasomes can also activate caspase-8, which in turn activates downstream caspase-3 to initiate apoptosis [256]. Although apoptosis and pyroptosis can occur simultaneously under certain conditions, which mode that predominates is substrate-dependent. Studies have shown that the nPD-L1/p-Stat3/GSDMC signaling axis regulates the interconversion between these two death modalities. Upon receiving a death signal (such as TNFα), when GSDMC expression is low, caspase-8 primarily activates the downstream apoptotic pathway. In contrast, when GSDMC expression is upregulated, it becomes the preferred substrate of caspase-8, and its cleavage products directly trigger pyroptosis, thereby “hijacking” the classical apoptosis-inducing signal [300].

#### Characteristics of apoptosis-, ferroptosis-, and pyroptosis-triggered responses

Intrinsic apoptosis is typically triggered by developmental cues or stress stimuli (e.g., nutrient deprivation, DNA damage, and endoplasmic reticulum stress) [301]. When cells are exposed to certain death ligands or pathogen-associated molecular patterns (PAMPs), their response can escalate from pro-inflammatory and pro-survival gene expression to extrinsic apoptosis or programmed necrosis if there is an immediate threat, manifested as damage to key signaling components [4]. Pairings of these ligands with death receptors include: FAS ligand (FASL) with FAS; tumor necrosis factor (TNF) or lymphotoxin-α with TNF receptor 1 (TNFR1); TNF-like cytokine 1A (TL1A) with death receptor 3 (DR3); and TNF-related apoptosis-inducing ligand (TRAIL) with TRAILR1 or TRAILR2 (mice have only a single TRAILR) [302].

Unlike apoptosis, the assembly of inflammasomes that trigger pyroptosis is initiated by proteins that sense specific DAMPs or PAMPs and subsequently form an oligomeric scaffold. Some “sensor” proteins bind DAMPs or PAMPs directly, such as AIM2, which recognizes viral or bacterial double-stranded DNA [303, 304]. Members of the NLR family apoptosis inhibitory proteins (NAIPs) bind bacterial flagellin, needle, and inner rod proteins [305, 306]. NLR family pyrin domain-containing protein 1 (NLRP1) and caspase activation and recruitment domain 8 (CARD8) can detect certain infections because each harbors an inhibitory domain that can be modified by bacterial or viral enzymes and targeted for proteasomal degradation [307]. Removal of this inhibitory domain promotes inflammasome assembly. In addition, inflammasome sensors that indirectly detect PAMPs or DAMPs include PYRIN and NLRP3. PYRIN senses bacterial toxins that modify and inactivate Rho GTPases, and PYRIN dephosphorylation and microtubule polymerization are thought to participate in inflammasome assembly [308]. NLRP3 responds to a variety of stimuli, including the bacterial toxin nigericin, extracellular ATP, and uric acid crystals associated with gout [309].

Ferroptosis can be triggered by physiological conditions, such as high concentrations of extracellular glutamate. From an evolutionary perspective, incorporating PUFAs into cellular membranes is crucial for the development of complex neural circuits, the regulation of membrane fluidity, and cellular adaptation to varying temperature environments [310]. The accumulation of PUFAs in membranes renders them vulnerable to lethal lipid peroxidation because they readily form stabilized free radicals; this process generates numerous reactive electrophiles that target nucleophilic sites on key proteins. Therefore, one possible physiological function of ferroptosis is to eliminate cells that excessively produce electrophilic intermediates [311].

The outcome of cell death depends not only on the triggering stimulus but also on cellular metabolism and cell type. For example, tumor cells in a mesenchymal-like state are often resistant to apoptosis induced by standard therapies. In such cells, elevated levels of PUFA-PLs increase reliance on GPX4 to eliminate lipid peroxides for survival, rendering them highly susceptible to ferroptosis [312]. Clear cell renal cell carcinoma, triple-negative breast cancer cells, gastric cancer, glioma, and bladder cancer are likewise prone to ferroptosis due to their high levels of PUFA-ePLs [313]. In macrophages, inflammasomes can engage both caspase-1 and caspase-8, but pyroptosis typically predominates because of its faster kinetics [4]. In addition, we have observed that pyroptosis is more common in sepsis, sepsis-associated conditions, and inflammatory diseases.

Now we can address the two questions raised in the “Exploring the crosstalk mechanisms of apoptosis, ferroptosis, and pyroptosis” section. For the question, “When intracellular ROS levels rise beyond the limits of intrinsic compensation, which of the three modes of cell death will occur?” our answer is that it depends on the triggering conditions and metabolic state at the time, as well as the cell type. For the question, “Do cells that meet the conditions for ferroptosis also undergo apoptosis simultaneously? If cells that meet the conditions for pyroptosis also exhibit elevated ROS, do they concurrently undergo apoptosis?” based on prior findings, we consider these conjectures to be theoretically valid. However, mutual inhibition has been observed between ferroptosis and apoptosis, which may give rise to special cases. Moreover, if two modes of cell death occur concurrently, it remains important to determine the proportion each contributes to overall cell death, and whether they are linked by shared regulatory factors or are sequentially activated in a temporal order. These issues warrant an in-depth investigation to inform the development of therapeutics targeting the co-regulation of cell death pathways. In studies examining multiple modes of death, we recommend focusing on the “temporal effects” between modes of death, which may involve patterns of crosstalk among these modes.

### The role of NAT10 and P53 in the regulation of apoptosis, ferroptosis, and pyroptosis acetylation

From the regulatory role of acetylation in cell death modes, we found that NAT10 and P53 are involved in the regulation of apoptosis, ferroptosis, and pyroptosis [195, 222, 256]. We suspect that both play a significant role in the crosstalk of acetylation governing these three types of cell death.

NAT10 is a multifunctional enzyme that plays a crucial role in RNA acetylation. As the only known acetyltransferase in eukaryotes, its structure includes an acetyltransferase domain, a tRNA-binding domain, and an RNA helicase domain [314]. The RNA helicase domain primarily participates in the processing and assembly of 18S rRNA, while mutations in the acetyltransferase domain can completely inhibit the acetylation of 18S rRNA and RNA [315, 316]. Therefore, NAT10 can catalyze the Ac4C modification in tRNA, rRNA, and mRNA to regulate RNA stability, translation efficiency, and gene expression [317]. Interestingly, NAT10 not only enhances the stability and translation efficiency of pro-apoptotic RNA but also increases the stability and translation efficiency of anti-apoptotic RNA [318]. For example, NAT10 boosts the expression of the anti-apoptotic protein Bcl-2 while downregulating the expression of the pro-apoptotic proteins Bax and Bak [319]. However, NAT10-mediated acetylation of the N4-acetylcytidine (ac4C) transcription factor EC (Tfec) mRNA transcript promotes the expression of the BIK gene, facilitating apoptosis [195]. Therefore, while NAT10 primarily enhances RNA stability and translational efficiency, its role in regulating modes of cell death warrants close attention to its downstream targets. As a key regulator of apoptosis and ferroptosis, p53 is simultaneously promoted by NAT10 through the Mybbp1a–p53 axis to drive both apoptosis and ferroptosis [320]. Other studies have shown that p53 directly activates GSDME transcription to induce pyroptosis [321]. These findings indicate that p53 is a crosstalk target through which NAT10 regulates apoptosis, ferroptosis, and pyroptosis. NAT10 also modulates cell death pathways via metabolic reprogramming. For example, NAT10 regulates fatty acid metabolism and redox balance through ACOT7 or FSP1, where the generation of lipid peroxidation products (e.g., MDA) not only marks ferroptosis but may also activate the inflammasome and promote pyroptosis [226, 322]. In addition, shared inflammatory pathways are crucial to how NAT10 controls cell death modalities. The STING–NLRP3 pathway is central to pyroptosis but has also been shown to interact with ferroptosis: its activation may increase ROS production and promote ferroptosis, while DAMPs released during ferroptosis may activate NLRP3 and trigger pyroptosis [257, 323]. Upstream regulation of NAT10 also significantly influences its control over cell death, as the promoter activity of NAT10 is enhanced during DNA damage induced by H2O2 and cisplatin [324], and the upregulation of NAT10 can also be attributed to NAD^+^ depletion [325].

Regarding the regulation of cell death modalities by acetylation of p53, some studies indicate that both the specific acetylation sites and the number of acetylation sites are involved. Using ferroptosis as an example, the mouse p53 mutant with substitutions at K117, K161, and K162 (hereafter referred to as p53 3KR/3KR) can activate ferroptosis and suppress tumor growth; however, adding a fourth mutation at K98 abolishes the promotion of ferroptosis; further adding a fifth mutation at K136 completely diminishes the remaining tumor-suppressive function of p53. These findings underscore the importance of both the modification sites and their number in determining protein function [326]. In future studies, it is preferable to use mass spectrometry to achieve both qualitative and quantitative analysis of acetylation, enabling a deeper investigation of its underlying mechanisms. Given that a single protein may have multiple acetylation sites, research focusing on specific sites should employ custom site-specific antibodies rather than pan-acetylation antibodies to ensure the reliability of the results.

### The role of microtubule acetylation in regulating apoptosis and senescence

Similarly, we found that the acetylation of microtubules occurs simultaneously in the regulation of both apoptosis and pyroptosis. Microtubules are dynamic, polarized, hollow tubular polymers that serve as the “scaffolding” of the cell, as well as its “highways” and “motors,” being indispensable in cell division, endocytosis and exocytosis, organ development, and motility functions [327]. Microtubule-targeting agents (MTPAs), particularly paclitaxel and docetaxel, play a central role in the treatment of various human epithelial cancers; however, their effects on microtubules and the cell cycle have triggered molecular signals in the mitochondrial apoptosis pathway [328]. As the “highway” for transport, it plays a crucial role in promoting NLRP3 inflammasome assembly during pyroptosis [282]. Known as the “tubulin code,” post-translational modifications (PTMs) of tubulin significantly regulate microtubule functions in subcellular compartments for specialized cellular activities [329]. Acetylation plays an important role as a post-translational modification of proteins in microtubules, with acetylated microtubules long regarded as stable and long-lived. However, until recently, there has been limited information on whether the longevity of these microtubules is a cause or an effect of acetylation. Current advances indicate that this post-translational modification contributes to the ability of the microtubule lattice to withstand mechanical stress, thereby promoting the self-repair of microtubules [330, 331]. Research has found that the degree of microtubule acetylation affects cellular outcomes to a certain extent. Initially, acetylation of α-tubulin triggers autophagy; however, when the level of α-tubulin acetylation reaches a certain threshold, the cell undergoes apoptosis instead of autophagy. This suggests that the level of acetylated α-tubulin may determine the fate of the cell between survival and apoptosis [211]. Furthermore, the acetylation of microtubules is influenced by the presence of damaged sites at the microtubule ends and along the microtubule axis, allowing acetylation-related enzymes to enter through these locations. Studies have revealed that microtubule acetylation exhibits an exponential gradient distribution, which arises from strict regulation of the (de)acetylation processes of microtubules, proportional to the size of the cells. The control of axonal damage represents a mechanism for regulating post-translational modifications (PTMs) within microtubules by facilitating access through the lumen, including acetylation of the microtubules [332]. Therefore, studying the degree of microtubule acetylation and its acetylation sites may provide insights into the regulation of cell death.

In current research, acetylation at the K40 site of α-tubulin is the only post-translational modification located on the inner surface of microtubules, and this acetylation has been shown to be highly correlated with the occurrence of autophagy [333]. By contrast, acetylation at K252 of β-tubulin neutralizes the positive charge at that site, causing the tubulin heterodimer to adopt a conformation unfavorable for tubulin-tubulin interactions, thereby slowing the rate at which tubulin incorporates into microtubules [334]. The formation of microtubule structures can, on the one hand, provide tracks for autophagosome trafficking, and on the other hand, facilitate the interaction between ASC associated with mitochondria and NLRP3 associated with the endoplasmic reticulum, thereby triggering pyroptosis [335]. Apoptosis can also be mediated by microtubule acetylation, primarily through conformational changes that suppress microtubule dynamic instability and the G2/M phase transition of the cell cycle, ultimately activating mitochondrial apoptotic signaling [336]. However, the occurrence of apoptosis is more commonly observed in the context of anticancer drugs that act by altering microtubule acetylation status, and existing studies have not been refined to specific sites. In addition, investigators have identified acetylation sites on microtubules; beyond the previously reported K58, K103, K297, and K379, they have found K216, K324, K350, K362, and K392, although the specific functions of these sites await further experimental validation [337].

### The role of STAT3 and IL-1β in regulating ferroptosis and pyroptosis

STAT3 is a transcription factor that can interchange between the cytoplasm and nucleus, belonging to the STAT protein family (STAT1-6). As a core regulatory factor, STAT3 can be activated by various molecules, including specific cytokines, peptide ligands, growth factors, and products of oncogenes, thereby integrating multiple signaling pathways [338, 339]. In the cytoplasm, cytokine signals are responded to through tyrosine phosphorylation, leading to the activation of STATs. Once activated, STATs translocate to the nucleus, where they bind to specific DNA sequences and act as transcription factors [340]. These transcription factors play a crucial role in various physiological processes, including angiogenesis, cell proliferation, growth, and apoptosis [341]. In gastric cancer, STAT3 regulates ferroptosis primarily through its own histone acetylation, which increases its gene-level expression and promotes the expression of downstream direct targets GPX4, SLC7A11, and FTH1, thereby exerting an anti-ferroptotic effect [231]. In sepsis-induced acute lung injury, phosphorylated STAT3 recruits EP300, promoting acetylation of the histone region of the NLRP3 gene and thereby facilitating pyroptosis [254]. By comparison, in both diseases, inflammatory signals (such as LPS and cytokines) lead to sustained phosphorylation and acetylation-mediated activation of STAT3, yet the downstream pathways of STAT3 differ entirely, underscoring the crucial role of pathological context in directing the actions of regulatory factors.

IL-1β is a core member of the IL-1 family, encoded by the IL1B gene. It is synthesized intracellularly as a 31 kDa precursor form, pro-IL-1β, which is cleaved by Caspase-1, activated by inflammasomes (such as NLRP3, NLRC4, AIM2, etc.) into a 17 kDa mature form, and is subsequently released from the cell through “non-conventional secretion” [342]. In light of this, it is easy to understand how IL-1β regulates pyroptosis; however, the question of why IL-1β is also capable of regulating ferroptosis warrants further investigation. Research has found that, on one hand, IL-1β upregulates ACSL4 and TFRC, promoting the uptake of PUFA-PE and iron [343, 344], thus facilitating ferroptosis; on the other hand, the upregulation of IL-1β can stabilize iron-sulfur clusters, inhibit the expression of TFRC, and promote the transcription of FTH1, thereby maintaining iron homeostasis and inhibiting ferroptosis [229]. Notably, the upregulation of IL-1β leads to acetylation at lysine (K) 1042 of nicotinamide nucleotide transhydrogenase (NNT) (NNT K1042ac), ensuring the stability of iron-sulfur clusters. In summary, although IL-1β is not an acetylating enzyme, it can promote acetylation and regulate ferroptosis, which warrants further investigation.

### Combined interventions targeting multiple cell death pathways and their clinical efficacy

Evidence shows that combined interventions targeting multiple forms of cell death can significantly improve clinical efficacy. Immunogenic cell death (ICD) may be induced by tumor vaccination, radiotherapy, and certain types of chemotherapy [345]. Historically, ICD has also been referred to as immunogenic apoptosis, as most ICD events occur via apoptotic pathways. In most cancer types, the effectiveness of immune checkpoint inhibitors (ICIs) is substantially limited, with responses observed in only about one-third of patients [346]. Tumors resistant to ICIs are considered “cold” tumors [347]. However, Wang et al. found that ICIs can efficiently kill cold tumor cells only when pyroptosis is simultaneously induced. Likewise, inducing pyroptosis alone fails to elicit effective tumor suppression, underscoring the importance of combining pyroptosis inducers with ICIs for the treatment of cold tumors [348, 349]. Artesunate has been reported to induce necroptosis-like apoptosis and ferroptosis in tumor cells, and multiple clinical trials have documented the benefits of artesunate as monotherapy or in combination with other anticancer agents for cancer treatment [350, 351]. This likewise supports the clinical feasibility of combined interventions that do not rely on a single mode of cell death.

A potential therapeutic target in oncology is histone deacetylase (HDAC) inhibitors. These inhibitors remove acetyl groups from histone and non-histone proteins, ultimately downregulating gene transcription [352]. The FDA has approved HDAC inhibitors, including SAHA, romidepsin (depsipeptide), and belinostat, for certain cancers such as T-cell lymphoma. Panobinostat (LBH589) has been approved for the treatment of multiple myeloma. In addition, sulforaphane is an HDAC inhibitor used to treat colorectal cancer that can inhibit tumor progression by targeting HDAC1 and HDAC2 [353, 354]. Acetylation modifications regulate multiple forms of cell death; deeply probing the underlying mechanisms and identifying specific modification sites will offer a promising path for the development of new therapeutics that co-target more than a single mode of cell death.

## Future prospects

Although this study focuses on acetylation, it is essential to recognize that the complexity of cellular signaling networks relies on both the synergy and antagonism among multiple PTMs. For example, phosphorylation introduces negative charges and therefore appears superficially similar to acetylation, which removes positive charges, in terms of altering a protein’s electrostatic properties. However, because of their fundamentally different chemical kinetics and mechanisms of specific recognition, the two are not functionally equivalent. More importantly, competition among acetylation, ubiquitination, and SUMOylation for the same lysine residues, together with the complementary or antagonistic functional interplay with methylation, collectively shapes a finely tuned “PTM crosstalk” network. Future studies that concurrently profile multiple PTMs across different cell death models will be critical for a comprehensive understanding of cell fate decisions.
